# Conjugation to a transferrin receptor 1-binding Bicycle peptide enhances ASO and siRNA potency in skeletal and cardiac muscles

**DOI:** 10.1093/nar/gkaf270

**Published:** 2025-04-10

**Authors:** Michael E Østergaard, Michele Carrer, Brooke A Anderson, Megan Afetian, Mohsen A Bakooshli, Jinro A Santos, Stephanie K Klein, Juliana Capitanio, Graeme C Freestone, Michael Tanowitz, Rodrigo Galindo-Murillo, Hans J Gaus, Chrissa A Dwyer, Michaela Jackson, Paymaan Jafar-nejad, Frank Rigo, Punit P Seth, Katherine U Gaynor, Steven J Stanway, Liudvikas Urbonas, Megan A St. Denis, Simone Pellegrino, Gustavo A Bezerra, Michael Rigby, Ellen Gowans, Katerine Van Rietschoten, Paul Beswick, Liuhong Chen, Michael J Skynner, Eric E Swayze

**Affiliations:** Ionis Pharmaceuticals Inc., 2855 Gazelle Court, Carlsbad, CA 92010, United States; Ionis Pharmaceuticals Inc., 2855 Gazelle Court, Carlsbad, CA 92010, United States; Ionis Pharmaceuticals Inc., 2855 Gazelle Court, Carlsbad, CA 92010, United States; Ionis Pharmaceuticals Inc., 2855 Gazelle Court, Carlsbad, CA 92010, United States; Ionis Pharmaceuticals Inc., 2855 Gazelle Court, Carlsbad, CA 92010, United States; Ionis Pharmaceuticals Inc., 2855 Gazelle Court, Carlsbad, CA 92010, United States; Ionis Pharmaceuticals Inc., 2855 Gazelle Court, Carlsbad, CA 92010, United States; Ionis Pharmaceuticals Inc., 2855 Gazelle Court, Carlsbad, CA 92010, United States; Ionis Pharmaceuticals Inc., 2855 Gazelle Court, Carlsbad, CA 92010, United States; Ionis Pharmaceuticals Inc., 2855 Gazelle Court, Carlsbad, CA 92010, United States; Ionis Pharmaceuticals Inc., 2855 Gazelle Court, Carlsbad, CA 92010, United States; Ionis Pharmaceuticals Inc., 2855 Gazelle Court, Carlsbad, CA 92010, United States; Ionis Pharmaceuticals Inc., 2855 Gazelle Court, Carlsbad, CA 92010, United States; Ionis Pharmaceuticals Inc., 2855 Gazelle Court, Carlsbad, CA 92010, United States; Ionis Pharmaceuticals Inc., 2855 Gazelle Court, Carlsbad, CA 92010, United States; Ionis Pharmaceuticals Inc., 2855 Gazelle Court, Carlsbad, CA 92010, United States; Ionis Pharmaceuticals Inc., 2855 Gazelle Court, Carlsbad, CA 92010, United States; Bicycle Therapeutics, Portway Building, Granta Park, Cambridge CB21 6GS, United Kingdom; Bicycle Therapeutics, Portway Building, Granta Park, Cambridge CB21 6GS, United Kingdom; Bicycle Therapeutics, Portway Building, Granta Park, Cambridge CB21 6GS, United Kingdom; Bicycle Therapeutics, Portway Building, Granta Park, Cambridge CB21 6GS, United Kingdom; Bicycle Therapeutics, Portway Building, Granta Park, Cambridge CB21 6GS, United Kingdom; Bicycle Therapeutics, Portway Building, Granta Park, Cambridge CB21 6GS, United Kingdom; Bicycle Therapeutics, Portway Building, Granta Park, Cambridge CB21 6GS, United Kingdom; Bicycle Therapeutics, Portway Building, Granta Park, Cambridge CB21 6GS, United Kingdom; Bicycle Therapeutics, Portway Building, Granta Park, Cambridge CB21 6GS, United Kingdom; Bicycle Therapeutics, Portway Building, Granta Park, Cambridge CB21 6GS, United Kingdom; Bicycle Therapeutics, Portway Building, Granta Park, Cambridge CB21 6GS, United Kingdom; Bicycle Therapeutics, Portway Building, Granta Park, Cambridge CB21 6GS, United Kingdom; Ionis Pharmaceuticals Inc., 2855 Gazelle Court, Carlsbad, CA 92010, United States

## Abstract

Improving the delivery of antisense oligonucleotides (ASOs) and small interfering RNAs (siRNAs) to skeletal and cardiac muscles remains a pivotal task toward the broader application of oligonucleotide therapeutics. The targeting of myofibers and cardiomyocytes via conjugation of ASOs and siRNAs to ligands that bind the human transferrin receptor 1 (TfR1) has gathered significant interest in recent years. However, the selection of ligands with low molecular weight and optimal biophysical and binding properties is crucial to maximize the potential of the TfR1 ligand-conjugated antisense (LICA) technology. Here, through effective combination of phage display and peptide medicinal chemistry, we identified and characterized a bicyclic peptide (Bicycle^®^ molecule BCY17901), with a molecular weight of ∼2 kDa, that binds human TfR1 with high affinity and specificity. Conjugation to BCY17901 improved ASO and siRNA potency in skeletal and cardiac muscles of human TfR1 knock-in mice, after either intravenous or subcutaneous administration. Furthermore, single-nucleus RNA sequencing showed that conjugation to BCY17901 enhanced ASO activity in myonuclei of different muscle fiber types. Importantly, we demonstrated good translatability of our TfR1-targeting platform in skeletal and cardiac muscles of nonhuman primates. Our results offer great promise toward potential future applications of low-molecular-weight Bicycle LICA therapeutics for the treatment of diseases affecting skeletal muscle and heart.

## Graphical Abstract

**Graphical Abstract:**
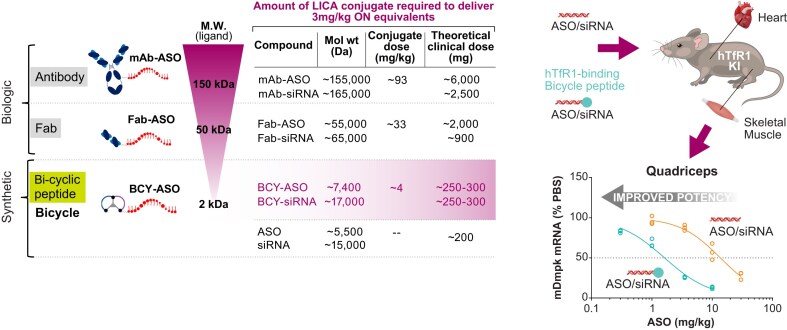

## Introduction

Antisense oligonucleotides (ASOs) and small interfering RNAs (siRNAs) are oligonucleotide (ON) drugs that primarily function by recruiting a functional enzyme (RNase H1 for ASOs and Ago2 for siRNAs), resulting in cleavage of complementary RNA [1]. Multiple ASOs and siRNAs have been approved for the treatment of human diseases [2, 3]. However, ASOs and siRNAs that function through an RNA cleavage mechanism have only been approved for the treatment of liver disorders (via systemic administration), eye, and central nervous system diseases (via local administration) [3]. ASOs administered to animals distribute broadly and show activity in most tissues after systemic dosing [4], while siRNAs have low bioavailability and therefore low activity in all tissues. However, using receptor-mediated uptake improves activity relative to unconjugated ASOs/siRNAs, as shown for GalNAc-conjugated ASOs/siRNAs targeting hepatocytes in the liver [5, 6]. In skeletal muscle and heart, the anticipated dose levels required for unconjugated ASOs to reach therapeutically meaningful activity are very high. Recently, the use of transferrin receptor 1 (TfR1) ligands has been shown to improve ON drug potency in skeletal and cardiac muscles via ligand-conjugated antisense (LICA) strategies. In 2016, it was first reported that a Fab′ antibody fragment binding mouse TfR1 increased siRNA activity in mice when administered as a conjugate [7]. More recently, it has been shown that ASO or siRNA conjugation to antibodies that bind mouse or human TfR1 resulted in large improvements in ON potency in mice and nonhuman primates (NHPs) [8]. While proof of concept for the delivery of ON drugs to skeletal muscle using anti-TfR1 antibody conjugates is now well established, there are multiple potential challenges to the use of antibodies and their fragments for LICA purposes: (i) off-target effects—anti-TfR1 antibodies can cause anemia and TfR1 depletion [9, 10]; (ii) synthetic complexity—antibodies are made by a biological manufacturing process and site-specific conjugation to antibodies and control of drug:antibody ratio can be complex, especially when using highly negatively charged ON drugs [11, 12]; (iii) size of ON drug conjugate—the antibody can comprise over 90% of the total ON drug mass, which for a clinical setting will require much larger volumes to be administered; (iv) tolerability—conjugating ON drugs to proteins increases the possibility of producing anti-drug antibodies; and (v) ON drugs conjugated to antibodies are dosed intravenously, while use of smaller ligands makes subcutaneous administration possible. Despite these challenges, multiple anti-human TfR1 antibody:ON conjugates have entered the clinic [13]. However, because of the aforementioned problems, the use of smaller targeting ligands offers advantages that can potentially improve the overall LICA product profile.

Bicycle molecules are ∼2-kDa bicyclic peptides formed through the structural constraint of peptides around a trimeric scaffold via formation of thioether cysteine linkages [14, 15]. They have attractive drug-like properties, such as high affinity and selectivity for their target, high plasma stability, tunable pharmacokinetics, and ease of manufacturing through chemical synthesis [16, 17]. Bicycle molecules are identified using modified phage display, where phage libraries expressing initially linear peptides are cyclized with a trifunctionalized small molecule “scaffold” via three cysteine residues, whose variable placement defines the loop sizes (specific combinations of loop lengths describing a distinct Bicycle “format”). The variable amino acid loop lengths, the scaffold moieties, and the amino acid residues all contribute to the large diversity of Bicycle libraries that can be screened to identify specific binders. Using this technology, Bicycle molecules were identified and optimized to bind human TfR1 with high affinity and specificity. The selected human TfR1-targeting Bicycle peptides were conjugated to ON moieties to examine their ability to improve ASO and siRNA activity in skeletal and cardiac muscles of animals. We demonstrated a good correlation between high-affinity human TfR1-binding bicyclic peptides and gene knockdown efficiency in muscles of the corresponding ON conjugates. Furthermore, the improved activity observed in mouse translated into good activity in NHPs.

## Materials and methods

### Synthesis of Bicycle peptides

Identification of the TfR1 Bicycle binders took place via iterative phage selections (see Supplementary Materials and Methods; Supplementary Fig. S1). Bicycle (BCY) peptides were then synthesized as previously described [16]. Briefly, Bicycle peptides were synthesized on Rink amide resin using standard 9-fluorenylmethyloxycarbonyl (Fmoc) solid-phase peptide synthesis, either by manual coupling (for large scale) or using a Biotage Syro II automated peptide synthesizer (for small scale). Following trifluoroacetic acid cleavage from the resin, peptides were precipitated with diethyl ether and dissolved in 50:50 acetonitrile/water. The crude peptides (at ∼1 mM concentration) were then cyclized with 1.3 equiv. of 1,1′,1″-(1,3,5-triazinane-1,3,5-triyl)tris(2-bromoethanone) (TATB) scaffold, using ammonium bicarbonate (100 mM) as a base. Completion of cyclization was determined by matrix-assisted laser desorption ionization time-of-flight (MALDI-TOF) or liquid chromatography–mass spectrometry (LC–MS). Once complete, the cyclization reaction was quenched using *N*-acetyl cysteine (10 equiv. with respect to TATB), and the solutions were lyophilized. The residue was dissolved in an appropriate solvent and purified by reverse-phase high-performance liquid chromatography (HPLC). Peptide fractions of sufficient purity and correct molecular weight (verified by either MALDI-TOF and HPLC or LC–MS) were pooled and lyophilized. Concentrations were determined by ultraviolet absorption using the extinction coefficient at 280 nm, which was based on Trp/Tyr content. Standard Fmoc amino acids, as well as nonproteinogenic Fmoc amino acids, were obtained from Sigma–Aldrich, Iris Biotech GmbH, Apollo Scientific, ChemImpex, and Fluorochem.

### Solid-phase synthesis of amino-modified ASOs and siRNAs

ONs were synthesized on 40–250 μmol scale on an AKTA Oligopilot 10 using a NittoPhase UnyLinker solid support (400 μmol/g) or a polystyrene support loaded with a C6 amine linker (300 μmol/g). An 4-monomethoxytrityl (MMT)-protected hexylamino phosphoramidite was used for incorporation of an amine at the 5′-end of the ON. Fully protected nucleoside phosphoramidites were incorporated using standard solid-phase ON synthesis conditions, i.e. 3% dichloroacetic acid in dichloromethane for deblocking, 1 M 4,5-dicyanoimidazole and 0.1 M *N*-methylimidazole in acetonitrile (MeCN) as activator, 10% acetic anhydride in tetrahydrofuran (THF) and 10% *N*-methylimidazole in THF/pyridine for capping, 0.05 M iodine in 9:1 pyridine:H_2_O for phosphodiester oxidation, and 0.1 M xanthane hydride in 3:2 pyridine:acetonitrile (v/v) for thiolation. All phosphoramidites were dissolved at 0.1 M in 1:1 MeCN:toluene (v/v) and incorporated with 3 min coupling times for DNA phosphoramidites and 12 min coupling times for all other phosphoramidites. After conclusion of the synthesis, the protecting groups were removed, and the ONs were cleaved from the resin by treating with 20% diethylamine in toluene for 20 min, followed by suspending the solid support in aqueous concentrated ammonia at 55°C overnight for ASOs or at room temperature for 48 h for siRNAs. The support was removed by filtration and the crude ON was purified using strong anion-exchange chromatography (SAX) (Buffer A: 100 mM NH_4_OAc in 3:7 ACN:H_2_O; Buffer B: 100 mM NH_4_OAc, 1.5 M NaBr in 3:7 ACN:H_2_O). Purified ONs were then desalted on C18 reverse-phase HPLC and lyophilized. ONs synthesized: Dmpk (dystrophia myotonica protein kinase) is a full phosphorothioate (PS) 3-10-3 cEt gapmer with the sequence 5′-ACAATAAATACCGAGG-3′, and Malat1 (metastasis-associated lung adenocarcinoma transcript 1) is a full PS 3-10-3 cEt gapmer with the sequence 5′-GCATTCTAATAGCAGC-3′. The Hprt siRNA is a duplex where the antisense strand is full 2′-OMe RNA, except for position 1, which is a 2′-*O*-methoxyethyl vinylphosphonate, and positions 2, 6, 12, and 14, which use a 2′-F RNA nucleotide. The sense strand is a full 2′-OMe RNA, except for positions 7, 9, 10, and 11, which use a 2′-F RNA nucleotide. The sequence of the antisense strand of the Hprt siRNA is 5′-TUAAAAUCUACAGUCAUAGGAAU-3′, and the sequence of the sense strand is 5′-UCCUAUGACUGUAGAUUUUAA-3′. ASOs were modified with a hexylamine at the 5′-end, while the siRNA sense strand was modified at the 3′-end with a C6 amino linker.

### Synthesis of BCN-ONs and conjugation to bicyclic peptides

Amino-modified ON strands were dissolved to 50 mg/ml in 100 mM sodium tetraborate (pH 8.5), followed by the addition of 2.0–3.0 equiv. BCN-NHS carbonate dissolved in *N*,*N*-dimethylformamide (DMF). After reaction completion and verification via LC–MS analysis, ONs were purified using SAX and desalted by reverse phase as detailed earlier. BCN-conjugated ONs were dissolved to 50 mg/ml in H_2_O with 10% 0.1 M sodium tetraborate buffer (pH 8.5), followed by the addition of 1.5 equiv. Bicycle peptide-azide dissolved at 20 mg/ml in DMF. After reaction completion and verification via LC–MS analysis, strands were purified and desalted as described earlier.

### Generation and dosing of human TfR1 knock-in mice

The human TfR1 knock-in (KI) mouse was generated using an approach similar to the one reported by Yu *et al.* [18]. Briefly, CRISPR/Cas9-mediated gene editing was used to replace the coding region of mouse exon 2 and the splice donor site of mouse intron 2 with the human TfR1 open reading frame. The gene targeting strategy was based on NCBI transcripts NM_011638.4 (mouse Tfrc) and NM_001128148.2 (human TFRC). The resulting KI mouse expresses the human TFRC gene under the control of the endogenous mouse promoter. Experiments in adult C57BL/6NTac and human TfR1 KI mice were performed at Ionis Pharmaceuticals. Mice were divided into groups of three or four and acclimated for at least 7 days prior to dosing. Food and water were available ad libitum in a virus-free barrier facility with a 12-h light/dark cycle. The mice were dosed by either intravenous or subcutaneous injection as specified in the text and figures. The mice received PBS (vehicle control), unconjugated ON, or OKT9 Fab′-conjugated or Bicycle-conjugated ON, as listed in the text and figures. For mouse siRNA studies, a group of animals received either unconjugated siRNA or lipid (palmitic acid, C16)-conjugated siRNA as a reference compound. Each figure lists the dose level at which the compounds were administered. Doses are expressed in mg/kg body weight, and they refer to the ASO or siRNA portion of the molecules (ASO or siRNA equivalents). Mice were dosed on days 1, 8, and 15, and sacrificed 1 week after the last dose (day 22), except for Supplementary Fig. S10B, where mice were dosed on days 1 and 8, and sacrificed 3 weeks after the second dose (day 29). The experiments in C57BL/6NTac and human TfR1 KI mice were approved by the institutional animal care and use committee (IACUC) at Ionis Pharmaceuticals. All procedures in C57BL/6NTac and human TfR1 KI mice were conducted following the eighth edition of the Guide for the Care and Use of Laboratory Animals (AAALAC-accredited Unit 000962 and State of California Department of Public Health Certificate No. 071), and conducted under IACUC protocol 2023-1219.

### Evaluation of Bicycle–ON conjugates in NHPs

Two- to four-year-old female cynomolgus monkeys (*Macaca fascicularis*) were randomized and assigned to treatment groups (*N* = 3 per group). Bicycle-conjugated ASOs or siRNAs were administered to the animals at 25 mg/kg, via intravenous infusion over 1 h (10 ml/kg infusion volume). The animals were dosed on days 1, 8, and 15 (three total doses), and sacrificed 13 days after the last dose (day 28). Tissues were harvested, weighed, and either flash frozen (for RNA analysis) or formalin-fixed (for histological analysis). The NHP study was conducted at the Korea Institute of Toxicology (KIT), and it was approved by the institutional animal care and use committee at KIT.

### Animal tissue homogenization, RNA isolation, and quantitative real-time polymerase chain reaction

For mouse studies, skeletal muscle, heart, and liver tissues were collected and flash frozen in liquid nitrogen. For NHP skeletal muscle (quadriceps) and heart, 4-mm-thick slices were prepared from the dissected tissues, and immediately frozen. At the time of sampling, three biopsy punches were collected from each of the frozen muscle slices. Punches were taken from three distinct areas of the frozen tissue to avoid geographical bias. Contaminating fat tissue and skin were also avoided as much as possible. Total RNA was extracted from each of the three punches. For NHP liver, a piece of tissue weighing ∼500 mg was collected and flash frozen in liquid nitrogen. Tissues were homogenized in TRIzol reagent (Thermo Fisher Scientific, Cat. 15596026). Total RNA was isolated by phenol–chloroform extraction, followed by purification using an RNeasy 96 Kit (Qiagen, Cat. 74181). The Express One-Step RT-qPCR Kit (Thermo Fisher Scientific, Cat. 11781200) was used to measure the RNA level of target genes by quantitative real-time polymerase chain reaction (RT-qPCR) in each sample. To determine knockdown of the target genes, their expression levels were normalized to housekeeping genes or total RNA input, and data were represented as percent change in expression relative to control groups. Additional details about the protocols used for RT-qPCR analysis are included in the Supplementary Materials and Methods. Supplementary Table S1 lists the sequences of primer–probe sets used in RT-qPCR assays.

### Statistical analysis

GraphPad Prism software was used to calculate ED_50_ values and perform one-way analyses of variance or Dunnett’s multiple comparison statistical tests. *P*-values <.05 were considered statistically significant and labeled with an asterisk (*) in the figures. Error bars indicate the standard deviation.

Differential expression analysis of single-nucleus transcriptome data was performed using a hurdle model tailored to scRNA-seq data (MAST package) [19] with *P*-value adjustment based on Bonferroni correction using all genes in the dataset. Adjusted *P*-values <.001 were considered statistically significant and are indicated with asterisks (***) in the figures.

## Results

### Identification and characterization of human TfR1-binding Bicycle molecules

**Figure 1.**
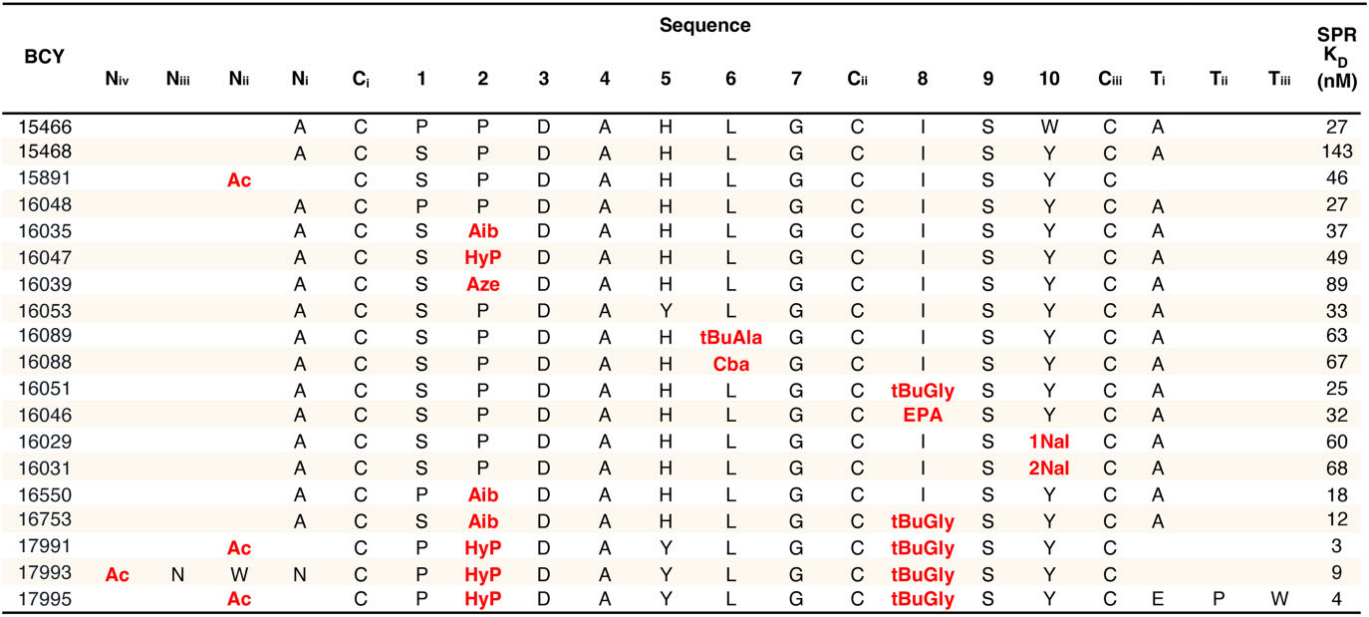
Use of non-natural substitutions and guidance from crystal structure to enhance the binding affinity of lead Bicycle peptides for human TfR1. Non-natural substitutions (shown in red in the table) were screened using surface plasmon resonance (SPR) against human TfR1. Aib, 2-aminoisobutyric acid; Aze, azetidine-2-carboxylic acid; HyP, hydroxyproline; tBuAla, *tert*-butylalanine; Cba, cyclobutylalanine; tBuGly, *tert*-butylglycine; EPA, 2-amino-3-ethylpentanoic acid; 2Nal, 3-(2-naphthyl)-alanine; 1Nal, 3-(1-naphthyl)-alanine.

Phage selection screens against human TfR1 identified Bicycle molecules that bound to the cognate receptor with sub-micromolar affinity (Supplementary Fig. S1). Subsequent phage display rounds were used to select Bicycle peptides that do not compete with the natural transferrin ligand. Affinity maturation of the lead Bicycle using natural amino acid substitutions was performed to further increase the potency (Fig. 1). From this exercise, two Bicycle molecules sharing a common motif were identified for further exploration, BCY15466 and BCY15468 (Fig. 1, top two rows).

Fluorescently labeled peptides were used to investigate internalization and localization of these hits within human TfR1-expressing cells. Bicycle peptide staining overlapped with TfR1 and the endosomal markers EEA1, RAB5, and RAB7 (Supplementary Fig. S2 and Supplementary Table S2). Pretreatment of cells with DYNGO-4A inhibited dynamin-dependent endocytosis of TfR1 and Bicycle peptide (Supplementary Fig. S3).

### Medicinal chemistry optimization of lead Bicycle ligand

The crystal structure of human His_6_-TfR1 (called hereafter human TfR1) in complex with the Bicycle molecule BCY15466 was solved at 2.3 Å resolution (Supplementary Fig. S4A–F and Supplementary Table S3) and used to optimize the binding of the Bicycle lead to TfR1 (see Supplementary Results). An alanine scan was further performed to determine core residues involved in the interaction with the human TfR1 protein (Supplementary Fig. S5) and solvent-facing residues that can be used to fine-tune the Bicycle molecular properties such as stability and solubility. Substitution of Ser1, Pro2, and His5 had minimal effect on binding, and the crystal structure showed them to be mostly solvent exposed. Alanine substitution of Leu6 resulted in a small negative effect on binding of ∼5-fold, as measured by fluorescence polarization (FP) (Supplementary Fig. S5), which can be explained by the loss of a set of weak intramolecular interactions with the backbone carbonyl of Asp3. Substituting alanine on any of the remaining positions resulted in significant loss of binding, especially Gly3 substitution was highly detrimental to binding because the presence of a glycine in this position permits the turn of loop 1 and is the only compatible residue, since the substitution to Ala or a different side chain would sterically clash with Ser159 of human TfR1. The crystal structure agreed well with the alanine scan data, and molecular interactions could be interrogated to assist in selecting amino acid substitutions to improve Bicycle properties.

Binding data (*K*_D_, measured by SPR) demonstrated that the removal of the alanines at the N-terminal and C-terminal was tolerated, along with capping the N-terminal with an acetyl group, as exemplified by BCY15891 (Fig. 1). Therefore, BCY15891 was advanced to plasma stability assessment in mouse, and it was found to be stable, having a plasma half-life of 22.7 h (Supplementary Table S4). Several invariant residues were identified from this exercise, namely Asp4, Gly7, and Ser9. It was observed in the phage campaign that in some cases a proline was swapped for the serine at position 2. Proline was indeed tolerated as indicated by BCY16048 (Fig. 1). As expected, a range of substituents were tolerated at Pro3, since this residue is solvent facing (Fig. 1). Affinity maturation also identified several swaps for His5, with tyrosine identified as a potential alternative (Fig. 1). Our results (data not shown) suggest that histidine may contribute to poor solubility; therefore, tyrosine was fixed at this position. *tert*-Butylalanine and cyclobutylalanine were identified as alternatives for the leucine at position 6 (Fig. 1 and Supplementary Fig. S6A). Correspondingly, *tert*-butylglycine and 2-amino-3-ethylpentanoic acid were identified as possible replacements for Ile7 (Fig. 1 and Supplementary Fig. S6A). Finally, 1-naphthyl and 2-napththyl were identified as alternative groups for Tyr10 (Fig. 1 and Supplementary Fig. S6A).

**Figure 2.**
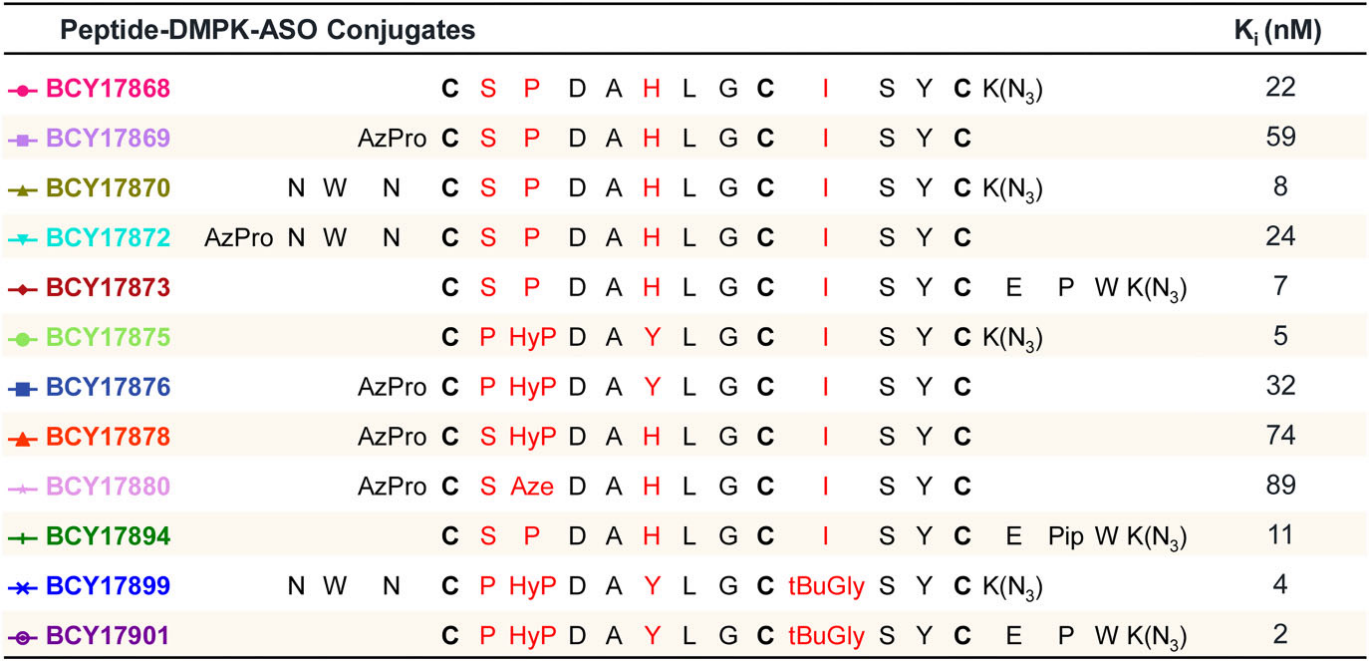
Twelve different Bicycle–Dmpk–ASO conjugates bind human TfR1 with different affinities. Bicycle peptide sequences and inhibition constants (*K*_i_) of Dmpk–ASO conjugates measured using bioluminescence resonance energy transfer (BRET). Modified positions are shown in red in the table.

A series of combinations was prepared to further improve binding affinity and *in vitro* stability. Combinations with N- and C-terminal extensions were also made as exemplified by BCY17993 and BCY17995. From these, single-digit nanomolar binders with excellent plasma stability, such as BCY17995, were identified (Fig. 1 and Supplementary Table S4). Finally, a series of analogues containing azidolysine [K(N_3_)] or azidopropanoic acid (AzPro) linker groups, which are suitable for conjugation of an ASO or siRNA, was prepared (Fig. 2 and Supplementary Fig. S6B). The K(N_3_) group was added to the C-terminus, while the AzPro group was added to the N-terminus of the Bicycle molecule (Fig. 2 and Supplementary Fig. S6B).

The Bicycle peptides were conjugated to ASOs and siRNAs (Supplementary Results, Fig. 2, and Supplementary Fig. S7). To screen for potential off-target peptide–protein binding interactions of the lead Bicycle peptide alone or the BCY–siRNA conjugate, the Retrogenix^®^ cell microarray technology was used. Here, the test articles were evaluated for binding to fixed HEK293 cells, individually overexpressing 6105 full-length human plasma membrane proteins, secreted, and cell surface-tethered human secreted proteins, plus a further 400 human heterodimers. In the Retrogenix assay, the Bicycle peptide interacted only with human TfR1, demonstrating the high degree of specificity of the lead ligand for the cognate receptor. Similarly, the BCY–siRNA conjugate bound human TfR1 and, to some extent, RNase1 (data not shown).

### *In vivo* screening of Bicycle peptides identifies ligands that enhance ASO activity in skeletal muscle and heart of human TfR1 KI mice

**Figure 3.**
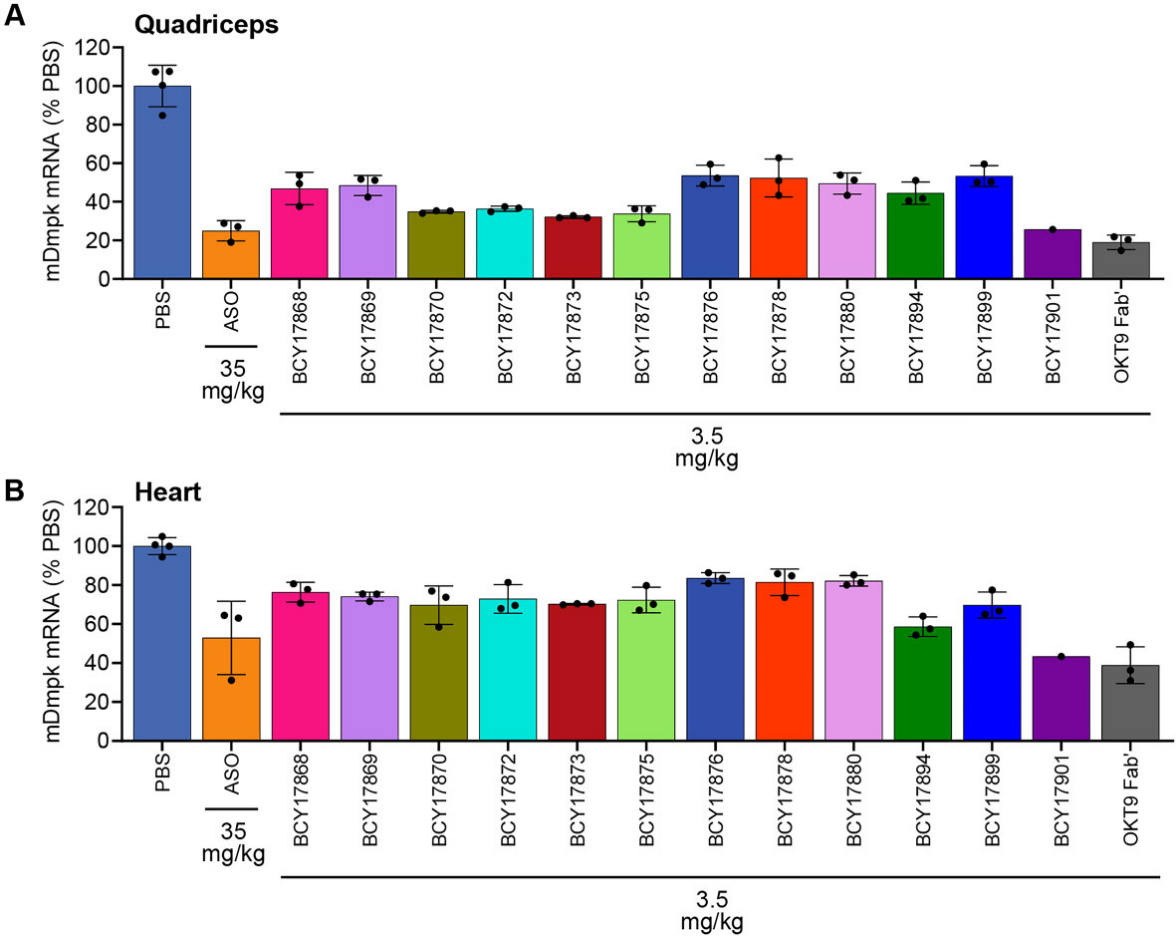
*In vivo* screening of Bicycle peptides that bind to human TfR1 with different affinities identifies BCY17901 as the lead ligand. Bicycle peptides were conjugated to mouse Dmpk ASO and dosed intravenously in human TfR1 heterozygote KI mice at 3.5 mg/kg ASO equivalent dose. A group of mice were dosed with the unconjugated Dmpk ASO at 35 mg/kg. Another group of mice was dosed with Dmpk ASO conjugated to the benchmark human TfR1 ligand OKT9 Fab′. The graphs report the mouse Dmpk (mDmpk) mRNA levels measured by RT-qPCR in (**A**) the quadriceps muscle and (**B**) the heart, expressed as percentage of the PBS-treated animals (vehicle control), after normalization using mouse Gapdh as the housekeeping gene. Error bars indicate standard deviation.

A KI mouse expressing the human TfR1 open reading frame under the control of the endogenous mouse Tfrc gene promoter was generated. Heterozygote KI mice express human TfR1 mRNA and protein in skeletal muscle and heart at robust levels (Supplementary Fig. S8A–D). The human TfR1 KI mouse was used to assess the enhancement of ON *in vivo* activity conferred by conjugation to selective human TfR1-targeting Bicycle molecules. Thus, 12 Bicycle peptide ligands were conjugated to well-characterized ASOs that target either mouse Dmpk mRNA (Fig. 3A and B) or the ubiquitously expressed mouse Malat1 RNA (Supplementary Fig. S9A and B). RT-qPCR was used to measure the target mRNA knockdown in quadriceps muscle and heart of human TfR1 KI heterozygote mice after three weekly doses of unconjugated ASO at 35 mg/kg (orange bar) or Bicycle-conjugated ASO at 3.5 mg/kg ASO equivalents. A group of control mice was dosed with PBS vehicle. Another group of mice received the Dmpk ASO conjugated to a benchmark ligand, the human TfR1-targeting OKT9 Fab′ at 3.5 mg/kg ASO equivalents (dark gray bar). Conjugation to the high-affinity Bicycle ligand BCY17901 provided the greatest enhancement in ASO activity versus unconjugated Dmpk or Malat1 ASO (orange bar, 10-fold higher dose), both in quadriceps muscle (Fig. 3A and Supplementary Fig. S9A) and in heart (Fig. 3B and Supplementary Fig. S9B), consistently across the two different ASO targets. Conjugation of the Dmpk ASO to the benchmark OKT9 Fab′ provided a similar activity improvement as the much smaller BCY17901 peptide (Fig. 3A and B). Consistent with the specificity of binding of BCY17901 to the human and not the murine TfR1 (Supplementary Table S5), the activity of BCY17901–Malat1 ASO conjugate was largely blunted in the skeletal and cardiac muscles of wild-type mice, compared to human TfR1 KI mice (Supplementary Fig. S10A). The *in vivo* screening exercise revealed that conjugation of ASOs to the C-terminal of Bicycle ligands that have low nM binding affinity for human TfR1 is optimal for efficient muscle targeting. Given the remarkable improvement in ASO activity in skeletal and cardiac muscles obtained by BCY17901 conjugation, we further characterized this Bicycle ligand in dose–response experiments.

### Conjugation of ASO and siRNA to the lead Bicycle peptide ligand BCY17901 results in robust, dose-responsive target knockdown and improved potency in skeletal and cardiac muscles of human TfR1 KI mice

**Figure 4.**
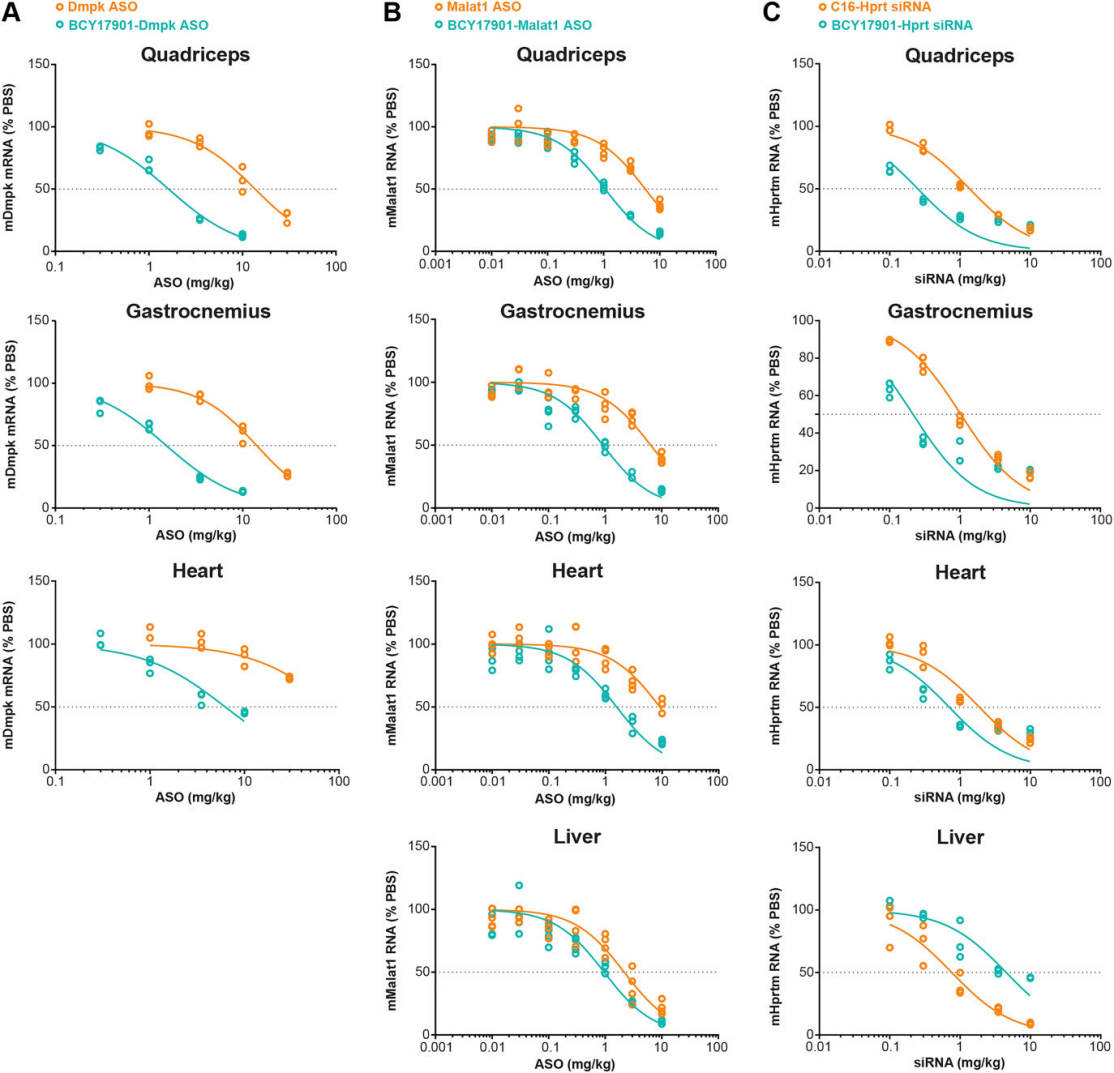
Conjugation of Dmpk ASO, Malat1 ASO, and Hprt siRNA to BCY17901 improves ON potency in skeletal muscle and heart of human TfR1 heterozygote KI mice. Dose-dependent target knockdown was measured by RT-qPCR in various skeletal muscle groups (quadriceps and gastrocnemius), heart, and liver of human TfR1 KI mice after intravenous injection of (**A**) Dmpk ASO, (**B**) Malat1 ASO, and (**C**) Hprt siRNA conjugated to BCY17901. Unconjugated ASOs and a lipid (palmitate, C16)-conjugated Hprt siRNA (orange) were included in the study to compare their potency versus the Bicycle-conjugated counterparts (green). Data are expressed as percentage target RNA level compared to PBS-treated (vehicle control) mice, after normalization using mouse Gapdh as housekeeping gene. Each open dot represents one animal. Doses refer to the ASO or siRNA component of the LICA molecules (ASO or siRNA equivalents).

To further validate the lead Bicycle ligand BCY17901, and to calculate the improvement in potency of the Bicycle conjugates over unconjugated ASO or lipid-conjugated siRNA molecules, we performed dose–response experiments in human TfR1 KI mice. Palmitate-conjugated siRNA was used as a control for the siRNA–Bicycle conjugate since an unconjugated siRNA shows very little activity and lipid-conjugated siRNAs have been shown to have activity in extrahepatic tissues [20]. We measured a robust increase in potency, and consequently lower ED_50_ values, for BCY17901-conjugated Dmpk ASO (Fig. 4A and Supplementary Table S6A) and BCY17901-conjugated Malat1 ASO (Fig. 4B and Supplementary Table S6B) compared to the respective unconjugated ASOs. The fold improvement in potency calculated based on ED_50_ values was 5.2–8.4 in quadriceps muscle, 6.9–7.6 in gastrocnemius muscle, and ≥5.8 in the heart (Supplementary Table S6A and B). Similar potency enhancements of 5.5-fold in quadriceps muscle, 4.7-fold in gastrocnemius muscle, and 2.7-fold in the heart of human TfR1 KI mice were calculated for BCY17901-conjugated Hprt siRNA compared to a lipid (palmitic acid, C16)-conjugated version of the same siRNA (Fig. 4C and Supplementary Table S6C). Unconjugated siRNAs are not robustly active *in vivo* at doses ≤10 mg/kg, and therefore were not included in the study. In addition, conjugation of Malat1 ASO and Hprt siRNA to BCY17901 enhanced ON potency compared to the unconjugated ASO or the C16-conjugated siRNA in diverse muscle groups, resulting in 2.9–3.9-fold improvement in soleus muscle, which is composed predominantly by type I slow-twitch myofibers, and 4.7–5.5-fold improvement in extensor digitorum longus (EDL) muscle, which is composed predominantly by type II fast-twitch myofibers (Supplementary Fig. S11A and B).

Importantly, immunohistochemical stain for human TfR1 in quadriceps muscle and heart from human TfR1 demonstrates no reduction in receptor protein levels on the surface of skeletal and cardiac muscle cells after repeated dosing with BCY17901–Malat1 ASO at the relatively high dose of 10 mg/kg ASO equivalents (Supplementary Fig. S12).

Systemic administration of BCY17901-conjugated Malat1 ASO in human TfR1 KI mice did not result in target RNA knockdown in central nervous system tissues, such as brain cortex and spinal cord, suggesting that the conjugated ASO does not cross the blood–brain barrier (BBB) (Supplementary Fig. S13).

Interestingly, in the liver, where TfR2 and not TfR1 is the predominant TfR homolog, the BCY17901–Malat1 ASO conjugate was only minimally (2.3-fold) more active than the unconjugated ASO (Fig. 4B and Supplementary Table S6B). Dmpk is lowly expressed in liver, so its mRNA level was not measured in the hepatic tissue. Strikingly, for the Hprt siRNA, conjugation to BCY17901 caused a 5.9-fold decrease in potency in the liver compared to the same siRNA conjugated to C16 (Fig. 4C, Supplementary Table S6C, and Supplementary Fig. S10B). The same effect was less noticeable for the Malat1 ASO, likely due to the many phosphorothioate linkages that promote nonspecific protein binding and liver accumulation, independent of the TfR1-mediated mechanism (Fig. 4B, Supplementary Table S6B, and Supplementary Fig. S10A). The partial liver-sparing findings were confirmed in a separate study where the Hprt siRNA was conjugated to either another high-affinity human TfR1-targeting Bicycle ligand, BCY17873, or a benchmark human TfR1 ligand, OKT9 Fab′ (Supplementary Fig. S14). Indeed, at 3.5 mg/kg siRNA equivalents, conjugation of Hprt siRNA to the OKT9 antibody fragment resulted in almost 90% target mRNA reduction in the liver, whereas conjugation to the Bicycle peptide resulted in only 43% hepatic knockdown of Hprt mRNA (Supplementary Fig. S14).

### Bicycle conjugation enhances ASO activity in myonuclei

**Figure 5.**
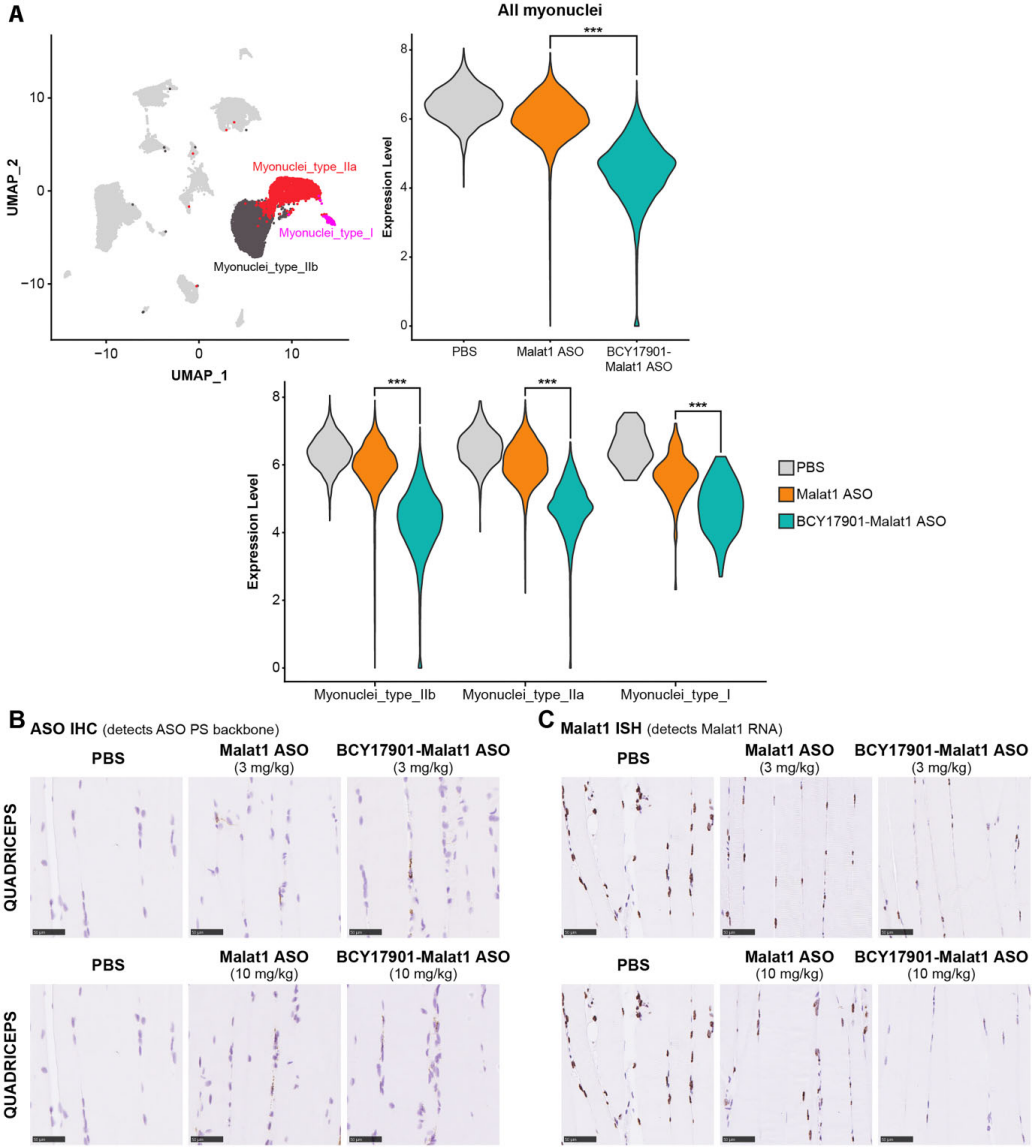
ASO conjugation to BCY17901 improves activity in myonuclei of all muscle fiber subtypes. (**A**) Top left panel: annotation of cell types identified in the snRNAseq analysis of gastrocnemius muscle plotted in a uniform manifold approximation and projection embedding. Myonuclei subtypes are annotated in distinct colors. Top right panel: violin plot of mouse Malat1 expression levels in myonuclei of gastrocnemius muscle from mice treated with PBS (vehicle, blue), unconjugated Malat1 ASO (ASO, red), and BCY17901-conjugated Malat1 ASO (green). Bottom panel: violin plot showing mouse Malat1 expression levels (normalized counts) in different myofiber subtypes (i.e. types IIb, IIa, and I). Data from *n* = 8 mice (2 mice dosed with PBS vehicle control, 3 mice with unconjugated Malat1 ASO, and 3 mice with BCY17901-conjugated Malat1 ASO). Asterisks (***) indicate adjusted *P*-value <.001 in differential expression test comparing unconjugated and BCY17901-conjugated Malat1 ASOs (using MAST with Bonferroni correction). (**B**) Representative images of ASO IHC (labels ASO in brown) in histological sections of quadriceps muscle from human TfR1 KI mice dosed with PBS (vehicle control), unconjugated Malat1 ASO, or BCY17901-conjugated Malat1 ASO. The top panels report the results for the 3 mg/kg ASO equivalent dose level, whereas the bottom panels report the results for the 10 mg/kg dose level. Scale bars: 50 μm. (**C**) Representative images of Malat1 ISH (labels Malat1 RNA in brown) in histological sections of quadriceps muscle. Scale bars: 50 μm. The PBS images in top and bottom panels of (B) are identical, as they were intentionally duplicated for ease of comparison versus both the 3 and 10 mg/kg treatment groups, respectively. The PBS images represent the same experimental conditions used across all treatment groups. Similarly, the PBS images in top and bottom panels of (C) are identical.

Single-nucleus RNA sequencing (snRNAseq) was performed to gain a detailed understanding of how human TfR1-targeting Bicycle conjugation improves ASO activity in the different cell types, and the different types of myofibers that make up the gastrocnemius muscle of human TfR1 KI mice (Fig. 5A and Supplementary Fig. S15A and B). Consistent with the bulk tissue RT-qPCR results obtained in gastrocnemius muscle, snRNAseq demonstrated that BCY17901–Malat1 ASO dosed at 3 mg/kg (ASO equivalents) lowers Malat1 RNA levels significantly more than the unconjugated ASO in myonuclei (Fig. 5A and Supplementary Fig. S15B). In addition, the snRNAseq analysis revealed that BCY17901 conjugation improved the Malat1 ASO activity similarly across the nuclei of the different myofiber types that compose the mixed gastrocnemius muscle, including the fast-twitch type IIb and IIa, and the slow-twitch type I myonuclei (Fig. 5A and Supplementary Fig. S15B). Therefore, the snRNAseq analysis confirmed the bulk tissue RT-qPCR data obtained in soleus (predominantly slow-twitch) and EDL (predominantly fast-twitch) muscle groups (Supplementary Fig. S11A). To a lesser, but still statistically significant extent, BCY17901 conjugation improved ASO activity also in nuclei of smooth muscle cells and adipocytes (Supplementary Fig. S15B).

In line with the snRNAseq data, analysis of ASO immunohistochemistry (IHC) in histological sections of quadriceps muscle of the human TfR1 KI mice further corroborated that the improvement in potency conferred by BCY17901 conjugation over unconjugated Malat1 ASO at the 3 and 10 mg/kg equivalent doses is associated with enhanced ASO localization at the sarcolemma (Fig. 5B, ASO IHC). Furthermore, *in situ* hybridization (ISH) to label Malat1 RNA in histological section of quadriceps muscle demonstrated that the BCY17901 conjugate at 3 and 10 mg/kg ASO equivalents achieved robust reduction of the target Malat1 RNA inside the myonuclei, whereas the unconjugated ASO did not (Fig. 5C, Malat1 ISH). Similarly, in the heart of the human TfR1 KI mice, BCY17901 conjugation enhanced ASO uptake (assessed by ASO IHC; Supplementary Fig. S16A) and activity (assessed by Malat1 RNA ISH; Supplementary Fig. S16B) inside the cardiomyocytes, compared to the unconjugated Malat1 ASO.

### Conjugation of ASO and siRNA to TfR1-targeting Bicycle peptide ligand results in good target knockdown in skeletal muscle and heart of NHPs

**Figure 6.**
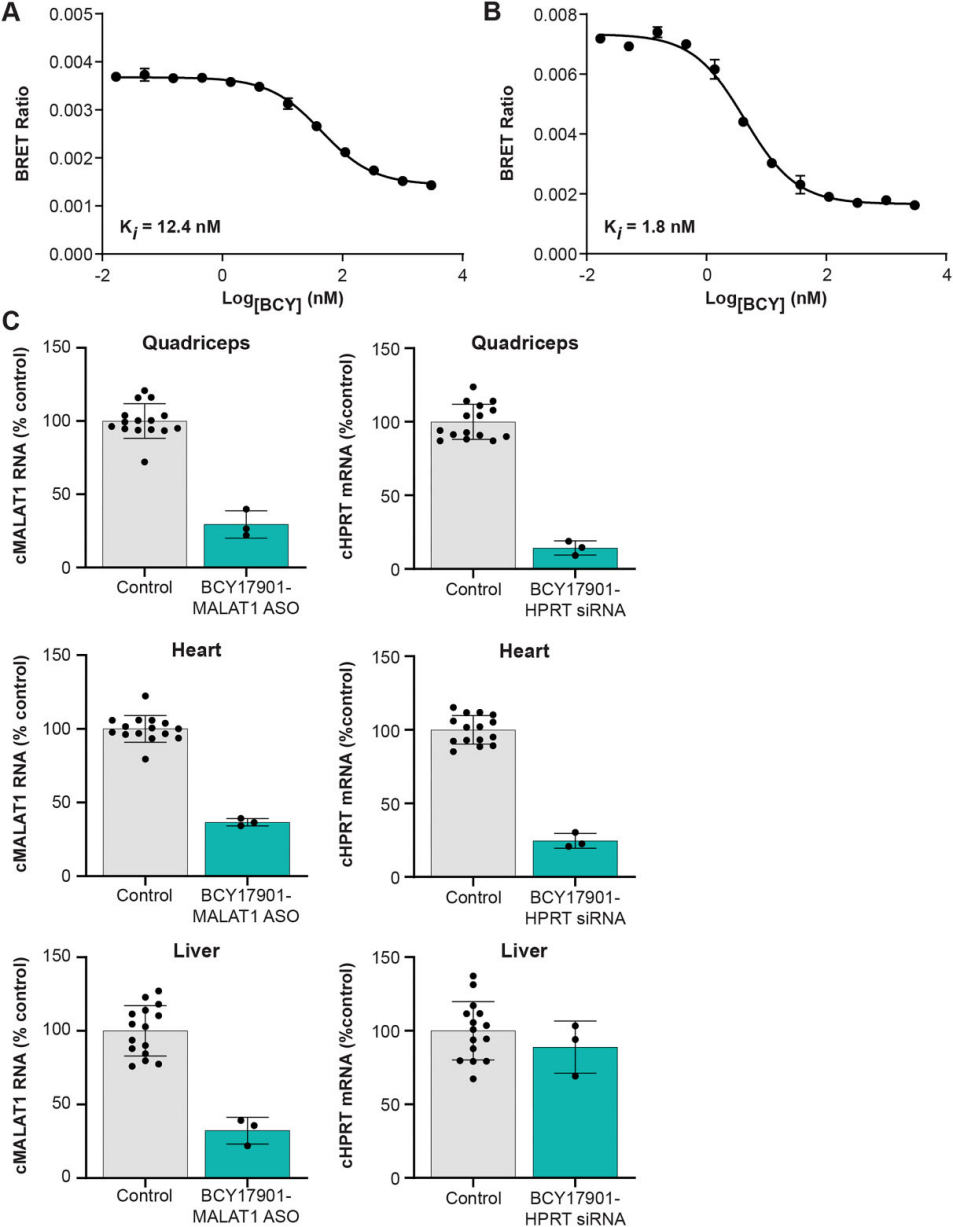
BCY17901-conjugated MALAT1 ASO and HPRT siRNA achieve robust target knockdown in skeletal muscle and heart of NHPs. Determination of Bicycle–ASO conjugate affinity to (**A**) cynomolgus monkey TfR1 and (**B**) human TfR1 using BRET. (**C**) Cynomolgus monkey MALAT1 (cMALAT1) RNA and HPRT (cHPRT1) mRNA levels measured by RT-qPCR in quadriceps muscle, heart, and liver of NHPs dosed by intravenous infusion with 25 mg/kg BCY17901-conjugated MALAT1 ASO and HPRT siRNA. The expression data were normalized using RiboGreen and are reported as percentage of control-treated animals (NHPs dosed with non-MALAT1 or non-HPRT targeting compounds). Error bars indicate standard deviation.

Importantly, Bicycle-conjugated ASOs and siRNAs could efficiently reduce their respective target RNAs in skeletal and cardiac muscles of NHPs (Fig. 6). Of note, BCY17901 has ∼10-fold lower affinity for the cynomolgus monkey TfR1 (Fig. 6A) compared to the human receptor (Fig. 6B), as measured by BRET. Despite the lower affinity of the ligand for the cognate NHP receptor, conjugation of MALAT1 ASO and HPRT siRNA to BCY17901 resulted in 71% and 86% target RNA knockdown, respectively, in quadriceps muscle (Fig. 6C). In the heart, BCY-MALAT1 ASO and BCY-HPRT siRNA reached 63% and 75% target reduction, respectively (Fig. 6C). Similar to the observations in mice, conjugation of HPRT siRNA to BCY17901 resulted in a partial liver-sparing effect, leading to only 11% target knockdown in hepatic tissue (Fig. 6C). *In situ* hybridization to detect MALAT1 RNA (Supplementary Fig. S17A), as well as immunohistochemistry to measure HPRT protein (Supplementary Fig. S17B), in histological sections of quadriceps muscle and heart from the NHPs corroborated the bulk tissue RT-qPCR data. Indeed, the histological stains are consistent with robust localization (ASO IHC, siRNA IHC) and activity (MALAT1 ISH, HPRT IHC) of the Bicycle–ON conjugates in skeletal and cardiac muscles (Supplementary Fig. S17A and B).

Due to the high expression of TfR1 in bone marrow [21], we used ASO IHC to assess the biodistribution pattern of BCY17901-conjugated Malat1 ASO in the bone marrow of NHPs (Supplementary Fig. S18A). Interestingly, BCY17901–Malat1 ASO uptake was seen in endothelial cells of sinusoidal capillaries and the stromal mature monocyte population, but not in progenitor cell of the erythroid or myeloid lineage (Supplementary Fig. S18A). Consistently, no significant Malat1 RNA knockdown was measured by RT-qPCR in the bone marrow of NHPs dosed with BCY17901–Malat1 ASO (Supplementary Fig. S18B).

### Subcutaneous administration of Bicycle–ASO conjugate results in robust target knockdown and improved potency over unconjugated ASO in skeletal muscle and heart of human TfR1 KI mice

**Figure 7.**
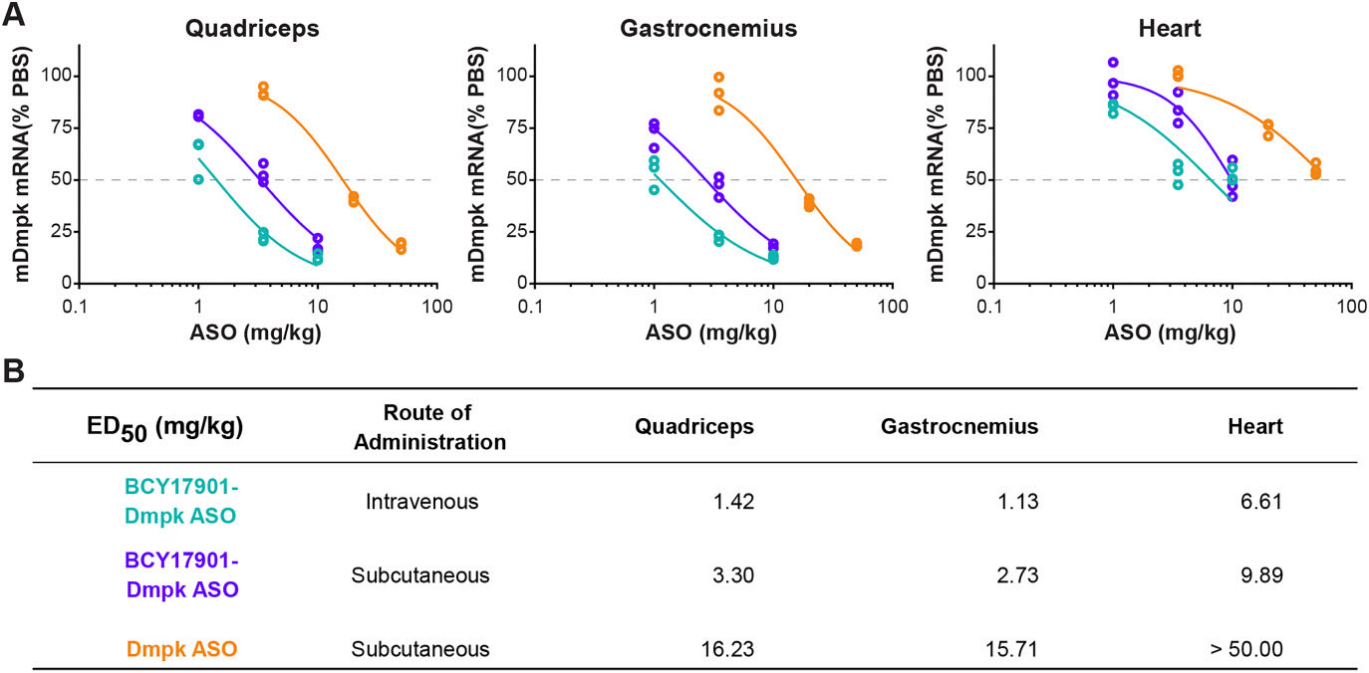
Subcutaneous administration of Bicycle–ASO conjugate results in robust target knockdown and improved potency compared to unconjugated ASO in skeletal muscle and heart of human TfR1 heterozygote KI mice. (**A**) Dose-dependent target knockdown of mouse Dmpk (mDmpk) mRNA was measured by RT-qPCR in various skeletal muscle groups (quadriceps and gastrocnemius) and heart of human TfR1 KI mice after both intravenous (light blue) and subcutaneous injection (purple) of Dmpk ASO conjugated to BCY17901. Unconjugated ASO (orange) dosed subcutaneously was included in the study to compare its potency versus the Bicycle-conjugated counterpart. Data are expressed as percentage target RNA level compared to PBS-treated (vehicle control) mice, after normalization using mouse Gapdh as housekeeping gene. Each open dot represents one animal. (**B**) ED_50_ values extrapolated from the graphs in panel (A) were calculated in GraphPad Prism software using the following constraints: top = 100, bottom = 0, Hill slope <−1. Doses refer to the ASO component of the LICA molecules (ASO equivalents).

The small size and favorable biophysical properties of the Bicycle peptides offer several advantages versus other LICA strategies based on antibody ligands, including the possibility to deliver the Bicycle–ON conjugates subcutaneously. Indeed, subcutaneous injection of BCY17901-conjugated Dmpk ASO resulted in robust, dose-responsive target mRNA knockdown in skeletal muscle and heart of human TfR1 KI mice (Fig. 7A and B). Compared to unconjugated Dmpk ASO, the Bicycle conjugate was 4.9-fold more potent in quadriceps muscle and 5.8-fold more potent in the gastrocnemius muscle, as reflected by their respective ED_50_ values (Fig. 7B). In the heart, the unconjugated ASO was less effective, whereas the BCY17901–Dmpk ASO conjugate reached 50% reduction of the target mRNA at much lower ASO equivalent doses (Fig. 7A and B).

## Discussion

ON-based drugs are uniquely suited to tackle several monogenic diseases affecting the skeletal muscle and heart, using either splicing modulation approaches or ASO/siRNA approaches that rely on RNase H1/Ago2-mediated degradation of target mRNAs. However, the delivery of ASOs and siRNAs to skeletal and cardiac muscles needs to be improved for broad therapeutic applicability. To be effective in muscle, ASOs and siRNAs must travel from the site of injection to their RNA target, traversing the capillary endothelium to leave the blood compartment and reach the tissue interstitium, and finally entering the cell. ASOs can more easily pass through the wider inter- and intracellular gaps in fenestrated and sinusoidal capillary endothelium in tissues such as kidney or liver, whereas the continuous endothelium in tissues such as skeletal muscle and heart limits the paracellular movement of systemically dosed ON drugs [22, 23]. Similarly, upon arrival at the target cell surface of interest, nucleic acid drugs must adhere to cell surface proteins, which can assist their cellular entry via endocytic processes. TfR1 is a highly recycling receptor expressed on the surface of various cell types, including myofibers and cardiomyocytes, as well as endothelial cells, where it undergoes constitutive endocytosis to transport transferrin-bound ferric ions into the cell [24]. The biological properties of the transferrin receptor make it an ideal candidate for LICA strategies, where conjugation of ASOs and siRNAs to ligands that bind TfR1 promotes their delivery to skeletal and cardiac muscle cells [7, 22].

Various types of anti-TfR1 ligands have been engineered for incorporation in LICA molecules. While antibodies provided the initial validation for the TfR1 LICA approach, they are associated with significant limitations, many of which are linked to their large molecular size. Selection of smaller human TfR1-targeting ligands with the optimal biophysical and biochemical properties is critical to maximize the potential of the muscle TfR1 LICA platform. We have explored a new class of ligands, the Bicycle molecules, which are chemically synthesized bicyclic peptides of ∼2 kDa in size. Bicycle molecules can be selected to bind the cognate human TfR1 protein with high specificity. The antibody-like specificity of binding, combined with the small drug-like scaffold, makes Bicycle peptides ideal ligands for LICA purposes. Especially, compared to monoclonal antibodies (mAb, ∼150 kDa) and antibody fragments (Fab, ∼50 kDa), Bicycle ligands (∼2 kDa) allow the reduction of the total dose of drug that needs to be administered in order to deliver the same number of ASO or siRNA molecules (i.e. ASO or siRNA equivalents) to skeletal and cardiac muscles, enhancing the overall LICA product profile. Indeed, achieving a hypothetical therapeutic dose of 3 mg/kg ASO equivalents would entail dosing a patient with a total drug mass of ∼6000 mg mAb–ASO conjugate, versus only ∼250–300 mg of Bicycle–ASO. Similar calculations apply to siRNA conjugates (Supplementary Fig. S19A and B). Other advantages that originate from the optimal biochemical and biophysical properties of the Bicycle ligands include the possibility of delivering the conjugates via subcutaneous injection.

Using a combination of phage display, structural biology, and medicinal chemistry, a high-affinity human TfR1-specific Bicycle binder was identified and optimized for conjugation to ASOs or siRNAs. A Retrogenix off-target screening cell microarray assay demonstrated exquisite specificity of binding of the lead Bicycle for human TfR1. In the same assay, a Bicycle–siRNA conjugate showed an additional significant interaction with RNase1, a protein known to bind both single- and double-stranded RNAs [25]. This secondary interaction is therefore not unexpected and is driven by the siRNA component of the LICA molecule, as demonstrated by the fact that the Bicycle peptide alone does not interact with RNase1.

To prove the potential of LICA approaches based on TfR1-targeting Bicycles for human applications, we have generated a humanized mouse model that expresses human TfR1. In the human TfR1 KI mice, we screened Bicycle molecules that have different binding affinities for the human TfR1 receptor, after conjugation to well-characterized tool ASOs and siRNAs targeting either Dmpk, which is a muscle-expressed gene, or Malat1, which is a ubiquitously and abundantly expressed lncRNA. Our *in vivo* screening efforts confirmed the requirement for high-affinity binding of the ligand for the cognate receptor to achieve robust activity of ON conjugates in skeletal and cardiac muscles of human TfR1-expressing mice.

Bicycle conjugation improved ASO and siRNA activity with similar efficiency in different muscle fiber types in mice and NHPs. Histological analysis of tissue samples suggests that the improvement in potency in skeletal muscle conferred by TfR1 Bicycle conjugation is associated with enhanced ASO localization at the sarcolemma and enhanced ASO uptake in the myofibers. snRNAseq analysis performed in skeletal muscle tissue isolated from mice treated with BCY17901–Malat1 ASO corroborated the conclusion that Bicycle conjugation improves ASO activity in myonuclei of type IIb, IIa, and I myofibers. Importantly, we demonstrated that binding of BCY17901 to human TfR1 does not reduce the amount of receptor protein present on the surface of skeletal and cardiac muscle cells, suggesting no interference of this high-affinity ligand with the trafficking of TfR1 back to the cell surface (Supplementary Fig. S12).

Leveraging the high TfR1 expression on the endothelial cells of the BBB, TfR1-targeting strategies have been used in recent years to transport ASOs across the BBB via transferrin receptor-mediated transcytosis [18, 26–29]. However, we did not observe significant activity in central nervous system tissues after systemic administration of BCY17901–ASO in human TfR KI mice, suggesting that a simple Bicycle–ON conjugate cannot cross the BBB. It might be that different properties are required to effectively engage the receptor-mediated transcytosis mechanism in the BBB, such as lower binding affinity for TfR1 [18]. Besides the endothelial cells of the BBB, the bone marrow is also a tissue where TfR1 is highly expressed [21]. Interestingly, BCY17901–Malat1 ASO uptake was seen in endothelial cells of sinusoidal capillaries and the stromal mature monocyte population, but not in progenitor cells of the erythroid or myeloid lineage (Supplementary Fig. S18A).

Our work presents with some limitations and still unresolved questions. In particular, given that TfR1 is expressed in both endothelial cells and myofibers, our Bicycle LICA strategy conceptually offers the opportunity to enhance both the passage of ON across the endothelial layer via transcytosis and the direct ON uptake in muscle cells. More work is needed to dissect the contribution of each to the activity of the LICA molecule. Furthering the understanding of when the ON cargo dissociates from the Bicycle component of the LICA molecule in the different cell types (e.g. endothelial cells, myofibers, and cardiomyocytes) and in different intracellular compartments will also be instrumental.

Although prior work validated the TfR1-targeting strategy for the delivery of ASOs and siRNAs to muscle in rodents [7, 8, 30], the translatability of the TfR1 LICA approach to human remains an important question to be addressed. Notably, the expression levels of human TfR1 in skeletal muscle and heart of our hTfR1 KI mouse are comparable to normal human tissues (Supplementary Fig. S8C). However, a point of caution when examining the activity of the BCY17901–Dmpk ASO conjugate in the mouse cardiac tissue is represented by the fact that the murine heart, differently from the human heart, might contain cell populations that express Dmpk but not TfR1 (https://tabula-muris.ds.czbiohub.org/; https://tabula-sapiens-portal.ds.czbiohub.org/), therefore potentially reducing the efficacy of a TfR1 LICA approach in the mouse cardiac tissue. Other modalities based on TfR1 targeting to deliver ON to muscle have generated encouraging results in humans, providing a favorable outlook in terms of translatability of the TfR1 LICA platform [8, 23, 26, 31, 32].

In conclusion, we have demonstrated that conjugation to Bicycle peptide ligands that target human TfR1 enhances ASO and siRNA activity in skeletal and cardiac muscles of human TfR1-expressing mice and NHPs. Our targeted delivery approach based on the combination of antisense and Bicycle technologies opens the door to the design of medicines that have antibody-like selectivity with a low total molecular weight, ultimately enabling the potential treatment of devastating diseases that affect the skeletal muscle and heart.

## Supplementary Material

gkaf270_Supplemental_File

## Data Availability

All data are available upon request. The model coordinates and structure factors have been deposited in the Protein Data Bank under accession number 9GH7.
