# Supplementary material for: Conjugation to a transferrin receptor 1-binding Bicycle peptide enhances ASO and siRNA potency in skeletal and cardiac muscles
Source: Nucleic Acids Res. 2025 Apr 10;53(7):gkaf270. doi: 10.1093/nar/gkaf270 (PMC11983102; doi:10.1093/nar/gkaf270)
Supplement: gkaf270_Supplemental_File [file gkaf270_supplemental_file.docx]

**SUPPLEMENTARY DATA**

**Conjugation to a transferrin receptor 1-binding Bicycle peptide enhances ASO and siRNA potency in skeletal and cardiac muscle**

Michael E. Østergaard^1^†*, Michele Carrer^1^†*, Brooke A. Anderson^1^, Megan Afetian^1^, Mohsen A. Bakooshli^1^, Jinro A. Santos^1^, Stephanie K. Klein^1^, Juliana Capitanio^1^, Graeme C. Freestone^1^, Michael Tanowitz^1^, Rodrigo Galindo-Murillo^1^, Hans J. Gaus^1^, Chrissa A. Dwyer^1^, Michaela Jackson^1^, Paymaan Jafar-nejad^1^, Frank Rigo^1^, Punit P. Seth^1^, Katherine U. Gaynor^2^, Steven J. Stanway^2^, Liudvikas Urbonas^2^, Megan A. St. Denis^2^, Simone Pellegrino^2^, Gustavo A. Bezerra^2^, Michael Rigby^2^, Ellen Gowans^2^, Katerine Van Rietschoten^2^, Paul Beswick^2^, Liuhong Chen^2^, Michael J. Skynner^2^ and Eric E. Swayze^1^

SUPPLEMENTARY MATERIALS AND METHODS

Human TfR1 protein cloning and expression

The gene encoding human TfR1 (aa C89-F760) with an N-terminal poly-histidine (His_6_) tag and a prolactin leader sequence was cloned into a pcDNA3.1 vector for transient mammalian expression. 900 mL culture of Expi293 freestyle GnTi- cells were grown in Expi293F expression medium (Life Technologies) at 37 °C, 70% humidity, 8% CO_2_, and rotating at 150 rpm. Cells were transfected using PEI-MAX (Polyscience) and cultivated for 4 days before harvesting. Cells were spun down at 800 x g for 15 minutes and supernatant was recovered and filtered before proceeding to the next step.

Human His_6_-TfR1 purification

Soluble human His_6_-TfR1 was purified from the harvested mammalian cell media using nickel sepharose excel resin (Cytiva product code 17371201), previously equilibrated in buffer A (20 mM Tris-HCl pH 8, 150 mM NaCl, 1 mM TCEP, 5% glycerol and 10 mM imidazole). Once the sample was loaded, the column was washed to baseline using buffer A and, afterwards, the protein was eluted in a single step using buffer B (20 mM Tris-HCl pH 8, 150 mM NaCl, 1 mM TCEP, 5% glycerol and 500 mM imidazole). The protein was further purified using size exclusion chromatography (SEC) S200 HiLoad 16/60 column (GE Healthcare Superdex) in a final storage buffer containing 20 mM Tris-HCl pH 8, 150 mM NaCl, 1 mM TCEP and 5% glycerol. Human His_6_-TfR1 protein was eluted from the SEC column at > 95% purity, as determined by SDS-PAGE, and concentrated to 10 mg/mL for crystallization using 10 kDa MWCO centrifugal concentrators (Millipore).

Identification of human TfR1 Bicycle binders by phage display

Identification of the lead human TfR1 Bicycle binder took place via iterative phage selections. Bacteriophage (phage) presenting linear peptides with up to 12 randomized positions and 3 cysteines, were cyclized in situ to form thioether-bonded bicyclic peptide libraries. These were used in panning experiments (selections) against N-terminally biotinylated human TfR1 (ACROBiosystems CD1-H5243). Multiple rounds of selection were performed using decreasing target concentrations of protein immobilized onto streptavidin magnetic beads. Eluted phage clones were isolated, sequenced, and tested for binding to proteins using an *AlphaScreen* assay (BMG Labtech). Binders were chemically synthesized as peptides for further characterization. Selections were then repeated, using initial peptides as tool peptides for blocking selections, to bias binding of the phage towards alternative epitopes. Certain lead clones subsequently returned to the phage platform for further affinity maturation. For these, custom *Bicycle* phage libraries were constructed based on the initial sequence, retaining some residues and randomizing others. Phage selections using these custom libraries were carried out as before to identify binders with improved affinity over the parent sequences.

Cell Internalization and co-localization experiments

HT1080 cells were purchased from ATCC (Cat: CCL-121). Minimum essential media (MEM) was purchased from Thermo Fisher Scientific (Cat: 11095-080), with supplements penicillin-streptomycin and fetal bovine serum (FBS) purchased from Sigma-Aldrich (Cat: P4333 and F7524, respectively). Paraformaldehyde (PFA), human transferrin Alexa Fluor 488, human transferrin Alexa Fluor 647, Hoechst, goat serum, mouse anti-human TfR1, goat anti-mouse Alexa Fluor 546, goat anti-mouse Alexa Fluor 594, goat anti-rabbit Alexa Fluor 546 and goat anti-rabbit Alexa Fluor 647 were all purchased from Thermo Fisher Scientific (Cat: 28908, T13342, T23366, 62249, 16210064, A11130, A11030, A11032, A11035 and A21244, respectively). Rabbit anti-human EEA1 and DYNGO-4A were purchased from Abcam (Cat: ab109110 and ab120689, respectively), with rabbit anti-human RAB5, mouse anti-human LAMP1, mouse anti-human RAB7 and rabbit anti-human RAB11 all purchased from Cell Signaling Technology (Cat: 3547, 15665, 95746 and 5589, respectively). Acetone was purchased from Fisher Scientific (Cat: A/0560/PC21) with Triton X-100 (TX-100) and PBS was purchased from Sigma-Aldrich (Cat: X-100 and D8537, respectively). Black-sided, clear-bottom 96-well imaging plates were purchased from Greiner Bio-One (Cat: 655090).

HT1080 cells were cultured in MEM media supplemented with 10% FBS and 1% penicillin-streptomycin. Cells were seeded at 20,000 cells/well into 96-well imaging plates and incubated overnight at 37°C, 5% CO_2_ before imaging or endocytosis experiments.

Cells were fixed and permeabilized by washing them twice with PBS and incubating them with 80% acetone/PBS for 10 minutes at -20°C. After washing them once with PBS, the cells were blocked with 10% goat serum/PBS (blocking buffer) for 1 hour at room temperature. The cells were then incubated with primary antibody, prepared in blocking buffer at the below dilutions for 1 hour at room temperature. For any conditions with no primary antibody (secondary alone controls, transferrin/Bicycle peptide alone) cells were incubated with blocking buffer. Supplementary Table 2 lists the primary antibodies and respective dilutions used for immunostaining. After washing them twice with PBS, the cells were incubated with secondary antibody; goat anti-mouse Alexa Fluor 546/594 and/or goat anti-rabbit Alexa Fluor 546/647 (each at 1 in 500) plus Hoechst (at 1 in 1,000), prepared in blocking buffer for 1 hour at room temperature. Cells were then washed twice in PBS and left in PBS for imaging.

For transferrin and Bicycle peptide endocytosis experiments, cells were washed twice with serum-free media and incubated with serum-free media for 90 minutes at 37°C. Cells were then incubated with human transferrin (conjugated to either Alexa Fluor 488 or 647; 25 µg/ml) and/or Bicycle (BCY20351, Oregon Green 488-conjugated; 1.0 µM), both prepared in serum-free media, for 1 hour at 4°C. Cells were then moved to 37°C for various amounts of time to allow endocytosis to occur. Cells were then washed with PBS, fixed/permeabilized, and immunostained as described above. For experiments using DYNGO-4A, cells were treated with either vehicle (0.1% DMSO) or DYNGO-4A (30 µM), prepared in serum-free media, for 30 minutes at 37°C before incubation with conjugated transferrin. Cells were visualized using an ImageXpress (Molecular Devices) confocal microscope at 40x magnification and images were exported using MetaXpress version 6.7 (Molecular Devices). Representative images are from triplicate culture wells. Data is from one experiment.

Crystallization, data collection and structure solution of the human His_6_-TfR1 in complex with BCY15466

All work relative to protein production, crystallization and structure solution was performed at Charles River Laboratories (CRL, Cambridge, UK). Human His_6_-TfR1 (at final concentration of 10 mg/mL was incubated with a 2x molar excess of BCY15466 (sequence ACPPDAHLGCISWCA[CONH2]) in ice for 60 minutes. Crystals were grown by the hanging drop method: drops of 1:0.5 μL protein:reservoir ratio were set up in 24-well plates using several commercial screens. Crystals of the human His_6_-TfR1:BCY15466 complex were grown in condition G5 from the PEG/Ion HT screen (Hampton Research), in hanging drops containing 0.1 M ammonium tartrate dibasic, pH 7.0 and 12% (w/v) PEG 3350. Crystals were harvested and cryoprotected in reservoir solution enriched with 20% glycerol before cryocooling in liquid nitrogen. Complete datasets were collected by Unattended Data Collection (UDC) at beamline i03 of Diamond Light Source (DLS). Data was initially auto-processed using autoPROC (1) (Supplementary Table 3) at a final resolution of 2.3 Å. The structure was solved by molecular replacement using a model previously generated by CRL as a reference within Phaser (2). The calculated electron density map presented a continuous portion unaccounted by human His_6_-TfR1 and consistent with the sequence of BCY15466 (ACPPDAHLGCISWCA[CONH2]). The initial model was used for iterative cycles of manual model building and refinement using Coot (3) and Refmac5 (4), respectively. Final refinement statistics are shown in Supplementary Table 3.

Figures were generated using ChimeraX v1.6 (5) and Pymol (The PyMOL Molecular Graphics System, Version 3.0 Schrödinger, LLC).

Determination of Bicycle peptide binding to human and cynomolgus monkey TfR1 using surface plasmon resonance (SPR)

Biacore experiments were performed to determine k_a_ (M^-1^s^-1^), k_d_ (s^-1^), K_D_ (nM) values of various peptides binding to TfR1. Recombinant human and cynomolgus monkey TfR1 were received from Bicycle Therapeutics as His_6_-tagged TfR1 (a.a. 89-760) (ACROBiosystems, CD1-H5243 and TfR-C524a). SPR binding analysis was performed on Biacore T200 and Biacore 8K+ (Cytiva). Human TfR1 and randomly biotinylated cynomolgus monkey TfR1 were captured on Series S Sensor Chip SA (BR100531, Cytiva) at levels of approximately 1000 RU. Using an appropriate top concentration, Bicycle peptides were screened in a 5-point titration curve in single cycle kinetics format at 30 ml/minute. The running buffer was PBS-P^+^, 1% DMSO at pH 7.4. The data was solvent-corrected for DMSO bulk effects. In addition, all data was double corrected against the reference flow cell and matched buffer blanks. Data processing and kinetic fitting were performed using Biacore T200 Evaluation Software or Biacore Insight Evaluation. The data was fitted using the 1:1 binding model or steady-state affinity model where appropriate. The geometric mean (geomean) K_D_ (nM) for binders was calculated by transforming unique K_D_ values by log10, arithmetic-mean-averaging the transformed values, and subsequently anti-log-transforming the averaged value.

Determination of the affinity of the ASO-Bicycle conjugate to human TfR1 using Bioluminescence Resonance Energy Transfer (BRET)

A human TfR1-Nluc fusion protein was constructed by linking NanoLuc (ProMega) through its N-terminal Val to the C-terminal F760 residue of human TfR1, using a GGGSGGSSG flexible linker. A fluorescently labeled (Alexa Fluor 594) Dmpk ASO was conjugated to the Bicycle ligand BCY17871 through a strain promoted azide-alkyne cycloaddition (SPAAC) reaction (called AF594-BCY17871-ASO). Crude membrane fractions from HEK293 cells stably expressing human TfR1-Nluc were resuspended in PBS and 100 µL of the suspension were dispensed into white 96-well assay plates (Thermo Fisher Scientific, Cat: 136101), an amount corresponding to 10,000 cells per well. Membranes were treated with 11.1 µL of AF594-BCY17871-ASO at serially-diluted final concentrations of 1000-0.006 nM. The mixtures were incubated for 3 hours at room temperature to reach equilibrium. To initiate BRET, 12.4 µL of 100 µM of the Nluc substrate furimazine were added to each well, and the mixtures were incubated for 5-30 minutes. The assay plate was then read on a Promega GlowMax Discover plate reader at wavelengths of 450 nm and 600 nm, and the ratio of emissions at wavelengths 450/600 was used to yield %BRET efficiency. Data was subjected to non-linear regression, then fitted to a single site binding hyperbolic function. The dissociation constant k_d_ of AF594-BCY17871-ASO was determined. In order to evaluate modified oligonucleotides conjugated to human TfR1 Bicycle ligands, the assay was modified as follows: 100 µL of crude membrane fractions from stably transfected human TfR1-Nluc HEK293 cells were dispensed into white 96-well assay plates. AF594-BCY17871-ASO was used as a tracer compound and added to each well at a final concentration approximately equivalent to the tracer k_d_. Modified oligonucleotides were conjugated to the Bicycle peptides through a SPAAC reaction. These were then added at a range of concentrations in triplicate assays points, and the mixtures were incubated for 3 hours at room temperature. BRET was initiated with the addition of furimazine and the assay was completed as described above. Inhibition constants (K_i_) were obtained by fitting %BRET efficiency values to a competitive inhibition model, using the k_d_ value estimated for AF594-BCY17871-ASO obtained in the same run. Values are presented as the average of triplicate data.

Determination of Bicycle peptide binding to TfR1 using fluorescence polarization

Competition fluorescence polarization assays were run by observing the fluorescence polarization of a fluorescent “tracer” Bicycle which competes against an unlabeled peptide for binding to the human TfR1 target, as previously described for EphA2 (6). 10 µL of protein was mixed with either 5 µL of peptide or 5 µL of buffer and then the assay was initiated by addition of 10 µL of tracer (1 nM) (final volume 25 µL), in a black 384 low bind, low volume plate (Corning). The buffer was 25 mM HEPES (Sigma), 2% (v/v) glycerol (Sigma), 100 mM NaCl (Sigma), 0.01% Tween-20 (Thermo Fisher Scientific, Cat: 85113) adjusted to pH 7.4. The plate was incubated for 1 hour at 25°C and then read in a PHERAstar FS (BMG) using an ‘FP 485 520 520’ optic module (excitation 485 nm, emission 520 nm). The gain was determined immediately prior to the read on a tracer-only well.

The k_d_ of the tracer molecule was found using the above method with the exclusion of the competing peptide and titration of the protein concentration (10 nM) against a fixed concentration of the tracer. The EC_50_ of the interaction was found using a 4-point logistic model:

$$fluorescence polarization=unbound+\frac{{\left( max-unbound \right)\times[hTfR1]}^{slope}}{{[hTfR1]}^{slope}+{EC50}^{slope}}$$

Where *unbound* is the fluorescence polarization value corresponding to the unbound tracer, *max* is the fluorescence polarization value corresponding to maximally bound tracer, and *slope* is the Hill slope used. In a competition fluorescence polarization assay typically 12 concentrations of unlabeled Bicycle in a 2-fold titration from 5 µM to 2 nM were used to generate a dose-response curve against a fixed concentration of tracer and human TfR1. The Cheng-Prusoff equation was then applied to find the *K_i_* using a value for the tracer k_d_ of 2 nM:

$$K_{i}=\frac{{IC}_{50}}{1+([tracer]/K_{d}}$$

Where *IC_50_* is the inflection point of the dose-response curve.

Custom Dotmatics (Dotmatics) workflows were used to process the data. Values quoted are the geometric means of at least 3 repeats.

## Medicinal chemistry optimization of lead Bicycle binder

Chemical optimization of the naïve hit BCY15468 was performed to further improve binding affinity, and identify Bicycle peptides with superior in vitro stability properties, suitable for progression to in vivo testing. An alanine scan was performed to identify core residues involved in binding to the protein and solvent-facing residues which could be used to tune molecular properties, followed by substitutions with non-natural amino acids to identify suitable replacements at each position. In addition, peptides were synthesized to investigate the removal of the N- and C-terminal alanines, capping the N-terminal with an acetyl group, and to examine the effects of combinations with N- and C-terminal extensions. Affinity was measured using either SPR or fluorescence polarization (FP) assays, and plasma stability in mouse was determined for selected examples. Series of analogues with the azidolysine (K(N_3_)) or azidopropanoic acid (AzPro) were prepared on the C- and N-terminal of the Bicycle molecules, respectively. These were used to conjugate to an ASO or siRNA for further profiling.

## In vitro selectivity assessment of Bicycle molecules (Retrogenix screen)

The Retrogenix Cell Microarray Technology platform was used to assess the binding specificity of both the lead Bicycle molecule alone (BCY peptide attached to biotin) and a biotinylated BCY-siRNA conjugate. In this assay, the two molecules were screened against human plasma membrane proteins and cell surface-tethered secreted proteins (including various transporters, adhesion molecules, enzymes, and receptors) to screen for potential off-target binding interactions. The selectivity of each test article (1 mM) was evaluated using fixed HEK293 cells expressing 6105 full-length human plasma membrane proteins, secreted, and cell surface-tethered human secreted proteins, plus a further 400 human heterodimers. Each library interaction was re-expressed, along with 2 control receptors, and re-tested with the respective test article or control treatments. This was performed on both fixed and live cells. Finally, a flow cytometry-based follow-on study was undertaken to investigate the identified test article-specific interactions further on live cells.

## Synthesis of OKT9 Fab’-ASO conjugate

OKT9 antibody (Bioxcell) was dissolved in 0.2 M sodium acetate buffer pH 4.0 at 1 mg/mL and pepsin (Sigma-Aldrich) was added in the same buffer (to give final pepsin concentration of 0.1 mg/mL). The mixture was incubated for 3 hours at 37^o^C, then cooled to room temperature and quenched by increasing pH to neutral with 2 M Tris base. The F(ab)_2_ generated was then purified by size exclusion chromatography (SEC) using a HiPrep 26/60 Saphacryl S200 HR column and 1x DPBS as the eluent. F(ab)_2_ (10 mg/mL in 1x DPBS) was then fully reduced using 20 mM TCEP (same volume 1x DPBS) followed by removal of TCEP using a 10 kDa dialysis cassette inserted in 1x DPBS at 4°C for 4 hours. The reduced antibody was then reacted with a 5’-maleimide-conjugated ASO (5 equivalents) for 2 hours at room temperature, followed by the addition of 8 mM dehydroascorbic acid in 1x DPBS (equal volume to reaction), and the mixture was left at 4°C overnight. The crude was purified by SEC as detailed above and concentrated using a 10 kDa centrifugal filter device.

Analysis of gene expression by quantitative real-time PCR (RT-qPCR)

The mRNA levels of target genes were measured by RT-qPCR in each sample using the Express One-Step RT-qPCR kit (Thermo Fisher Scientific, Cat: 11781200) and the standard 2 hour cycling program as outlined in the manufacturer’s protocol. Each target was measured using a forward and reverse primer plus a fluorescently labeled (5’-FAM) probe and an internal ZEN / 3’-Iowa Black^TM^ FQ double-quencher system. Primers and probes were from Integrated DNA Technologies (IDT) except for one TaqMan™ Gene Expression Assay (Thermo Fisher Scientific, Cat: Rh02800695_m1; 5’-FAM reporter and 3’-MGB quencher), which was used for the quantification of NHP HPRT according to the manufacturer’s protocol. Information for each primer-probe set, including gene, species reactivity, mismatches, and sequences can be found in the Supplementary Table 1. The final concentration of primers and probe in the RT-qPCR reaction was 0.4 μM and 0.1 μM, respectively. The TaqMan™ assay was used at 1X (0.9 μM and 0.25 μM for primers and probe, respectively). ~10 ng of total RNA input was used in each RT-qPCR reaction. To quantify the amount of mRNA expression, a 5-point standard curve was prepared for each tissue by preparing 2.5-fold serial dilutions of pooled total RNA extracted from control animal tissue, typically starting with a top standard amount of 12.5 ng input RNA. Each primer-probe set was run in triplicates, and the respective standard curves were included on each technical run (e.g., in the same reaction plate), along with a no-reverse transcription control and a no-RNA template control. The StepOnePlus Real-time PCR System (Thermo Fisher Scientific, Cat # 4376600) was used for cycling and amplification. The following cycling conditions were used: 1) 50°C for 15 minutes, 2) 95°C for 2 minutes, and 3) 45 cycles of 95°C for 15 seconds and 60°C for 1 minute. Raw data was exported and analyzed in Thermo Fisher Scientifics’s StepOne Software version 2.3. Target threshold was adjusted to 0.1, where the standard curve is in its linear phase, and the quality of the standard curves was confirmed to fit recommended guidelines of slope value between -3.0 and -3.6, with an R^2^ value of 0.99 or higher. The no-reverse transcription and no-RNA template control were confirmed to have no amplification, i.e., a Ct (cycle threshold) value of “undetermined” or 10 Cts more than control samples. Ct values for control samples were typically between 16 and 25. The target gene Ct values were normalized to the housekeeping reference gene Gapdh for mouse samples. The relative expression of target mRNAs was calculated using the respective standard curves to extrapolate from each reaction sample the mRNA quantities, which were then expressed as percentage relative to the vehicle control group. For NHP samples and Supplementary Figure 8C, the expression levels of target genes were normalized to total input RNA quantity as determined by RiboGreen RNA Assay Kit (Thermo Fisher Scientific, Cat: R11490), and data was represented as percentage relative to control groups.

## snRNA-seq sample preparation, sequencing, and analysis

Gastrocnemius muscle samples from a total of eight mice were processed for single nuclei RNA sequencing. Two mice had been dosed with PBS (vehicle control), three with unconjugated Malat1 ASO at 3 mg/kg, and three with BCY17901-Malat1 ASO at 3 mg/kg (ASO equivalents). Muscle nuclei were isolated using a modified version of the 10x Genomics Chromium Nuclei Isolation Kit for Single Cell Multiome ATAC + Gene Expression. In brief, approximately 50 mg of flash frozen gastrocnemius muscle were pulverized in Covaris tissueTUBE TT2 with one pulse of the CP02 cryoPREP Automated Dry pulverizer at the highest impact setting. Pulverized samples were transferred to pre-chilled sample dissociation tubes provided in the 10x Genomics Nuclei Isolation Kit, and nuclei were isolated as directed in the manufacturer’s instructions. Nuclei suspensions were diluted to 1,000 nuclei/μl using fluorescent nucleic acid staining (AOPI), and nuclei quality was confirmed under 40x magnification.

The single nuclei suspension was loaded onto Chromium X (10x Genomics) for targeting of 6,000 nuclei, according to the manufacturer's instructions. Libraries were constructed using Chromium Next GEM Single Cell 3' Kit v3.1 (10x Genomics). Quantity and quality of cDNA and indexed libraries were determined with Qubit 1X dsDNA HS Assay Kit (Thermo Fisher Scientific) and High Sensitivity DNA ScreenTape Analysis (Agilent). Pooled libraries were sequenced on NovaSeq 6000 SP platform (Illumina).

Fastq files were processed with 10x Genomics Cell Ranger 7.2.0 to align reads and generate feature-barcode matrices. 33,793 cells were sequenced. Data QC was performed using the Seurat (v4) package (7), and it involved ambient RNA removal (8) and filtering of low-quality cells, which were identified by mitochondrial RNA above 5%, number of detected gene above 3,000 or below 350, unique molecular identifier (UMI) counts above 10,000 or below 500, or complexity below 0.8. 5,233 cells failed QC and were removed, leaving 28,560 cells used for further analysis. Non-expressed genes and genes expressed by less than 10 cells were removed from the analysis. Data normalization and variance stabilization were performed using SCTransform (9), and data integration was performed using Harmony (10). After dimensionality reduction (UMAP, resolutions between 0.2 and 1.2) and clustering (shared nearest neighbor modularity optimization), cell types present in each cluster were identified based on the expression of curated marker genes. Data visualization was facilitated by the package scCustomize (11).

Histological analysis

Tissue samples of heart and quadriceps from mice and NHPs were fixated in 10% formalin for 48 to 72 hours. After embedding, 4 μm-thick sections were cut and placed on glass slides. All stains were performed on Ventana Discovery Ultra slide stainer. For Malat1 ISH on heart and quadriceps from mice and NHP, the following probes were used: RNAscope™ 2.5 VS Probe- Mus musculus Mm-Malat1-pooled O1 (ACD, Cat: 507729), and Macaca fascicularis Mf-Malat1-O1 (ACD, Cat: 490419). RNAScope 2.5 VS Universal Sample Prep Reagents v2 (ACD, Cat: 323740) was used for deparaffinization and target retrieval. RNAScope 2.5 VS Universal Detection Reagents (ACD, Cat: 323210) and the Discovery mRNA DAB Detection kit (Roche, Cat: 760-224) were used for amplification and detection. RNAscope™ 2.5 VS negative control probe dapB (ACD, Cat: 312039), and positive control probe Mm-PPIB (ACD, Cat: 313919) were used. DapB and PPIB probes were used for quality control. IHC to detect ASO in heart and quadriceps from mice and NHPs was performed using rabbit polyclonal ASO antibody (Ionis). Tissues were treated enzymatically with trypsin (Sigma, Cat: T8003). The primary antibody was diluted with Discovery Antibody Diluent (Ventana, Cat: 760-108) and incubated for 1 hour at 37°C. The slides were then blocked with Endogenous Biotin Blocking Kit (Ventana, Cat: 760-050) and normal goat serum (Jackson Immuno Labs, Cat: 005-000-121). The secondary antibody was biotin labeled goat anti-rabbit (Jackson Immuno Labs, Cat: 111-005-003). The secondary antibody was visualized with DABMap Kit (Ventana, Cat: 760-124). siRNA detection was achieved by enzymatic antigen retrieval of tissues with protease 2 for 8 minutes (Roche, Cat: 05266696001), followed by rabbit polyclonal siRNA antibody (Ionis) for 1 hour and secondary antibody OmniMap-anti rabbit HRP (Roche, Cat: 05269679001). For detection of HPRT1 protein, a primary antibody against HPRT1 (Abcam, Cat: ab109021) was used with antigen retrieval CC1 for 64 minutes (Roche, Cat: 05424569001). The antibody was incubated for 12 hours. As secondary antibody, anti-rabbit HQ (Roche, Cat: 760-4815) was used with amplification system anti-HQ HRP Multimer (Roche, Cat: 760-4602) and amplification HQ kit (Roche, Cat: 760-052). The secondary antibodies for siRNA and HPRT IHC were labeled with a chromoMap Kit (Roche, Cat: 05266645001). For TfR protein labelling, slides were stained with rabbit polyclonal anti-transferrin receptor antibody (Abcam, Cat: ab193188) on a Ventana Ultra staining system. Slides were treated with Heat Induced Antigen Retrieval (HIER) with Ventana CC1 solution (Ventana, Cat: 950-500) for 64 minutes. The slides were then blocked with Endogenous Biotin Blocking Kit (Ventana, Cat: 760-050) and normal goat serum (Jackson Immuno Labs, Cat: 005-000-121). The primary antibody was diluted with Discovery Antibody Diluent (Ventana, Cat: 760-108) and incubated for 1 hour at 37°C. The antibodies were detected with biotin labeled goat anti-rabbit secondary antibody (Jackson Immuno Labs, Cat: 111-005-003). The secondary antibody was visualized with DABMap Kit (Ventana, Cat: 760-124).

All images were scanned on a Hamamatsu S360 scanner at 20x resolution. Stained sections were analyzed and semi-quantitatively scored by a board-certified pathologist using light microscopy.

Western blot analysis

Whole frozen tibialis anterior muscle tissues were homogenized in RIPA buffer (ThermoFisher Scientific, Cat.: 89900) supplemented with protease and phosphatase inhibitors cocktail (ThermoFisher Scientific, Cat.: 78440). The tissue homogenate was then incubated on ice for 30 minutes after which it was centrifuged at 10,000 g at 4°C for 30 minutes, and the supernatant was collected for analysis. The sample protein concentration was measured using the Pierce^TM^ BCA protein assay kit (Thermo Fisher Scientific, Cat.: 23227). 20 μg of total protein lysate was resolved by electrophoresis on Bis-Tris 4-12% NuPAGE polyacrylamide gels using the SureLock™ Tandem Midi Gel Tank (Thermo Fisher Scientific, Cat: STM1001). The proteins were then transferred to a nitrocellulose membrane using a Trans-blot Turbo^TM^ transfer system (BioRad, cat.: 1704150) and the membrane was later blocked with 5% nonfat blotting grade milk solution (Biorad, Cat.: 1706404XTU) in Tris-buffered saline containing 0.1% Tween 20. The primary antibodies used in our studies were: anti-human TfR1 (Abcam, Cat.: ab38171; 1:1,000 dilution), anti-human/mouse TfR1 (ThermoFisher Scientific, Cat: 13-6800; 1:1,000 dilution), anti-mouse GAPDH (Cell Signaling Technology, Cat.: 2118; 1:1,000 dilution; served as loading control). Horseradish peroxidase-linked goat anti-mouse or goat anti-rabbit secondary antibodies were used (1:5,000 dilution). Densitometric quantification was performed using Image Lab 6.0.1 software (BioRad).

SUPPLEMENTARY RESULTS

Resolution of the structure of human TfR1 in complex with BCY15466

The crystal structure of human His_6_-TfR1 in complex with the Bicycle molecule BCY15466 was solved at 2.3 Å resolution (Supplementary Figure 4A-F). After molecular replacement (MR), unaccounted positive density was identified in a cavity between the apical and the protease-like domains, therefore validating previous SPR epitope binding data suggesting that the Bicycle molecule does not compete with the endogenous transferrin ligand (Supplementary Figure 4C). Binding of BCY15466 does not promote any conformational rearrangement at the level of human TfR1, with an overall Root Mean Square Deviation (RMSD) of 1.725 Å over 635 residues (measured at Cα) when compared to the human TfR1 apo crystal structure (PDB 1CX8). Structural analysis of the binding mode of BCY15466 evidenced how the Pro-Pro motif present at the beginning of the first loop likely confers the required rigidity to allocate the Bicycle molecule into the pocket. This overall arrangement allows the formation of a salt bridge between Asp3 (Bicycle) and Lys161 (human TfR1) (Supplementary Figure 4D). The second stretch of residues within the first Bicycle loop, residues 4 to 7, is involved in an extensive network of backbone-driven interactions with human TfR1, where His5 and Leu6 side chains are exposed to the solvent side of the bicyclic peptide. Further validation of the crystal structure comes from FP binding data obtained for an alanine (Ala)-scan of a very close representative of BCY15466 family (BCY15468), where the substitution of His5 and Leu6 to alanine does not abolish binding (Supplementary Figure 5). However, in the case of Leu6 there is an approximate 5-fold decrease of affinity (as measured by FP) (Supplementary Figure 5), which can be explained by the loss of a set of weak intramolecular interactions with the backbone carbonyl of Asp3. Much more important is the contribution of Gly7, since substitution to Ala perturbs massively the binding of BCY15466 to human TfR1 (Supplementary Figure 5). The presence of a glycine in this position permits the turn of loop 1 and is the only compatible residue, since the substitution to Ala or a different side chain would sterically clash with Ser159 of human TfR1. The beginning of the second loop of BCY15466 contains an Ile at position 8, which is well accommodated by a hydrophobic pocket formed by Ile190, Val192, Ile384 and Phe458 of human TfR1 (Supplementary Figure 4D, E). Additionally, the backbone carbonyl of Ile8 creates a hydrophilic interaction with the amine group of Lys382 of human TfR1 (Supplementary Figure 4D). The hydroxyl group of Ser9 of the Bicycle molecule instead forms specific hydrogen bonds with the facing Glu156 of human TfR1 and is additionally involved in stabilizing the second loop through coordination of a water molecule. Finally, the side chain of Trp10 of BCY15466 appears to form weak C-C interactions with the human TfR1 loop consisting of residues Lys193-Asp194-Ser195 (Supplementary Figure 4D). The presence of weaker electron density for the C-terminal Ala of BCY15466 hints at the partial flexibility of this residue, which however fails to establish any specific interactions with neighboring residues.

Structural analysis of the TATB scaffold shows that it sits in front of the side chain of Trp412 of human TfR1 to establish a network of interactions (Supplementary Figure 4F). Of note is the equidistant positioning of the carbonyl group on TATB with the pyrrole ring atoms of the Trp residue. Furthermore, the same carbonyl group from the scaffold forms a bridge with the N of the backbone of residue Gly413. Overall, the TATB scaffold is positioned to establish van der Waals interactions with Pro154, Trp412, Gly413 and Thr569, likely stabilizing the whole Bicycle molecule within the pocket.

Synthesis of Bicycle conjugates and characterization of their binding to human TfR1

Bicycle peptides were conjugated to ASOs and siRNAs (Figure 2). Multiple conjugations approaches have been developed to conjugate peptides to ONs (12), and for this purpose we selected a copper-free click reaction (13) between an azide and (1*R*,8*S*,9*S*)-bicyclo[6.1.0]non-4-yn-9-ylmethyl (BCN) since: 1) the conjugation is very efficient and scalable, 2) it is compatible with most functional groups, 3) peptides are easily functionalized with an azido group, 4) BCN can be effectively added to the ends of ONs by solid-phase synthesis or by solutions-phase conjugation, and 5) the linker is relatively small and should have a very small effect on the binding to human TfR1. The conjugation was performed in aqueous buffer at pH 8.5 to protect the ON from potential acidity from peptide TFA salts, and DMF was added to ascertain that both ON and peptide are soluble. Excess peptide was used to make sure all ON reacted, facilitating the SAX HPLC purification. Purified yields were generally above 70% and no novel impurities arising from the conjugation were observed. Since the PS backbone of ASOs can also contribute to strong binding to proteins (14), and since conjugation to ASOs has previously been shown to affect binding relative to an unconjugated ligand (15), we assessed the binding affinity for hTfR1 of the Bicycle-ASO conjugates. First, the Dmpk ASO was dual conjugated to the Bicycle peptide BCY17871 on the 5’-end and to Alexa Fluor 594 on the 3’-end of the ASO. This construct was then used to directly measure binding affinity to human TfR1 using BRET as detailed in the methods section. The binding affinity of BCY17871-ASO was measured to be 57 nM (Supplementary Figure 7A). Since all Bicycle peptides share the same binding epitope, the fluorescently labeled conjugate was used in future competition experiments to allow the measurement of other Bicycle-ASO conjugates, avoiding the need to synthesize all the fluorescent analogs.

Binding competition experiments using human transferrin and the Bicycle itself confirmed that Bicycle-ASO conjugates do not compete with the natural ligand transferrin for binding to the human TfR1 (Supplementary Figure 7B). These findings are consistent with the crystal structure of the human TfR1-Bicycle complex (Supplementary Figure 4C). Selected Bicycle-ASO conjugates were also tested for binding using the BRET competition assay, where they showed binding affinities (K_i_) for human TfR1 ranging from 89 to 2 nM (Figure 2A; Supplementary Figure 7C). The rank order of affinity values measured for the ASO conjugates was generally in agreement with that of the unconjugated Bicycle molecule (Figure 1), and in both cases BCY17901 had the highest binding affinity for hTfR1. SPR was used to further characterize the binding affinity of the lead Bicycle molecule BCY17901 to human TfR1 (Supplementary Table 5). The binding properties of the unconjugated BCY17901 were also compared to those of its ON conjugates, as well as to the human TfR1-binding antibody fragment OKT9 Fab’ (Supplementary Table 5). The SPR measurements showed that unconjugated BCY17901, the BCY17901-Dmpk ASO conjugate, and unconjugated OKT9 Fab’ all bind human TfR1 with similar single-digit nanomolar affinity, whereas the BCY17901-Malat1 ASO and BCY17901-Hprt siRNA conjugates have slightly weaker binding affinity for hTfR1 (Supplementary Table 5).

SUPPLEMENTARY TABLE AND FIGURE LEGENDS

**
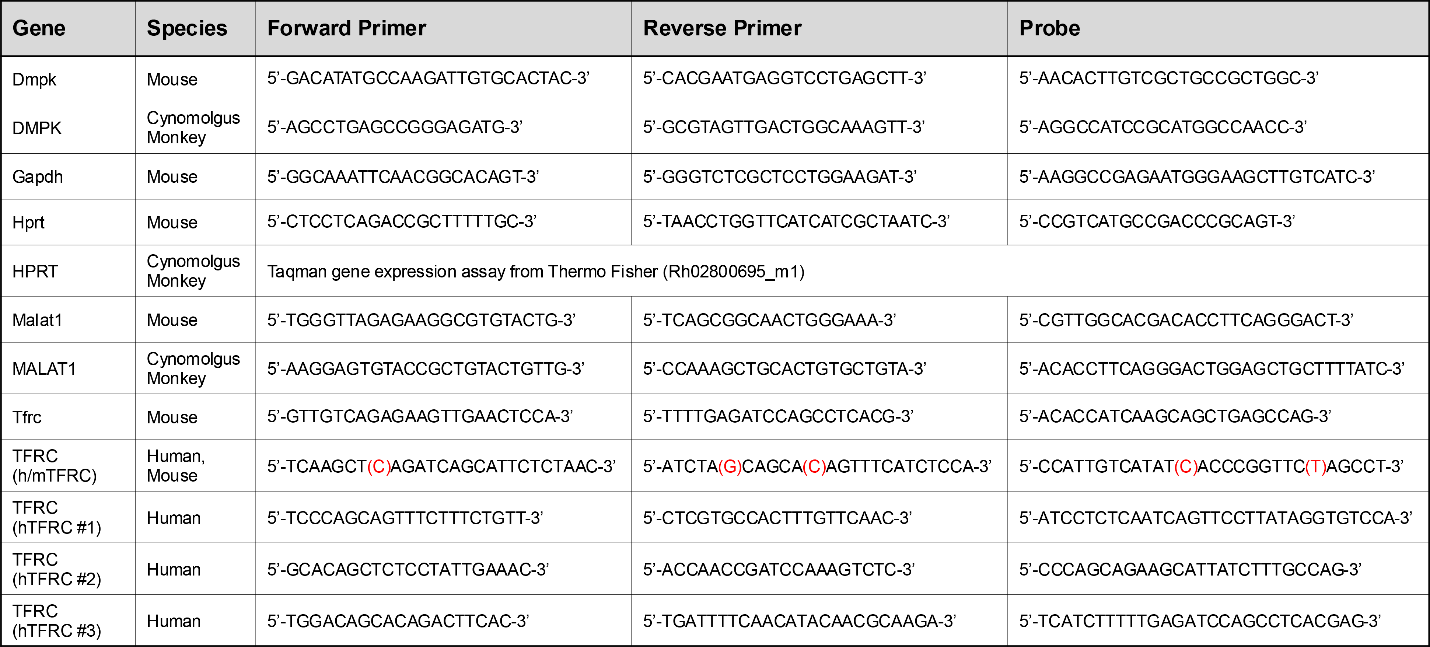
**

**Supplementary Table 1:** Sequences of primer-probe sets used in RT-qPCR assays. For the ‘h/mTFRC’ primer-probe set (PPset), the sequences used are indicated in black, which perfectly match the target human TFRC mRNA, while the putative mismatched mouse sequence is indicated in red between parentheses.


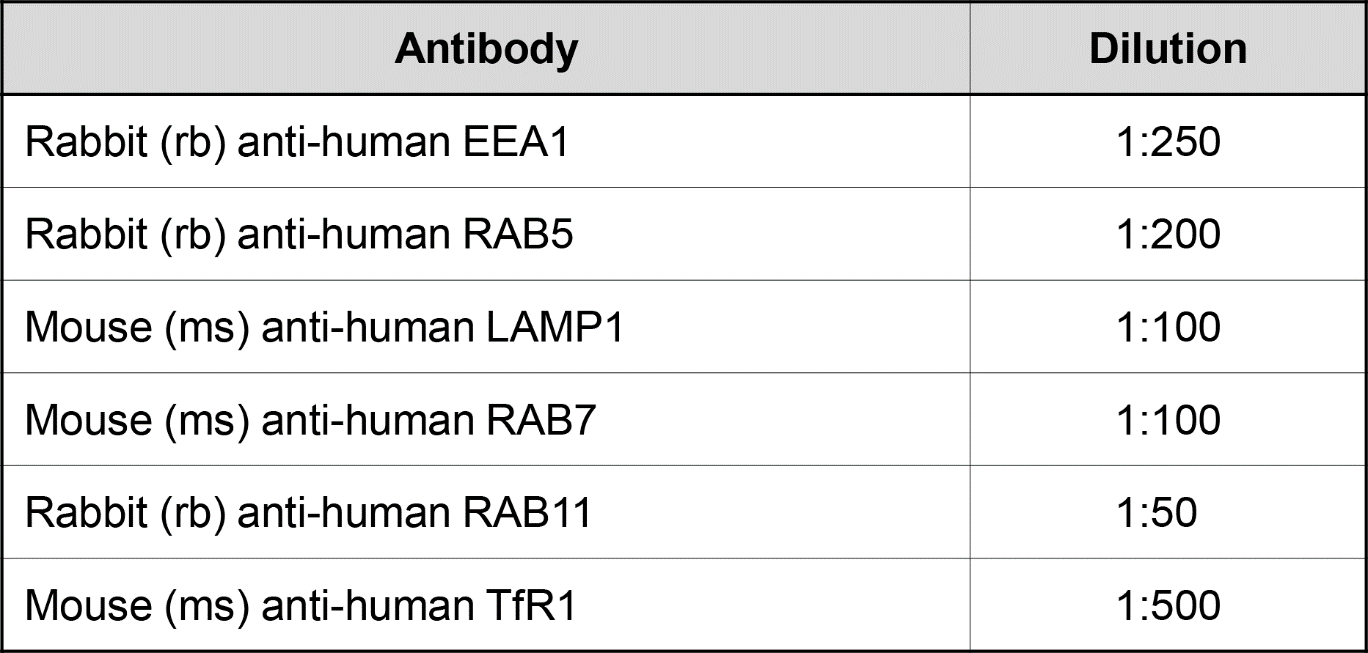


**Supplementary Table 2:** List of primary antibodies and respective dilutions used for immunostaining in HT1080 cells.


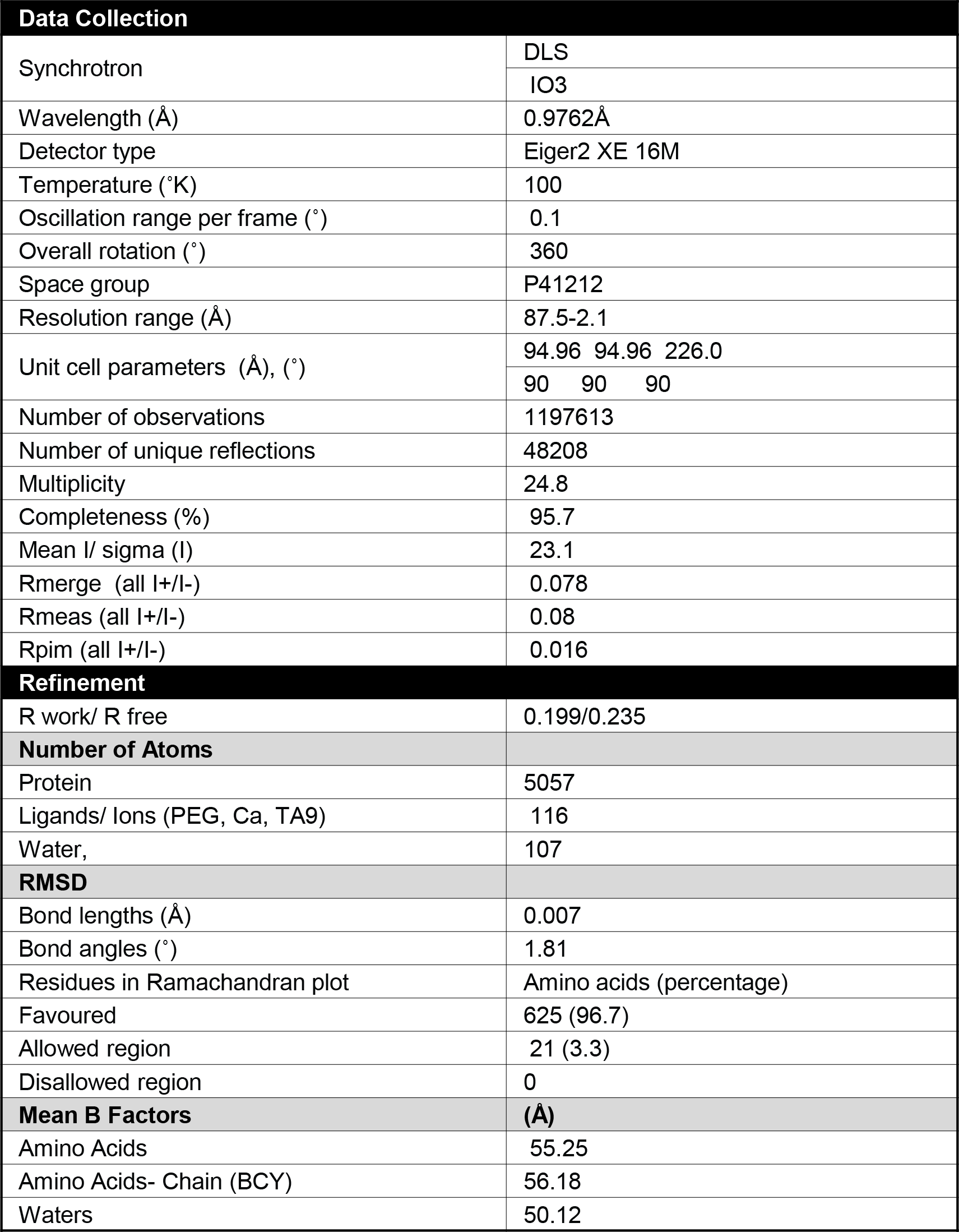


**Supplementary Table 3:** X-ray crystallography data collection, refinement, and validation statistics.


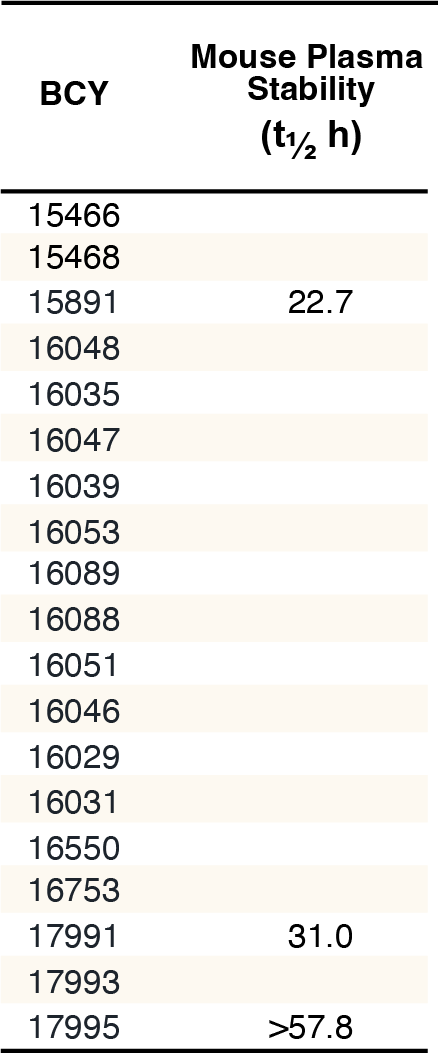


**Supplementary Table 4:** Mouse plasma stability half-lives of selected Bicycle peptides. h: hours.


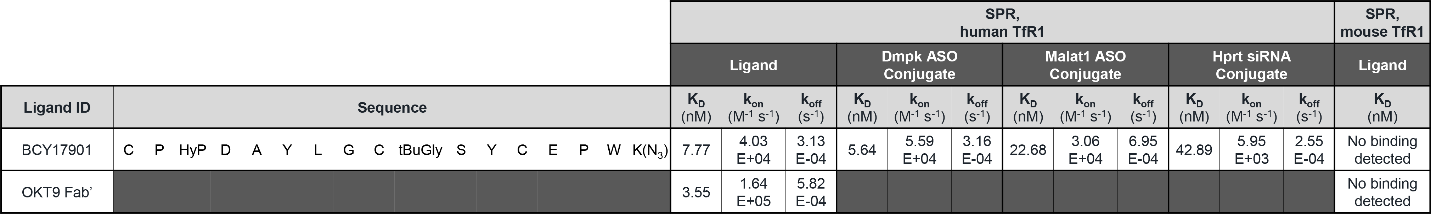


**Supplementary Table 5:** Surface plasmon resonance (SPR) measurements for BCY17901 and OKT9 Fab’, either alone or conjugated to Dmpk ASO, Malat1 ASO, and Hprt siRNA. Binding properties for human and mouse TfR1 of unconjugated ligands and ON conjugates are reported.


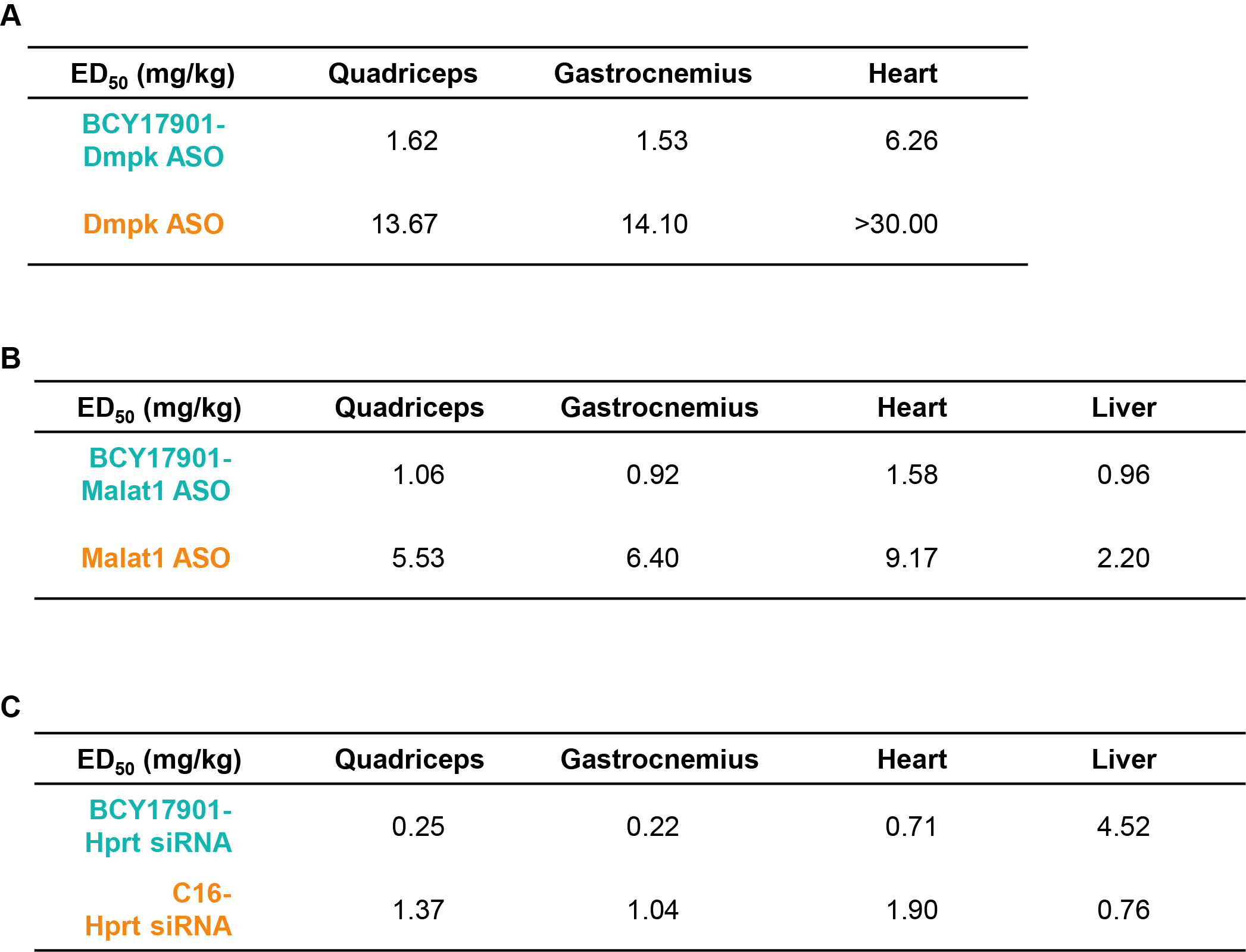


**Supplementary Table 6:** ED_50_ values associated with the graphs in Figure 4 were calculated in various skeletal muscle groups (quadriceps and gastrocnemius), heart, and liver after dosing human TfR1 heterozygote KI mice with (A) BCY17901-conjugated and unconjugated Dmpk ASO, (B) BCY17901-conjugated and unconjugated Malat1 ASO, (C) BCY17901-conjugated and lipid (palmitate, C16)-conjugated Hprt siRNA. The ED_50_ values were calculated in GraphPad Prism software using the following constraints: Top = 100, Bottom = 0, Hill slope < -1. Doses refer to the ASO or siRNA component of the LICA molecules (ASO or siRNA equivalents).


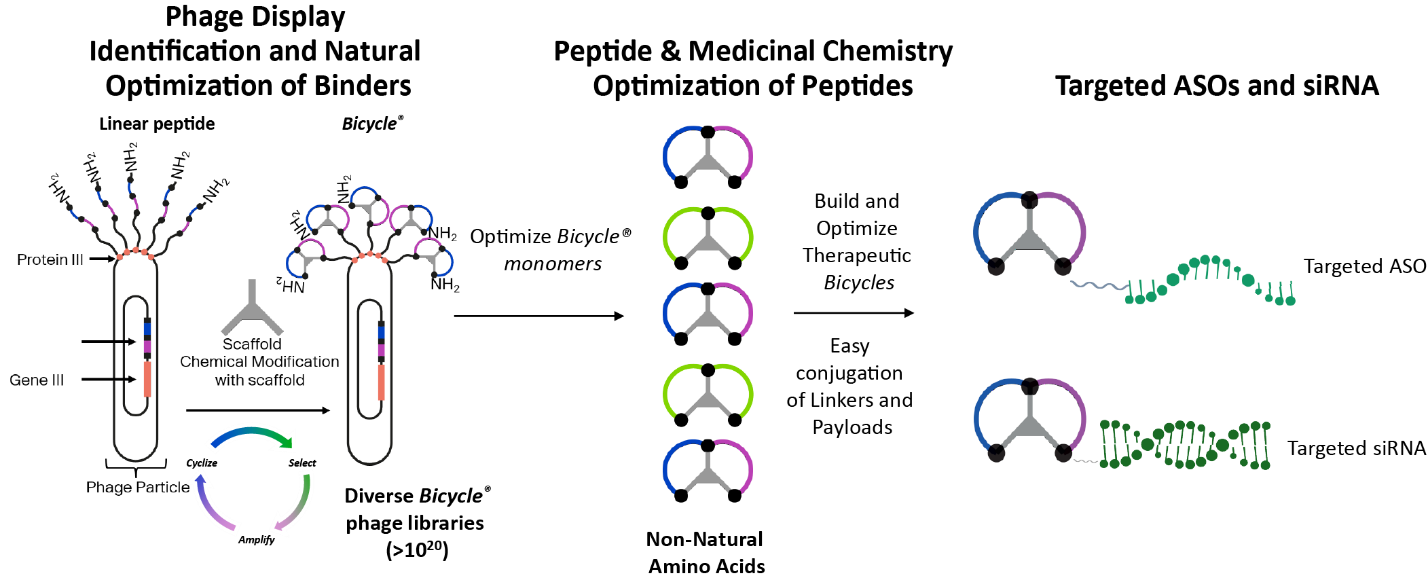


**Supplementary Figure 1:** Workflow for the identification and optimization of Bicycle lead peptides using phage display followed by peptide and medicinal chemistry, and then conjugation to ASOs and siRNAs to generate LICA molecules.


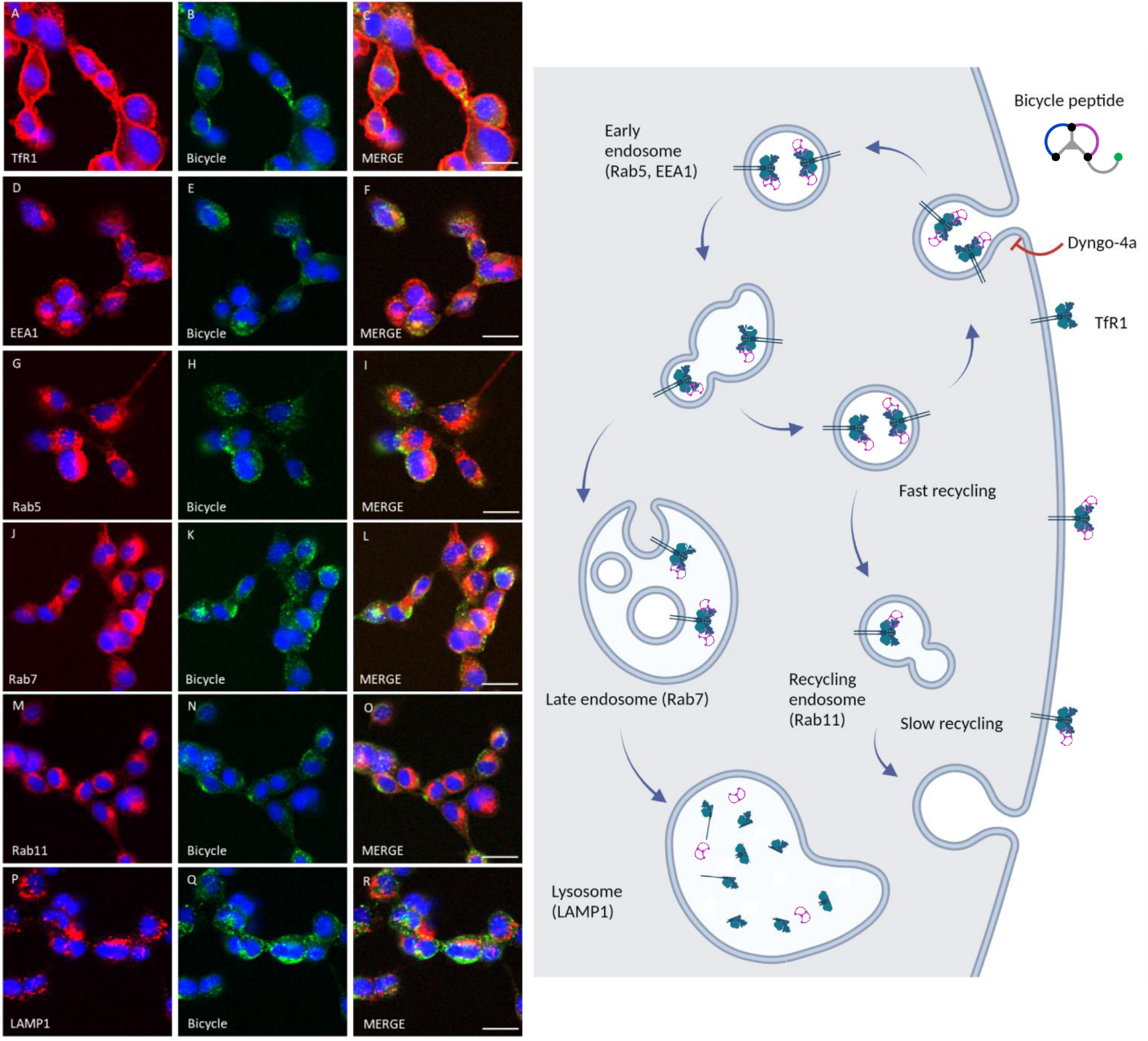


**Supplementary Figure 2:** Human TfR1-binding Bicycle molecule is internalized and co-localizes with endocytic markers. HT1080 cells were treated with 1 μM Bicycle peptide (B, E, H, K, N, Q) for 1 hour at 4°C, then transferred to 37°C for 25 minutes to allow endocytosis. After washing, the cells were fixed, permeabilized and immunostained with antibodies against human TfR1 (A), EEA1 (D), RAB5 (G), RAB7 (J), RAB11 (M) and LAMP1 (P). Cells were also stained with Hoechst in blue to reveal nuclei. Merged two-color images are shown in C, F, I, L, O, R. Scale bars correspond to 20 μm. Each image is representative of four fields from triplicate wells. Right panel shows endocytosis and recycling of Bicycle molecules. Created in BioRender. Urbonas, L. (2025) [https://BioRender.com/](https://url.us.m.mimecastprotect.com/s/-Fu_CyPkBRU9mN6LsZfnUxUicY?domain=biorender.com/)ixvyhye.


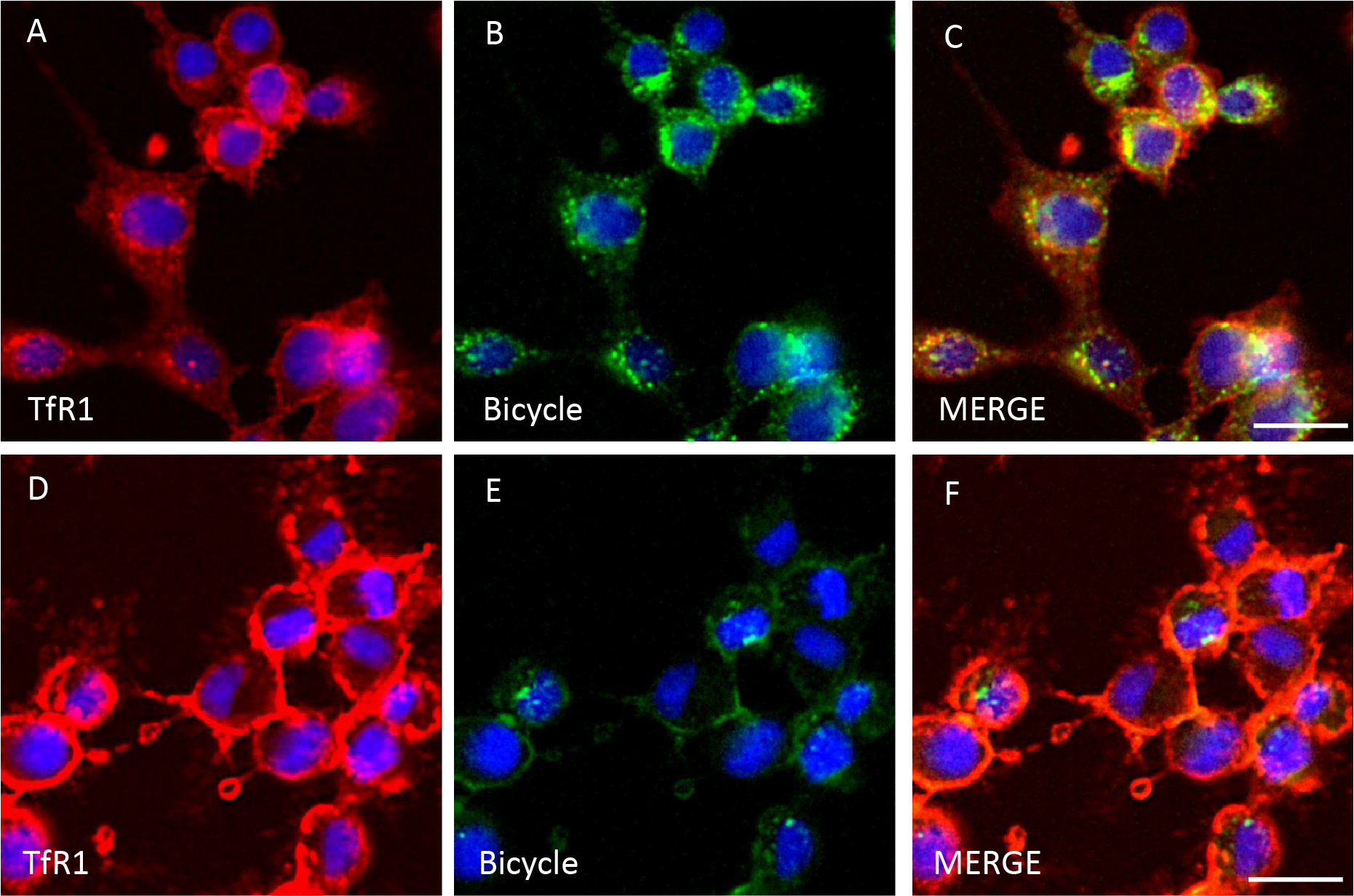


**Supplementary Figure 3:** Endocytosis of human TfR1-binding Bicycle is blocked with DYNGO-4A. HT1080 cells were treated with vehicle (A, B, C) or 30µM DYNGO-4A (D, E, F) for 30 minutes at 37°C then incubated with 1µM Bicycle peptide (B, E) for 1 hour at 4°C. Cells were then transferred to 37°C for 25 minutes to allow endocytosis. After washing, the cells were fixed, permeabilized, and immunostained with antibodies against human TfR1 (A, D, red). Cells were also stained with Hoechst (blue) to reveal nuclei. Merged two color images are shown in C and F. Scale bars correspond to 20 μm.


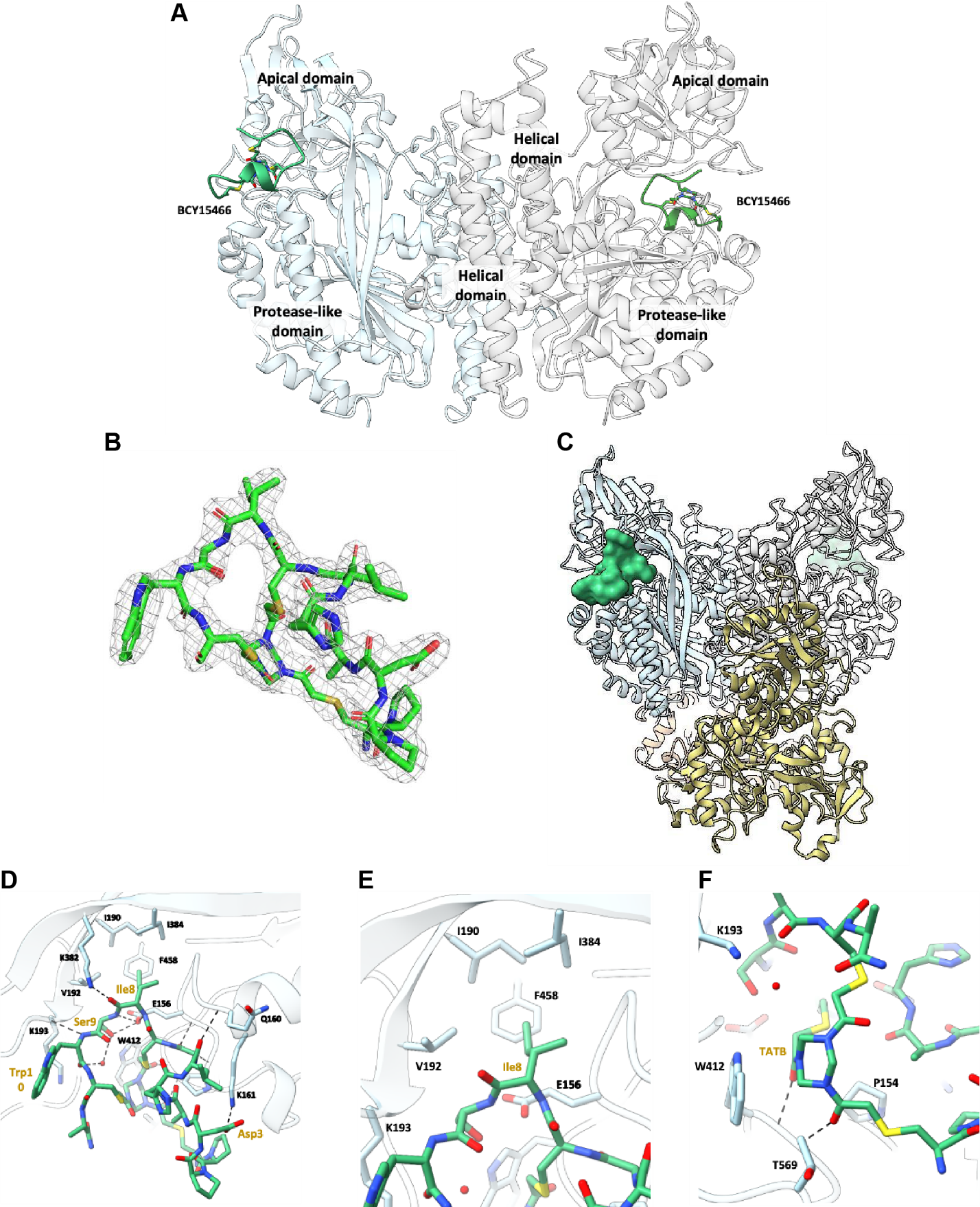


**Supplementary Figure 4:** BCY15466 binds in a pocket between the apical and the protease-like domain of human TfR1, and it does not interfere with the binding of transferrin to human TfR1. (A) Overall view of the binding pose for BCY15466 (green) on dimeric human TfR1. (B) Fit of BCY15466 into the experimental map (2Fo-Fc, contoured at 1.5σ). (C) Superimposition of the human TfR1:BCY15466 structures (PDB 9GH7, current work) with the structure of human TfR1 in complex with transferrin (PDB 3S9L). BCY15466 is green, transferrin is yellow, and human TfR1 is light cyan (monomer 1) and light grey (monomer 2). (D) Detail of the interaction network established by BCY15466 (green) on human TfR1 (light cyan). (E) Zoom-in on Ile8 of BCY15466 and its fit within a hydrophobic pocket on human TfR1. (F) Zoom-in of the interactions involving the TATB scaffold of BCY15466 and human TfR1. Hydrogen bonds are shown as black dashed lines.


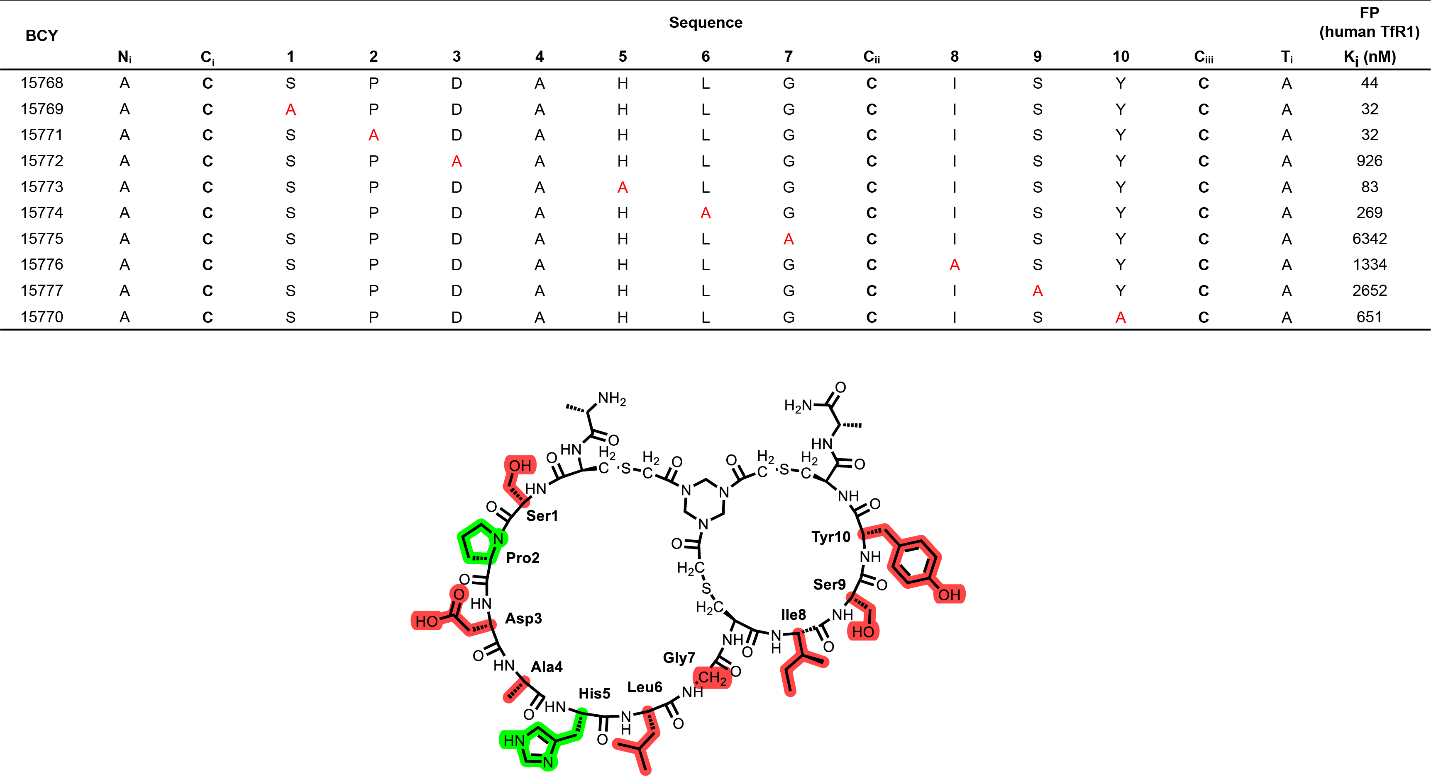


**Supplementary Figure 5:** Alanine scan of the initial Bicycle molecule to identify core residues required for binding. BCY15768 is the fluorescent version of BCY15466, and all the Bicycle molecules mentioned in the table are fluorescein conjugates for use in the fluorescence polarization (FP) assay. The structure in the bottom panel shows the interacting residues of the Bicycle molecule, as revealed by the alanine scan and X-ray crystallography, highlighted in red. Solvent facing residues, suitable for tuning molecule properties, are highlighted in green.


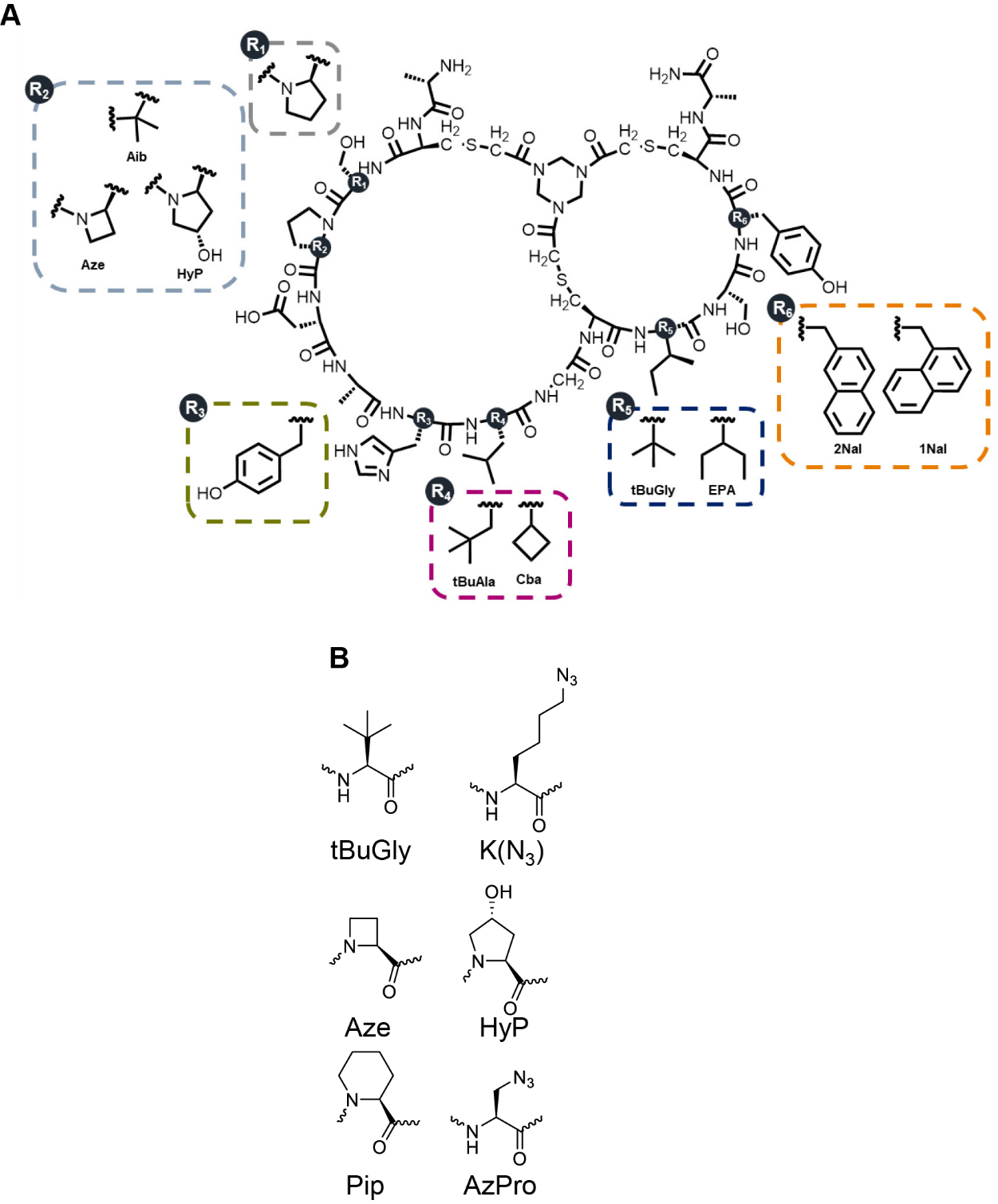


**Supplementary Figure 6:** Non-natural substitutions introduced in BCY15468. (A) Schematic representation of the position and nature of the non-natural substitutions used to enhance the binding affinity of the Bicycle peptide for human TfR1. Aib, 2-Aminoisobutyric acid; Aze, Azetidine-2-carboxylic acid; HyP, Hydroxyproline; tBuAla, tert-butylalanine; Cba, cyclobutylalanine; tBuGly, tert-butylglycine; EPA, 2-amino-3-ethylpentanoic acid; 2Nal, 3-(2-naphthyl)-alanine; 1Nal, 3-(1-naphthyl)-alanine. (B) Chemical structures of the non-natural amino acids used in this study.


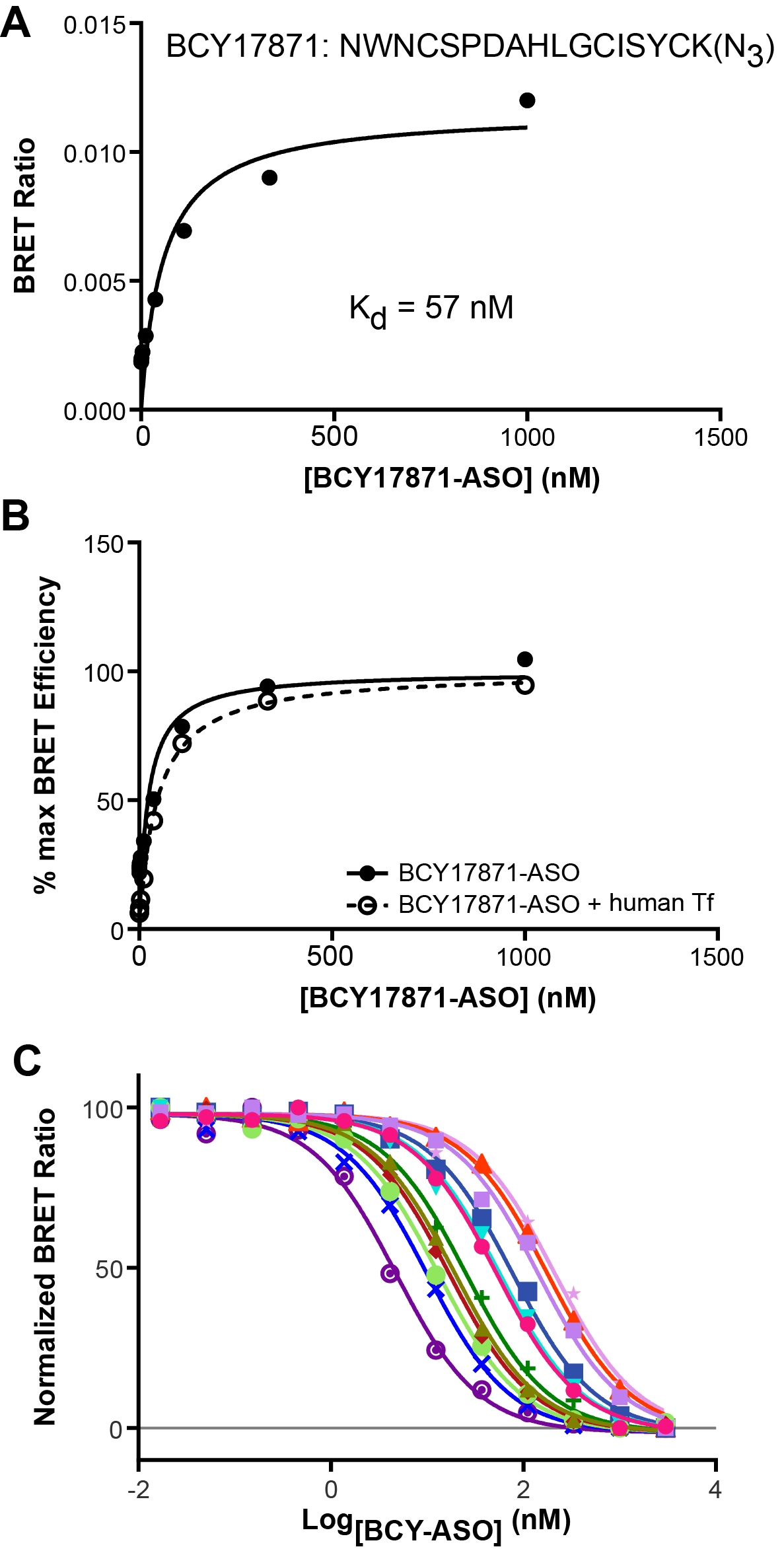


**Supplementary Figure 7:** Binding affinities for human TfR1 of twelve different Bicycle-Dmpk-ASO conjugates. (A) Direct determination of binding affinity using BRET for Alexa Fluor 594 labeled Bicycle-ASO conjugate. (B) Competition binding with transferrin. (C) Dose-response curves for competition experiments with the Bicycle-ASO conjugates listed in Figure 1.


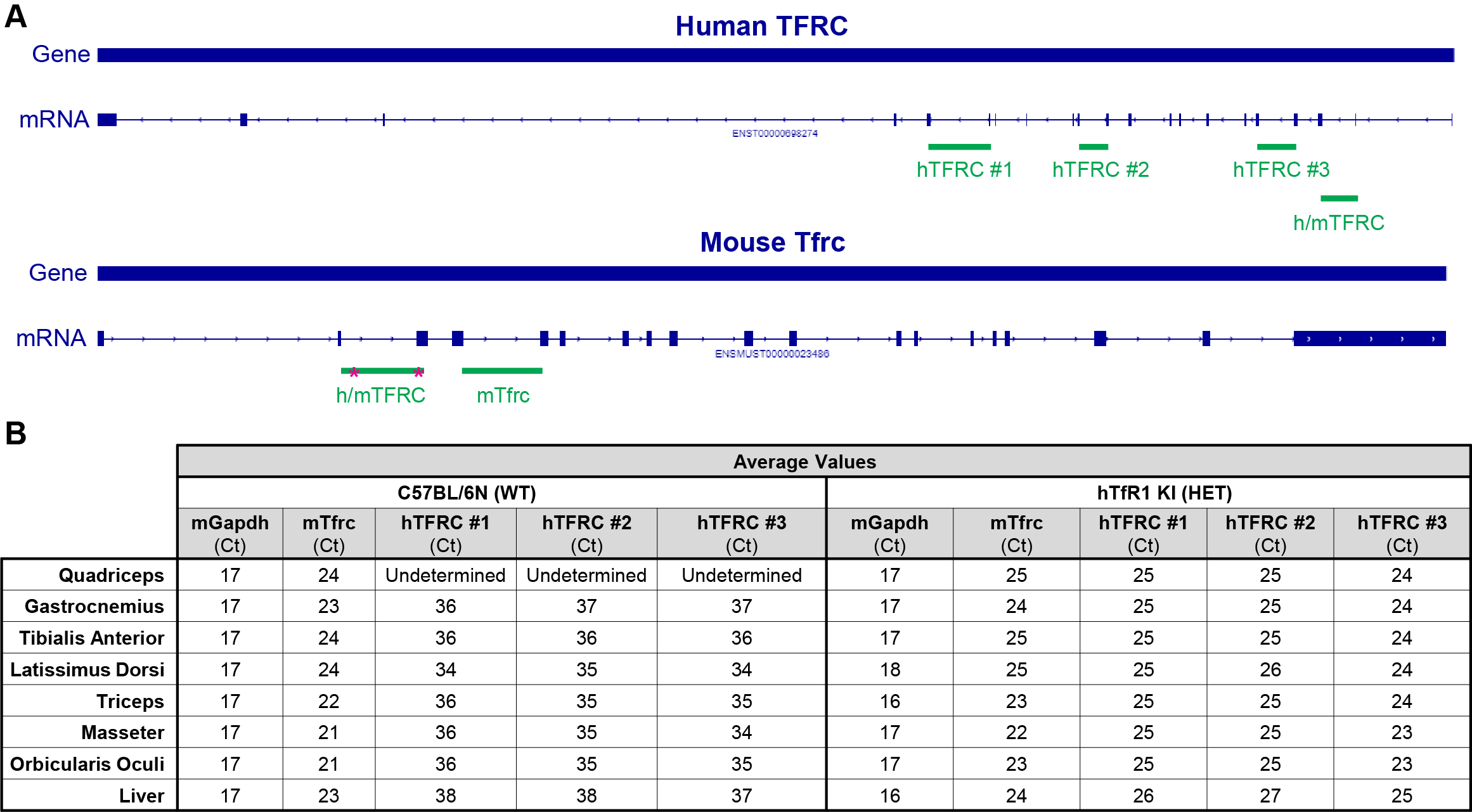


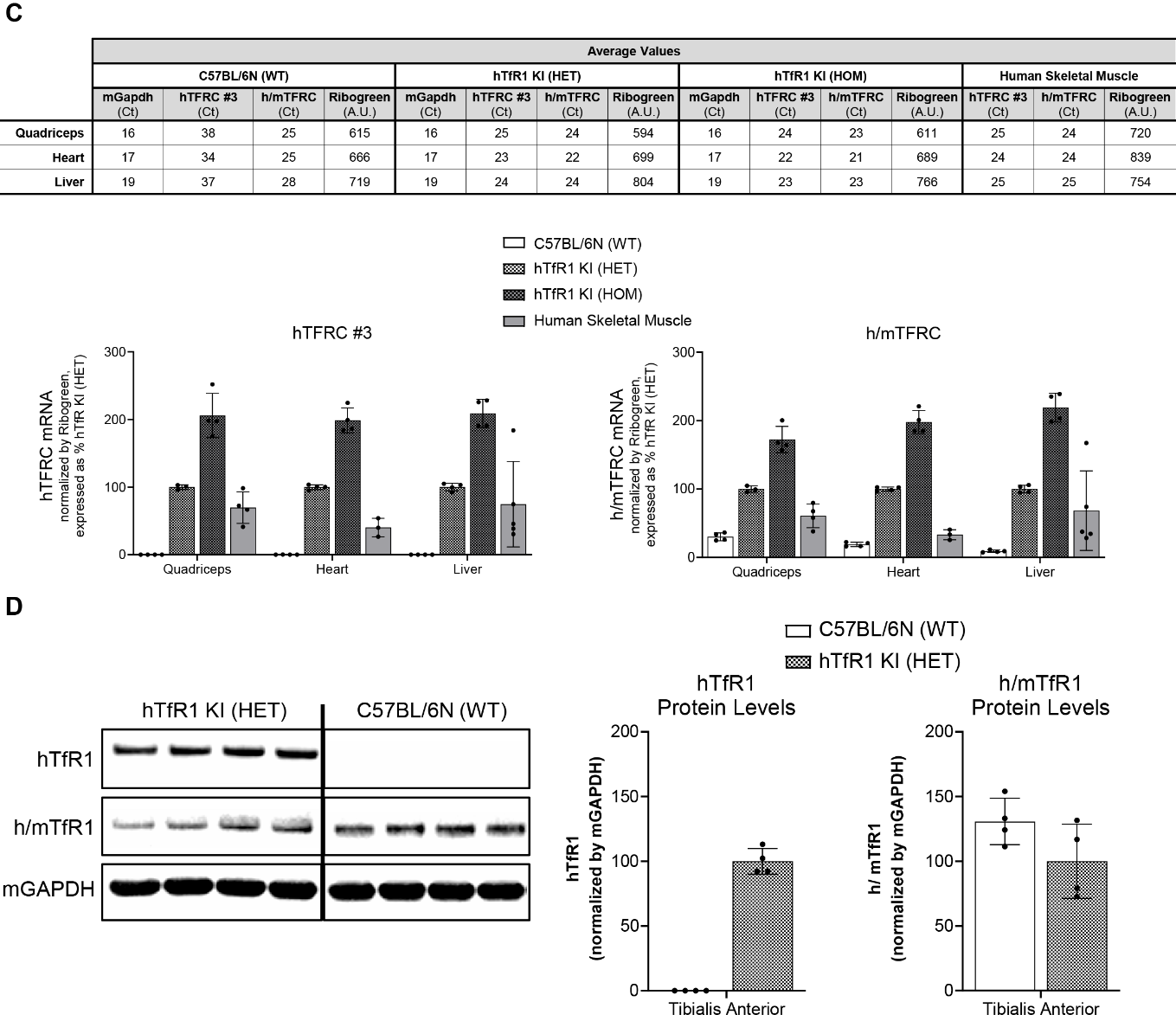


**Supplementary Figure 8:** Characterization of TfR1 expression in human TfR1 KI mice. (A) Schematic representation of the location of the PPsets used to measure human (‘h’ prefix) and mouse (‘m’ prefix) TFRC mRNA expression by RT-qPCR. The ‘h/m’ PPset amplifies both human and mouse TFRC mRNA, despite having mismatches for the target mouse mRNA sequence (as described in Supplementary Table 1; the red asterisks indicate that the PPset has mismatches). (B) Average Ct values for either mouse Tfrc or human TFRC compared to mouse Gapdh (housekeeping gene) in various tissues from C57BL/6N wild-type (WT) mice and human TfR1 KI heterozygote (HET) mice. Three different PPsets spanning the human TFRC transcript were utilized. (C) Table, top panel: average TFRC Ct values measured by RT-qPCR using either a human-specific (hTFRC #3) or a human/mouse cross-reactive (h/mTFRC) PPset, compared to mouse Gapdh (housekeeping gene) or Ribogreen (quantifies total input RNA) in various tissues from WT mice, human TfR1 KI HET or homozygote (HOM) mice, and human skeletal muscle. A.U.: arbitrary units; refers to Ribogreen fluorescence signal relative to a standard curve. Graphs, bottom panel: the graphs report the expression data for human TFRC (hTFRC #3), and for human plus mouse TFRC (h/mTFRC) in the indicated mouse genotypes. Human skeletal muscle samples were included as reference, to demonstrate that the KI mice express robust levels of human TFRC. (D) Left panel: Western blot analysis in quadriceps muscle from human TfR1 KI HET and WT mice. A human-specific (‘h’) or a human/mouse cross-reactive (‘h/m’) primary antibody for TfR1 were used. Mouse GAPDH was detected as housekeeping loading control. Right panel: quantification of the Western blot images.


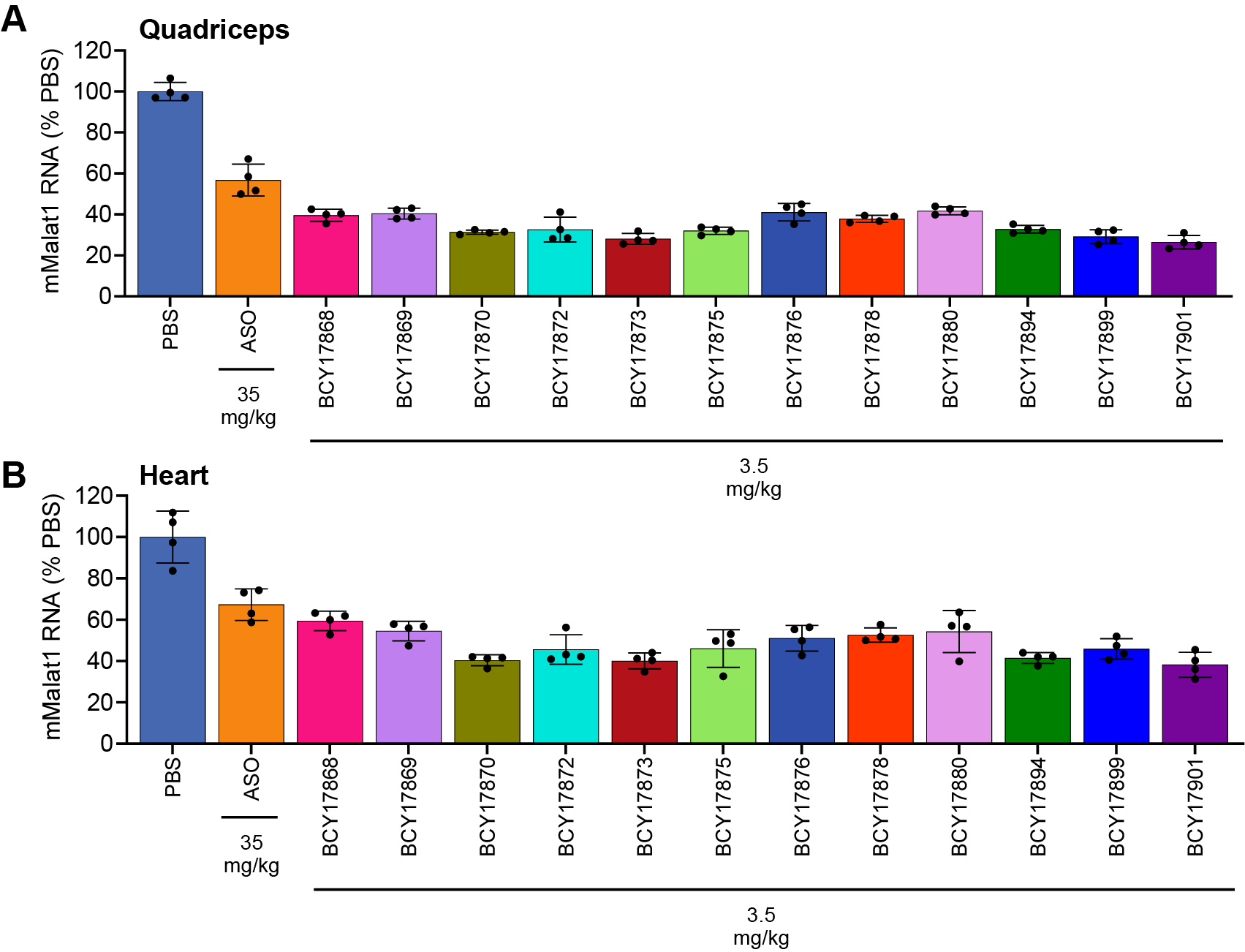


**Supplementary Figure 9:** Twelve different Bicycle peptides were conjugated to mouse Malat1 ASO and dosed intravenously in human TfR1 heterozygote KI mice at 3.5 mg/kg ASO equivalents. A group of mice were dosed with the unconjugated Malat1 ASO at 35 mg/kg (orange). The graphs report the mouse Malat1 (mMalat1) RNA levels measured by RT-qPCR in (A) quadriceps muscle and (B) heart, expressed as percentage of the PBS-treated animals (vehicle control), after normalization using mouse Gapdh as the housekeeping gene. Error bars indicate standard deviation.


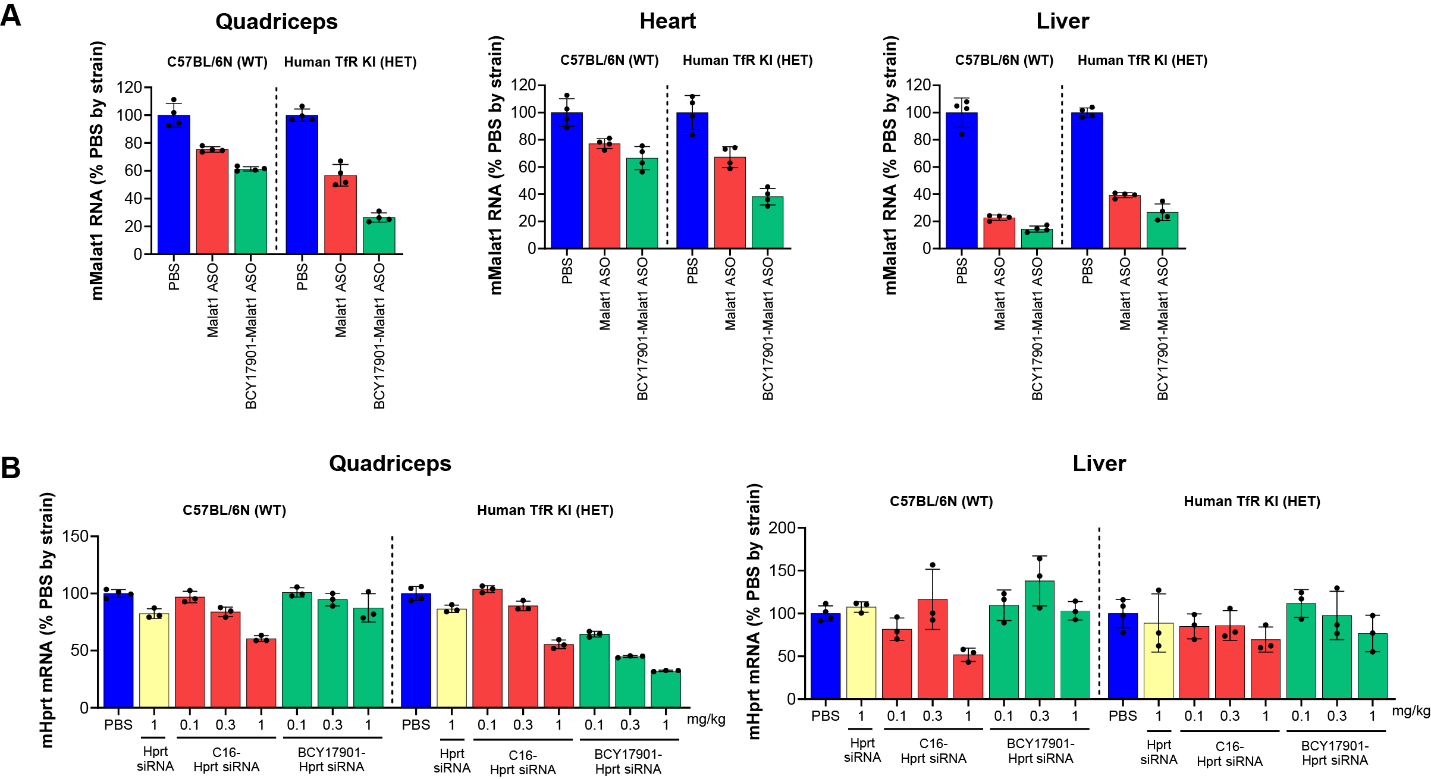


**Supplementary Figure 10:** The activity of BCY17901-conjugated Malat1 ASO and Hprt siRNA is largely blunted in the skeletal and cardiac muscle of wild-type mice compared to human TfR1 KI mice. Additionally, Bicycle-siRNA molecules have a liver-sparing property that is not seen with the unconjugated or BCY17901-conjugated Malat1 ASO. (A) Wild-type (WT) C57BL/6N mice and human TfR1 heterozygote (HET) KI mice were dosed intravenously with either PBS (dark blue), 3 mg/kg Malat1 ASO (red), or 3 mg/kg BCY17901-conjugated Malat1 ASO (green). (B) Wild-type (WT) C57BL/6N mice and human TfR1 heterozygote (HET) KI mice were dosed intravenously with either PBS (dark blue), 1 mg/kg unconjugated Hprt siRNA (yellow), lipid (palmitate, C16)-conjugated Hprt siRNA (red, various dose-levels), or BCY17901-conjugated Hprt siRNA (green, various dose-levels). The graphs report (A) the mouse Malat1 (mMalat1) RNA levels, or (B) the mouse Hprt (mHprt) mRNA levels measured by RT-qPCR, expressed as percentage of the strain-matched PBS-treated animals (vehicle control) after normalization using mouse Gapdh as the housekeeping gene. Error bars indicate standard deviation. Doses refer to the ASO or siRNA component of the LICA molecules (ASO or siRNA equivalents).


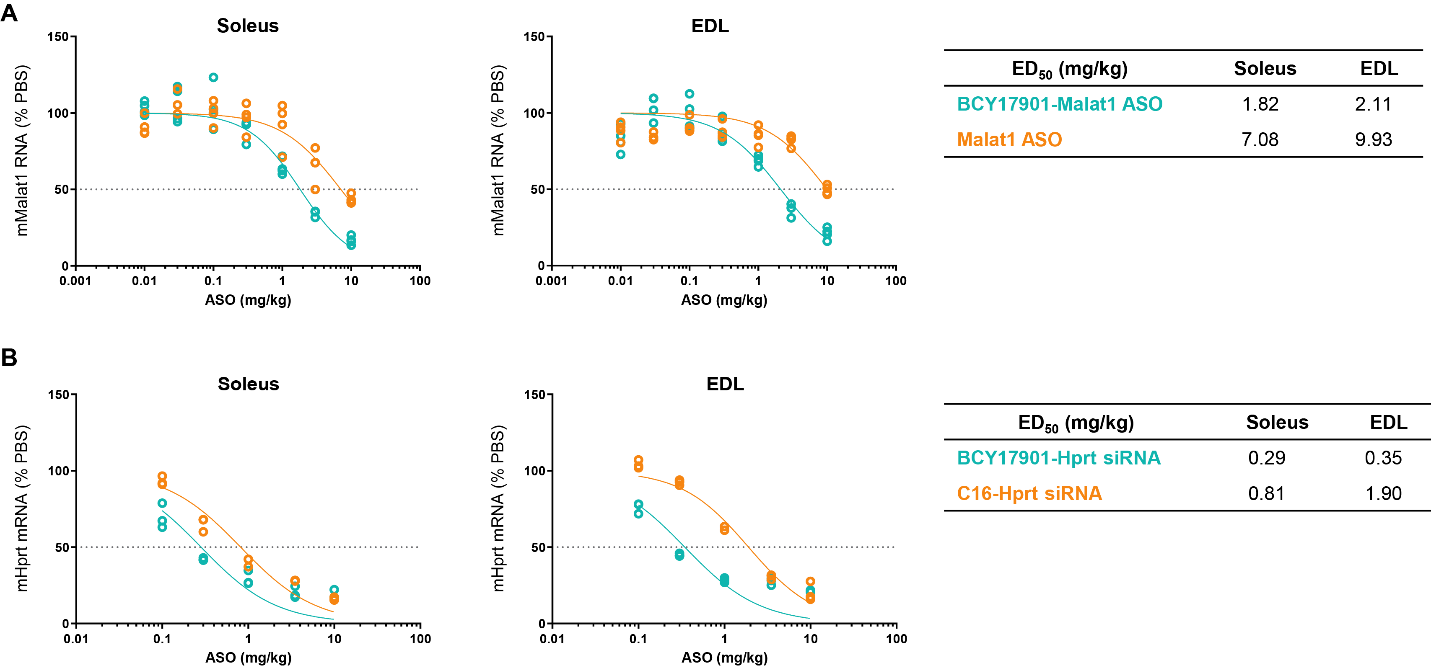


**Supplementary Figure 11:** Conjugation of Malat1 ASO and Hprt siRNA to BCY17901 improves potency in soleus muscle (slow-twitch, predominantly type I myofibers) and extensor digitorum longus (EDL) muscle (fast-twitch, predominantly type II myofibers) of human TfR1 heterozygote KI mice. Dose-dependent target knockdown was measured by RT-qPCR after intravenous injection of (A) Malat1 ASO, and (B) Hprt siRNA conjugated to BCY17901. Unconjugated ASO and a lipid (palmitate, C16)-conjugated Hprt siRNA were included in the study to compare their potency versus the BCY17901-conjugated counterparts. Data is expressed as percentage of target RNA level compared to PBS-treated (vehicle control) mice, after normalization using mouse Gapdh as housekeeping gene. Each open dot represents one animal. The ED_50_ values listed in the tables next to the respective graphs were calculated in GraphPad Prism software using the following constraints: Top = 100, Bottom = 0, Hill slope < -1. Doses refer to the ASO or siRNA component of the LICA molecules (ASO or siRNA equivalents).


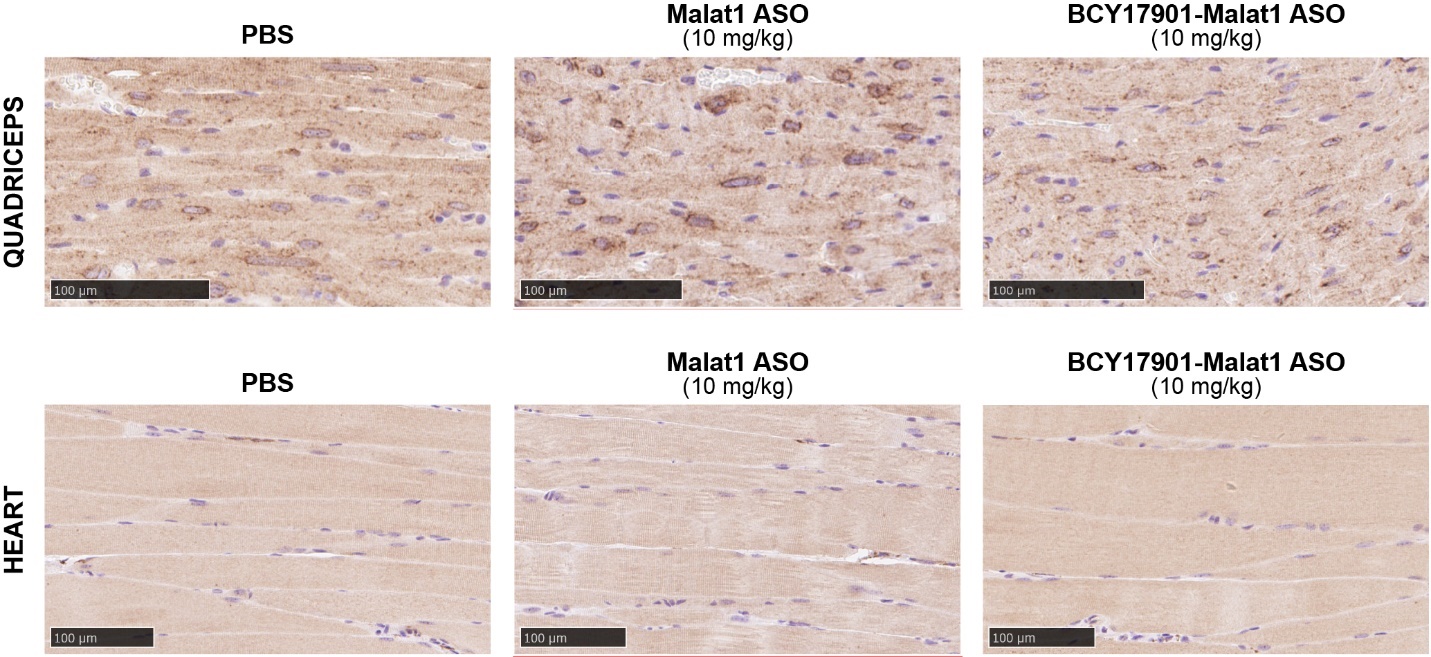


**Supplementary Figure 12:** Repeat dosing with BCY17901-ASO conjugate does not reduce TfR1 protein levels on the surface of skeletal and cardiac muscle cells. IHC for human TfR1 (brown stain) in histological sections of quadriceps muscle and heart from human TfR1 KI mice dosed intravenously with either PBS (vehicle control), unconjugated Malat1 ASO at 10 mg/kg, or BCY17901-conjugated Malat1 ASO at 10 mg/kg ASO equivalents. Scale bars: 100 μm.


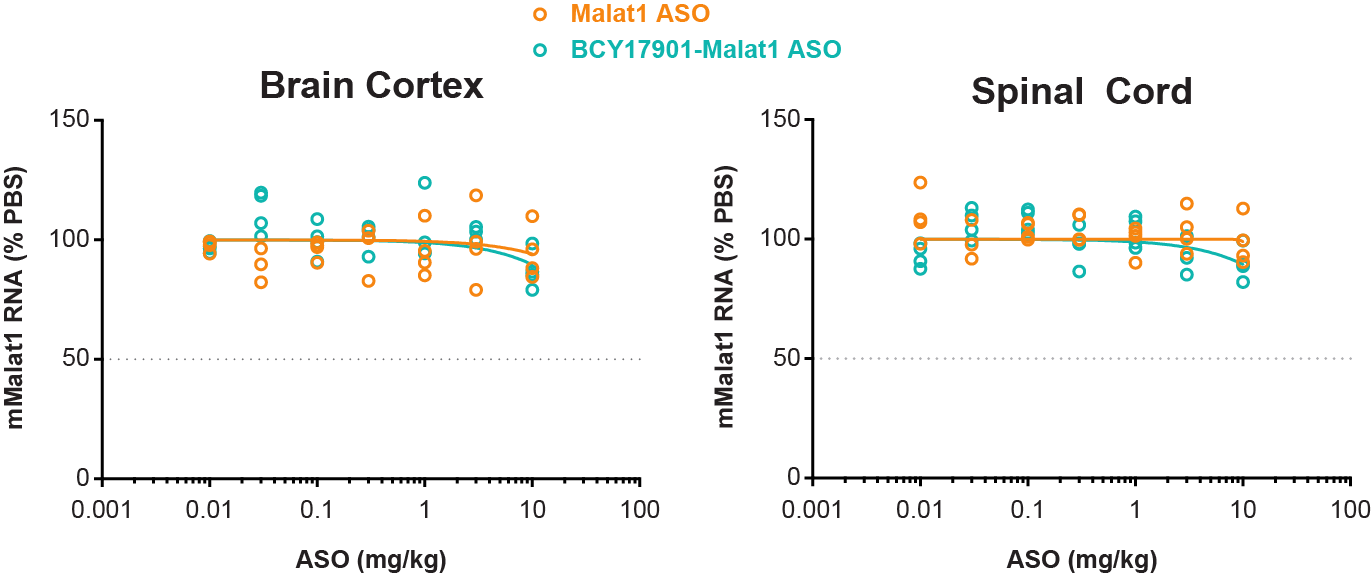


**Supplementary Figure 13:** Systemically dosed BCY17901-Malat1 ASO does not result in Malat1 RNA knockdown in CNS tissues. Malat1 RNA levels measured by qPCR in brain cortex and spinal cord of human TfR1 KI mice after intravenous administration of either unconjugated or BCY17901-conjugated Malat1 ASO at the indicated ASO equivalent doses.


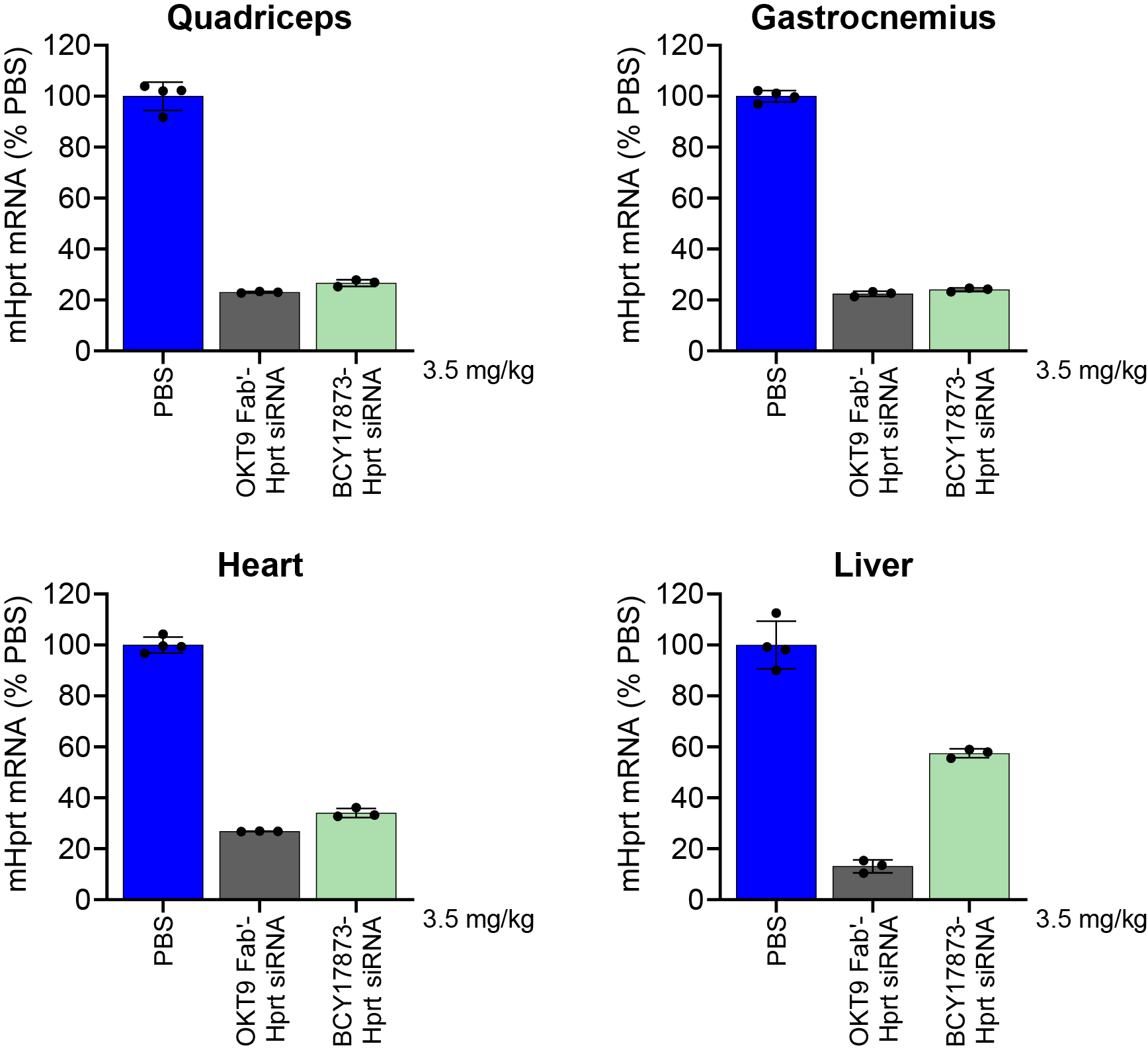


**Supplementary Figure 14:** Partial liver-sparing properties of BCY-siRNA compared to OKT9 Fab’-siRNA conjugates. Human TfR1 heterozygote KI mice were dosed intravenously with either PBS, 3.5 mg/kg OKT9 Fab’-siRNA conjugate, or 3.5 mg/kg BCY17873-conjugated Hprt siRNA. The graphs report the mouse Hprt (mHprt) mRNA levels measured by RT-qPCR in skeletal muscle (quadriceps and gastrocnemius), heart, and liver. Target mRNA levels are expressed as percentage of the PBS-treated animals (vehicle control), after normalization using mouse Gapdh as the housekeeping gene. Error bars indicate standard deviation.


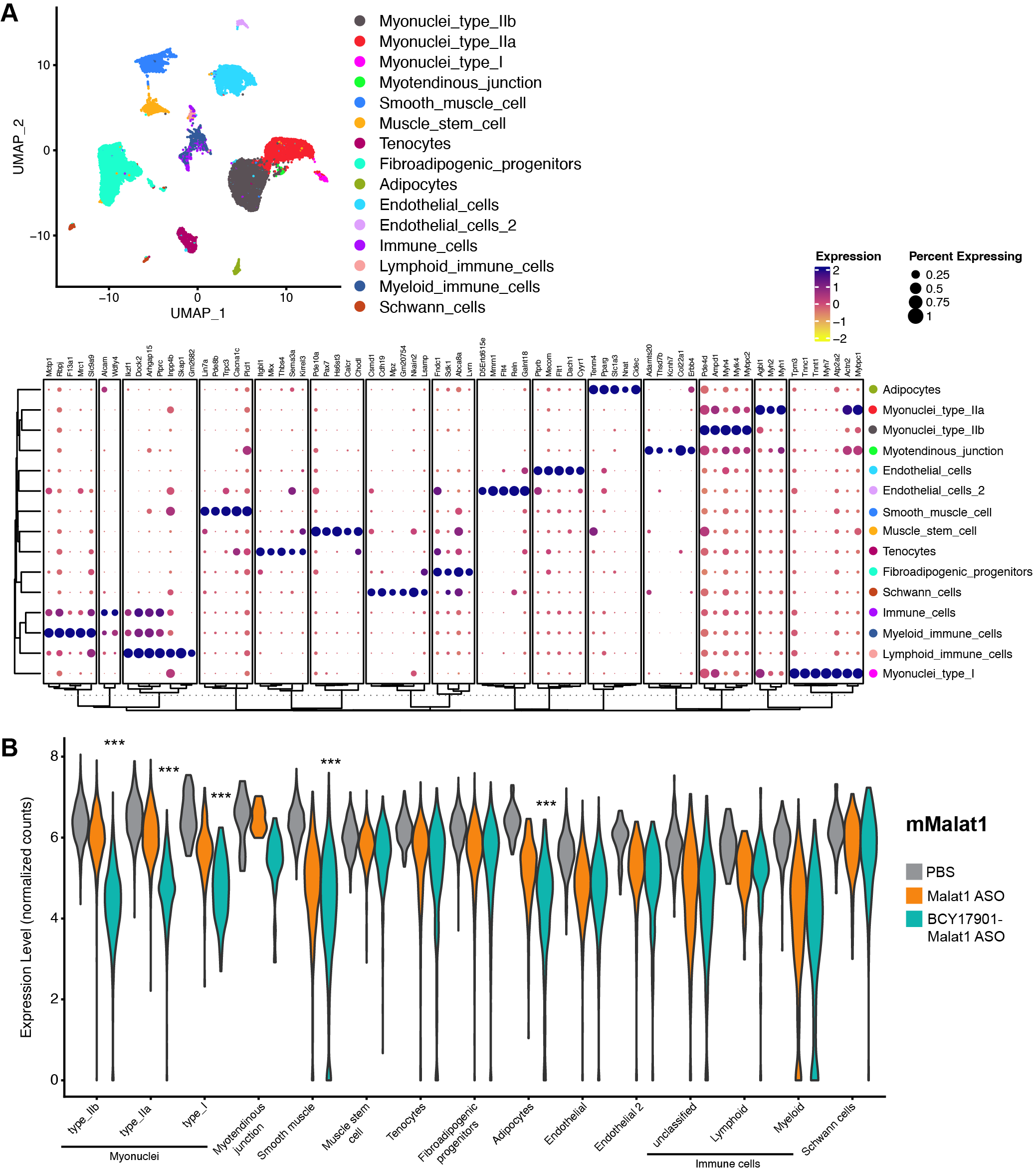


**Supplementary Figure 15:** (A) Top: annotation of cell types identified in single-nucleus RNA sequencing (snRNAseq) analysis of gastrocnemius muscles plotted in a UMAP embedding. Bottom: dot plot visualization of marker genes expression for each cell type cluster identified in snRNAseq of gastrocnemius muscles. Marker genes are grouped by expression similarity. Dots are color coded based on the expression level. Dot size is correlated with the percent cells expressing the marker gene in each cell type. (B) Violin plot of mMalat1 expression level in different cell types identified in snRNAseq of gastrocnemius muscles of mice treated with PBS (vehicle, blue), unconjugated ASO (ASO, red), and BCY17901-conjugated ASO (BCY-ASO, green). Data from n = 8 mice (2 mice dosed with PBS vehicle control, 3 mice with unconjugated Malat1 ASO, and 3 mice with BCY17901-conjugated Malat1 ASO). *** indicate adjusted p-value < 0.001 in differential expression test comparing unconjugated and BCY17901-conjugated Malat1 ASOs (MAST(16) with Bonferroni correction).


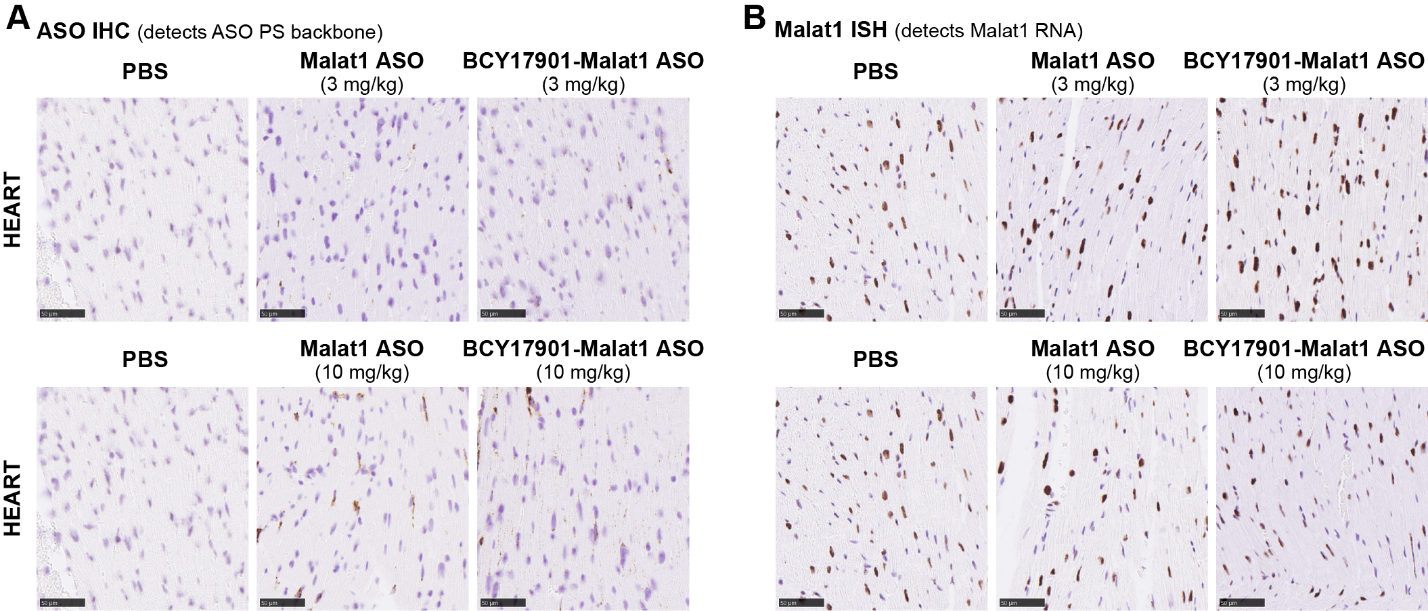


**Supplementary Figure 16:** ASO conjugation to BCY17901 improves ASO uptake, activity in cardiomyocytes of human TfR1 KI mice. Representative images of (A) ASO IHC (labels ASO in brown), and (B) Malat1 ISH (labels Malat1 RNA in brown) in histological sections of heart from human TfR1 KI mice dosed with either PBS (vehicle control), unconjugated Malat1 ASO, or BCY17901-conjugated Malat1 ASO. The top panels report the results for the 3 mg/kg ASO equivalent dose-level, whereas the bottom panels report the results for the 10 mg/kg dose-level. Scale bars: 50 μm.


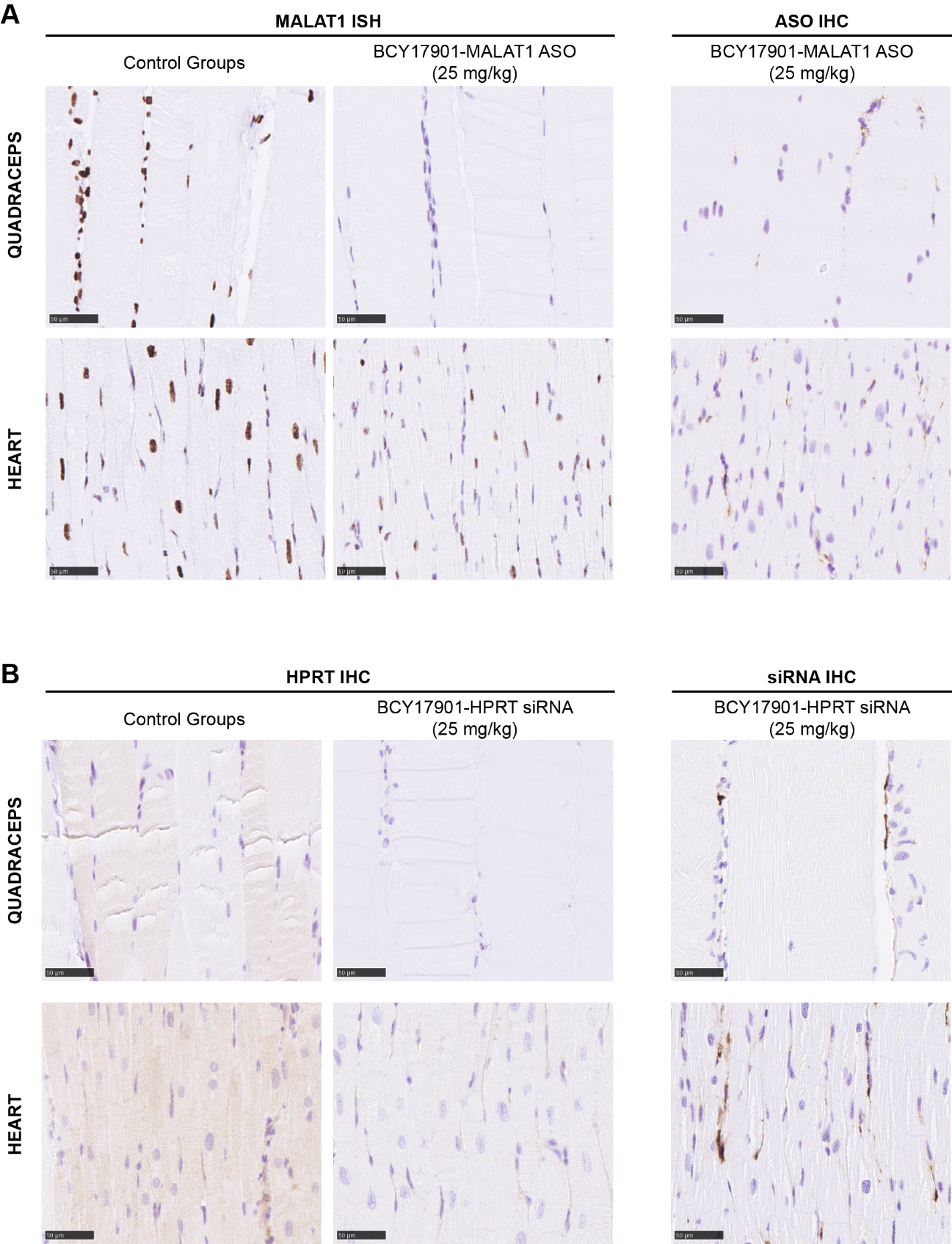


**Supplementary Figure 17:** BCY17901-conjugated MALAT1 ASO and HPRT siRNA reduce target RNA in myofibers and cardiomyocytes of non-human primates. Representative images of quadriceps muscle and heart histological sections from NHPs dosed with (A) Malat1 ASO or (B) Hprt siRNA. The MALAT1 RNA levels were assessed using ISH (MALAT1 ISH, brown staining). The HPRT protein levels were assessed using IHC (HPRT IHC, brown staining). IHC was used to visualize the localization of ASO and siRNA (ASO IHC and siRNA IHC, visible as brown staining). Scale bars: 50 μm.


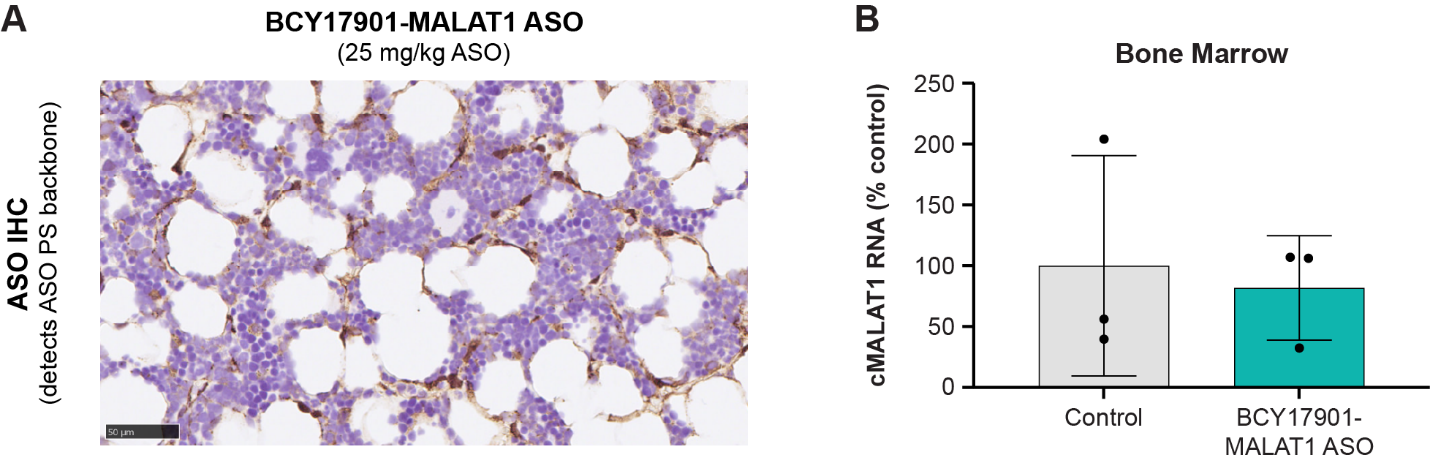


**Supplementary Figure 18:** BCY17901-Malat1 ASO distribution and activity in the bone marrow of NHP. A) ASO IHC to assess the biodistribution pattern of BCY17901-conjugated Malat1 ASO in the bone marrow of NHP. IHC was used to detect the PS backbone of the ASO (visible as brown staining) in the tissue. Scale bars: 50 μm. B) Cynomolgus monkey MALAT1 (cMALAT1) RNA level measured by RT-qPCR in bone marrow of NHPs dosed by intravenous infusion with 25 mg/kg BCY17901-conjugated MALAT1 ASO. The expression data was normalized using RiboGreen and is reported as percentage of control-treated animals (NHPs dosed with non-MALAT1 or non-HPRT targeting compounds). Error bars indicate standard deviation.


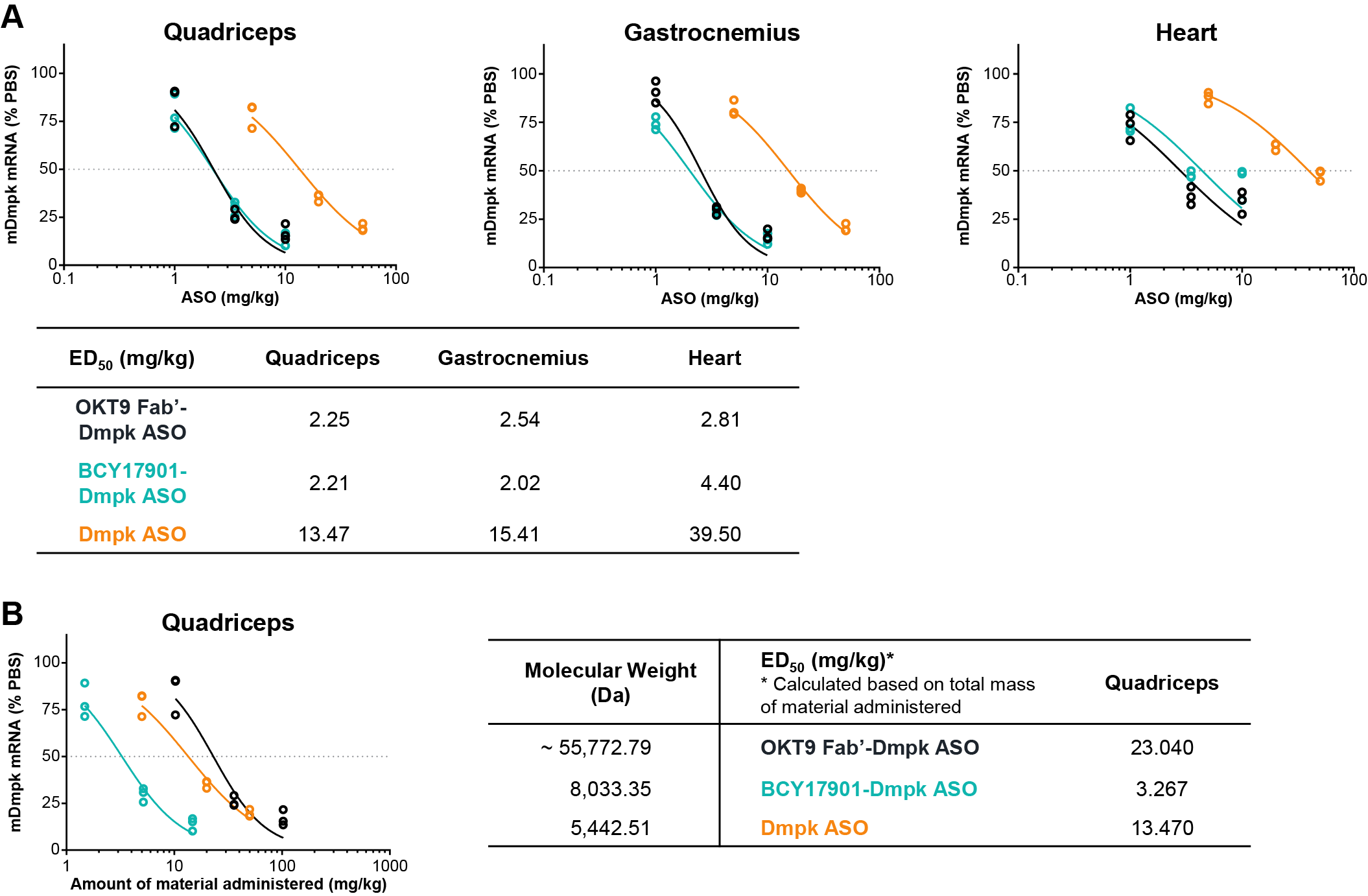


**Supplementary Figure 19:** Compared to antibody fragments (Fab, ~50 kDa), the smaller Bicycle peptides (~2 kDa) allow the reduction of the total dose of drug that needs to be administered in order to deliver the same amount of ASO to skeletal muscle and heart. (A) Dose-dependent knockdown of mouse Dmpk (mDmpk) mRNA was measured by RT-qPCR in various skeletal muscle groups (quadriceps and gastrocnemius), and heart of human TfR1 KI mice after intravenous dosing of OKT9 Fab’-Dmpk ASO (black), BCY17901-Dmpk ASO (green), or unconjugated Dmpk ASO (red). Doses refer to the ASO component of the LICA molecules (ASO equivalents). BCY17901 and OKT9 Fab’ are two ligands that bind to human TfR1 with similar affinity, as measured by SPR (Supplementary Table 3), but have very different molecular weights. Both BCY17901-ASO or OKT9 Fab’-ASO conjugates knock down mouse Dmpk mRNA levels in skeletal muscle and heart with similar potency (ED_50_ values) when the dose is calculated in ASO equivalents. (B) Dmpk mRNA levels measured in quadriceps muscle by RT-qPCR (y axis, same values as in panel A) plotted against the total dose of compound (x axis). Therefore, ED_50_ values were calculated by factoring in the molecular weight of the whole conjugates, enabling the comparisons of their potency based on the total mass of material administered rather than ASO equivalents. When the molecular weight of the two ligands is factored into the calculation of the dose of whole conjugate administered, the approximately 7-fold potency advantage of the smaller Bicycle peptide conjugate versus the Fab’ conjugate becomes evident.

The data is expressed as percentage target mRNA level compared to PBS-treated (vehicle control) mice, after normalization using mouse Gapdh as housekeeping gene. Each open dot represents one animal. The ED_50_ values listed in the tables next to the respective graphs were calculated in GraphPad Prism software using the following constraints: Top = 100, Bottom = 0, Hill slope < -1.

SUPPLEMENTARY REFERENCES

1. Vonrhein, C., Flensburg, C., Keller, P., Sharff, A., Smart, O., Paciorek, W., Womack, T. and Bricogne, G. (2011) Data processing and analysis with the autoPROC toolbox. *Acta Crystallogr D Biol Crystallogr*, **67**, 293-302.

2. McCoy, A.J., Grosse-Kunstleve, R.W., Adams, P.D., Winn, M.D., Storoni, L.C. and Read, R.J. (2007) Phaser crystallographic software. *Journal of Applied Crystallography*, **40**, 658-674.

3. Emsley, P., Lohkamp, B., Scott, W.G. and Cowtan, K. (2010) Features and development of Coot. *Acta Crystallogr D Biol Crystallogr*, **66**, 486-501.

4. Murshudov, G.N., Vagin, A.A. and Dodson, E.J. (1997) Refinement of macromolecular structures by the maximum-likelihood method. *Acta Crystallogr D Biol Crystallogr*, **53**, 240-255.

5. Meng, E.C., Goddard, T.D., Pettersen, E.F., Couch, G.S., Pearson, Z.J., Morris, J.H. and Ferrin, T.E. (2023) UCSF ChimeraX: Tools for structure building and analysis. *Protein Sci*, **32**, e4792.

6. Mudd, G.E., Brown, A., Chen, L., van Rietschoten, K., Watcham, S., Teufel, D.P., Pavan, S., Lani, R., Huxley, P. and Bennett, G.S. (2020) Identification and Optimization of EphA2-Selective Bicycles for the Delivery of Cytotoxic Payloads. *J Med Chem*, **63**, 4107-4116.

7. Hao, Y., Hao, S., Andersen-Nissen, E., Mauck, W.M., 3rd, Zheng, S., Butler, A., Lee, M.J., Wilk, A.J., Darby, C., Zager, M. *et al.* (2021) Integrated analysis of multimodal single-cell data. *Cell*, **184**, 3573-3587 e3529.

8. Young, M.D. and Behjati, S. (2020) SoupX removes ambient RNA contamination from droplet-based single-cell RNA sequencing data. *Gigascience*, **9**.

9. Choudhary, S. and Satija, R. (2022) Comparison and evaluation of statistical error models for scRNA-seq. *Genome Biol*, **23**, 27.

10. Korsunsky, I., Millard, N., Fan, J., Slowikowski, K., Zhang, F., Wei, K., Baglaenko, Y., Brenner, M., Loh, P.R. and Raychaudhuri, S. (2019) Fast, sensitive and accurate integration of single-cell data with Harmony. *Nat Methods*, **16**, 1289-1296.

11. Marsh, S. (2021) scCustomize: custom visualizations & functions for streamlined analyses of single cell sequencing. *Preprint at https://doi.org/10.5281/zenodo*, **5706430**.

12. Järver, P., Coursindel, T., Andaloussi, S.E., Godfrey, C., Wood, M.J. and Gait, M.J. (2012) Peptide-mediated Cell and In Vivo Delivery of Antisense Oligonucleotides and siRNA. *Mol Ther Nucleic Acids*, **1**, e27.

13. Debets, M.F., van Berkel, S.S., Dommerholt, J., Dirks, A.T., Rutjes, F.P. and van Delft, F.L. (2011) Bioconjugation with strained alkenes and alkynes. *Acc Chem Res*, **44**, 805-815.

14. Gaus, H.J., Gupta, R., Chappell, A.E., Ostergaard, M.E., Swayze, E.E. and Seth, P.P. (2019) Characterization of the interactions of chemically-modified therapeutic nucleic acids with plasma proteins using a fluorescence polarization assay. *Nucleic Acids Res*, **47**, 1110-1122.

15. Schmidt, K., Prakash, T.P., Donner, A.J., Kinberger, G.A., Gaus, H.J., Low, A., Ostergaard, M.E., Bell, M., Swayze, E.E. and Seth, P.P. (2017) Characterizing the effect of GalNAc and phosphorothioate backbone on binding of antisense oligonucleotides to the asialoglycoprotein receptor. *Nucleic Acids Res*, **45**, 2294-2306.

16. Finak, G., McDavid, A., Yajima, M., Deng, J., Gersuk, V., Shalek, A.K., Slichter, C.K., Miller, H.W., McElrath, M.J., Prlic, M. *et al.* (2015) MAST: a flexible statistical framework for assessing transcriptional changes and characterizing heterogeneity in single-cell RNA sequencing data. *Genome Biol*, **16**, 278.
